# Locating and Activating Molecular ‘Time Bombs’: Induction of Mycolata Prophages

**DOI:** 10.1371/journal.pone.0159957

**Published:** 2016-08-03

**Authors:** Zoe A. Dyson, Teagan L. Brown, Ben Farrar, Stephen R. Doyle, Joseph Tucci, Robert J. Seviour, Steve Petrovski

**Affiliations:** 1 La Trobe Institute of Molecular Sciences, Bendigo, VIC, Australia; 2 Department of Animal, Plant & Soil Sciences, Latrobe University, Bundoora, VIC, Australia; 3 Department of Physiology, Anatomy and Microbiology, La Trobe University Bundoora, VIC, Australia; Naval Research Laboratory, UNITED STATES

## Abstract

Little is known about the prevalence, functionality and ecological roles of temperate phages for members of the mycolic acid producing bacteria, the Mycolata. While many lytic phages infective for these organisms have been isolated, and assessed for their suitability for use as biological control agents of activated sludge foaming, no studies have investigated how temperate phages might be induced for this purpose. Bioinformatic analysis using the PHAge Search Tool (PHAST) on Mycolata whole genome sequence data in GenBank for members of the genera *Gordonia*, *Mycobacterium*, *Nocardia*, *Rhodococcus*, and *Tsukamurella* revealed 83% contained putative prophage DNA sequences. Subsequent prophage inductions using mitomycin C were conducted on 17 Mycolata strains. This led to the isolation and genome characterization of three novel *Caudovirales* temperate phages, namely GAL1, GMA1, and TPA4, induced from *Gordonia alkanivorans*, *Gordonia malaquae*, and *Tsukamurella paurometabola*, respectively. All possessed highly distinctive dsDNA genome sequences.

## Introduction

The availability of next generation DNA sequencing has resulted in a dramatic increase in the number of draft and fully annotated bacterial genomes. Yet a neglected area of genomics is that of phages, considering that current estimates place their global abundance at 10^31^ [1, 2]. To date (March 2016), 1,479 actinophage genome sequences have been sequenced, according to the phagesdb database, and of these 1,128 infect members of the genus *Mycobacterium*, making their genomes the most extensively characterized of all phages. Complete genome sequences of phages infective for other mycolic acid producing bacteria, the Mycolata, a distinct evolutionary lineage in the *Actinobacteria*, have been obtained [3–9]. Their hosts include members of the genera *Gordonia*, *Nocardia*, *Rhodococcus*, and *Tsukamurella*. Some share both nucleotide and amino acid sequence similarity with each other, and with some mycobacteriophages [4–6, 8–9].

Bacterial whole genome sequence data have revealed insights into their evolutionary origin, including features that appear to be of phage origin [10–12]. Up to 20% of a bacterial chromosome can be occupied by phages that can contribute to conferring to their parent cells both virulence and resistance to phage infection [10–12]. Lytic cycle induction can occur under a range of conditions, including environmental and anthropogenic perturbations [10–11, 13–17]. Eventual host cell lysis renders any genetic benefit conferred from such an infection transient, while the prophage remains intact [11–12, 14–15].

To our knowledge, most Mycolata phages whose genomes have been sequenced have been isolated by their ability to form plaques on lawn cultures of their hosts [3–7, 9, 18]. These phages are attractive as biocontrol agents to limit proliferation of their host bacteria, including those stabilizing foams in activated sludge reactors [4–6, 19–20].

Prophage induction therapy is particularly appealing for phage biocontrol, especially where lytic phages for a particular bacterium cannot be isolated readily, either because of fastidious host cell growth requirements or phage defense mechanisms encoded by their host, but where complete prophages are integrated into the host genome [21]. Conventional phage therapy requires several stages, including phage isolation, purification, characterization, mass production, determination of appropriate cocktail mixes of phages and development of an appropriate delivery method [22–24].

However, before this approach can be considered for the Mycolata, several key questions need to be addressed. These include the following: (i) Do Mycolata genomes contain prophages? (ii) Is it possible to induce these to undergo their lytic cycles? (iii) Are these phages similar to isolated Mycolata lytic phages, and what can be learned about their evolutionary ancestries? This study attempts to address these questions.

## Materials and Methods

### Mycolata bacterial, plasmid and prophage sequence analyses

Bacterial and plasmid whole genome sequence (wgs) data were obtained from GenBank by searching for the genera of interest by name (*Gordonia*, *Mycobacterium*, *Nocardia*, *Rhodococcus*, and *Tsukamurella*), and those present are described in detail in S1 and S2 Tables. Putative prophage DNA sequence data were detected using PHAST [25], which was preferred over other programs including Prophage Finder [26], Prophinder [27], and Phage finder [28] because of its faster run times, ability to process a wider variety of file types (annotated or un-annotated) of differing completeness (draft or finished) and increased sensitivity [25].

### Bacterial strains and media

Bacterial strains used in this study are listed in Table 1, and in Petrovski et al. [29], and methods for their storage and cultivation were those described by Petrovski et al. [29]. These were all grown on R2A medium (0.5 g/L Yeast extract (Oxoid, Adelaide, Australia), 0.5 g/L Proteose peptone (Difco, North Ryde, Australia), 0.5 g/L Casamino acid (Difco, North Ryde, Australia), 0.5 g/L Glucose, 0.5 g/L soluble starch (Difco, North Ryde, Australia), 0.3 g/L K2HPO4, 0.005 g/L MgSO4.7H2O, 0.3 g/L sodium pyruvate (BDH, Murarrie, Australia)) broth and agar R2A + 14 g/L agar (Oxoid, Adelaide, Australia) at 25 C. All remaining chemicals were obtained from Sigma (Sydney, Australia), unless otherwise noted.

**Table 1:** Summary of strains used in Mycolata prophage induction studies.

Species	Lab ID	Reference	Other ID	Species genome sequences available ^a^	Putative complete prophage(s)	Putative incomplete prophages(s)^b^	Mitomycin C induction	Spontaneous induction
***Dietzia maris***	CON27^T^	[30]	Dmar27, DSMZ 43672	0	NA	NA	-	-
***Gordonia alkanivorans***	CON72^T^	[31]	DSMZ 44369	1	1	0	+	-
***Gordonia amarae***	CON44 ^T^	[32]	Gama44, DSMZ 43392, NBRC 15530	1	0	1	-	-
***Gordonia amarae***	CON9		Gama9, UQCC2810	1	0	1	-	-
***Gordonia amarae***	BEN371			1	0	1	-	-
***Gordonia amarae***	BEN374			1	0	1	-	-
***Gordonia amarae***	BEN381			1	0	1	-	-
***Gordonia amarae***	BEN386			1	0	1	-	-
***Gordonia amarae***	BEN389			1	0	1	-	-
***Gordonia desulfuricans***	CON69 ^T^		213E, NCIMB 40816	0	NA	NA	-	-
***Gordonia polysoprenoraus***	CON71			1	0	2	-	-
***Gordonia malaquae***	G239			1	1	1	+	+
***Tsukamurella paurometabola***	CON55			1	0	1	+	+
***Nocardia brasiliensis***	CON42 ^T^			1	0	0	-	-
***Nocardia brevicatena***	CON43		DSMZ 43024	1	1	1	-	-
***Milisia brevis***	J81 ^T^	[33]	DSMZ 44463	0	NA	NA	-	-
***Milisia brevis***	J82	[33]		0	NA	NA	-	-

^a^ Bacterial strains do not match with sequences in all cases

^b^ Includes those that scored a completeness level of questionable (Q) when screened using PHAST

^T^ Indicates type strain

### Phage induction procedures

Bacterial strains were grown to stationary phase with shaking in either R2A or PYCA broth [33], and then exposed to mitomycin C in attempts to induce any prophages. Rapid screening of multiple concentrations of mitomycin C (0.2, 0.5, 1.0, 2.0, 5.0, 10.0, 15.0 and 20.0 μM) were carried out on 1 mL aliquots of each of the stationary phase bacterial cultures. These cultures were exposed to this range of mitomycin C concentrations overnight, then combined and filtered (0.22 μM pore size) to remove any bacterial cell debris. Filtrates were subjected to DNAse/RNAse pretreatment to remove any bacterial DNA prior to polyethylene glycol (PEG) precipitation to concentrate phage particles. Phage DNA extraction/purification involved proteinase K digestion, and phenol-chloroform-isoamyl alcohol purification. The DNA extracted from mitomycin C treated cells was screened by agarose gel electrophoresis, where presence of a suitably sized DNA band suggested detection of a putative prophage.

Where such a band was observed, separate assays using 1 mL aliquots of stationary phase bacterial cultures and mitomycin C at each of the aforementioned concentrations were then conducted to determine the optimum concentrations of mitomycin C. Similar experiments were conducted on both PYCa or R2A broth and solid media lacking mitomycin C, to screen for any spontaneous prophage induction. Thus, with solid media, lawn plates of the bacterial strains were incubated at 30°C for three days prior to recovering cells into suspension with either PYCa or R2A broth. DNA extraction and agarose gel electrophoresis to detect putative prophages were performed as described above.

### Sequencing and annotation of induced prophage genomes

Phage DNA sequencing libraries were prepared using an Illumina Nextera XT sample preparation kit following the manufacturer’s instructions. The prepared DNA libraries were sequenced on an Illumina MiSeq as a 150-bp paired end run and sequence reads were assembled using CLC Workbench. Open reading frames (*orf*s) within the *de novo* assembled sequences were detected using Glimmer (v3.02), for *orf*s with a minimum size of 90 bp [34]. All predicted start codons were inspected manually for the presence of putative ribosomal binding sites, and corrected if required. Sequence similarity searches were performed against genome sequences in GenBank. The presence of tRNA and tmRNA was sought using ARAGORN [35] and with tRNAScan-SE [36]. Transmembrane domains were predicted with the DAS transmembrane prediction server [37], as described previously [4].

### Preliminary induced phage characterization

Induced phage host ranges were determined by plating a 1:10 dilution series of phage onto host bacterial lawn plates

### Nucleotide sequence accession numbers

The nucleotide sequence for induced prophages GAL1, GMA1, and TPA4 were deposited in GenBank under accession numbers KR053194, KR053195, and KR053196, respectively.

## Results and Discussion

### Prophages are prevalent in Mycolata species

All available whole genome sequence data from *Gordonia*, *Nocardia*, *Rhodococcus*, *Tsukamurella*, *Mycobacterium* strains and their corresponding plasmid sequences were downloaded from the GenBank database on 26^th^ February 2014 (bacterial genome data), and 1^st^ July 2014 (plasmid data). A total of 259 bacterial genomes and 49 plasmid sequences were analyzed. The Mycolata sequences came from isolates from habitats including clinical samples, contaminated soil, activated sludge, faeces, and animals (S1 Table). When these sequences were screened for putative prophage regions, PHAST predicted that 83% of the 259 bacterial genomes may contain genes of phage origin (S1 Table). Of these sequences 26% appeared to contain putative complete intact prophage genomes (S1 Table), ranging from 5.4 to 135.5 kb in size. Some Mycolata genomes contained up to nine putative prophage regions, and up to 4.26% of complete Mycolata genomes was occupied by such regions.

The four Mycolata isolates obtained from wastewater treatment plants whose genomes had been fully sequenced were also examined. These were *Gordonia amarae* strain NBRC 15530, *Gordonia malaquae* strain NBRC 108250, *Gordonia sihwensis* strain NBRC 108236, and *Rhodococcus ruber* strain Chol-4. Of these, only the *R*. *ruber* strain appeared free of genes of phage origin, and only an incomplete putative phage sequence was seen in the *G*. *amarae* genome.

Several of these putative prophage regions showed sequence similarities at an amino acid level to previously characterized Mycolata phages (see S3 Table). For example the *Mycobacterium tuberculosis* strain CCDC5180 genome contained a putative remnant prophage region encoding putative capsid proteins similar to those in *Rhodococcus* phages REQ1 and RRH1 [7, 38]. A small terminase subunit encoding gene was also similar to that in the *Nocardia* phage NBR1 genome [14]. It was also possible to trace the same prophage sequence across genomes of multiple isolates of the same host organism. For example, *Mycobacterium abscessus* isolates 5S-0921 and 5S-1212 both contained an identical intact prophage sequence, 32.7 kb in length.

Forty-nine plasmid sequences were also analyzed from the genera listed above, as Kanda et al [39] had demonstrated that phages can integrate into plasmids. Five Mycolata plasmids were predicted by PHAST to contain intact prophage genomic sequences (S2 Table). All were examined manually, and the genes detected were mainly homologues of those encoding integrases, recombinases, and translocases, suggesting most likely that these were not from prophages. Similar homologues of these genes were observed in some of the bacterial genomic data, making it difficult sometimes to discern if they are genes of true phage origin, as these may also be associated with other mobile genetic elements like plasmids.

### Induction and detection of induced Mycolata prophages

**Fig 1:**
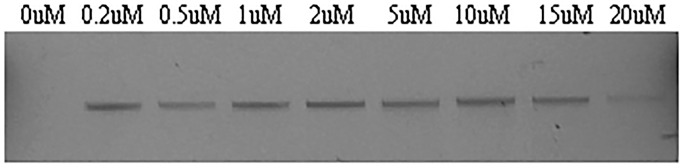
Phage DNA extraction from mitomycin C induced GMA1. Concentration of mitomycin C is indicated on the gel above.

*G*. *alkanivorans* strain CON72, *G*. *malaquae* strain BEN700, and *N*. *brevicatena* strain CON43 all isolated from wastewater treatment plants and held in the La Trobe University culture collection and were used in induction experiments. They were chosen because some whole genome sequence data (see S1 Table), suggested they may contain prophages. So they were used as possible positive controls in the induction experiments. Furthermore, no lytic phages had been recovered for them in earlier work carried out in this laboratory (Petrovski & Seviour, Unpublished data). When these strains were exposed to a range of concentrations of mitomycin C, the prophages GAL1 and GMA1 were successfully induced from *G*. *alkanivorans*, and *G*. *malaquae*, respectively, which is shown in. Fig 1. No prophages were detected in *N*. *brevicatena*, probably because of an absence of a complete prophage in this strain. This result was not surprising as the bacterial strains we used in the induction study are different isolates to those in the bioinformatics analysis. The bioinformatics study was used as a guide to determine if we could detect the presence of any prophage like DNA elements.

Phage GAL1 was inducible at 20 μM mitomycin C, whereas a spontaneous induction of phage GMA1 was also detected in the absence of mitomycin C, with *G*. *malaquae* [40]. When GAL1 phage was examined by TEM, a *Siphoviridae* phage possessing the characteristic long non-contractile tail (~450 nm) and isometric capsid (~75 nm diameter) was seen (data not shown). Examination of other induced phages was unsuccessful, possibly due to low phage titre.

DNA was isolated from both phage GAL1 and GMA1 and their genomes were sequenced and assembled. The data revealed ~927-fold and ~2599-fold average coverage, respectively. GAL1 and GMA1 phages had genome sizes of 49,979 bp and 41,207 bp, and contained 82 and 68 putative *orf*s, respectively. Their genomes contained no tRNA or tmRNA.

Despite the GAL1 phage being induced from a different strain of *G*. *alkanivorans* (CON72) than that examined in the initial whole genome sequence screening (*G*. *alkanivorans* strain NBRC16433), its sequence and that of the NBRC16433 contig GOALK93 (49,954 bp), were >99% homologous. This high level of shared similarity suggests the prophage in strain NBRC16433 may be functional.

### Activating the lytic cycle of prophages in other Mycolata isolates

An additional 14 Mycolata strains (Table 1) whose genomes have not yet been sequenced but which are available in our culture collection, were also exposed to mitomycin C to see if any prophages were present there. These strains included seven *G*. *amarae* strains (Table 1), the most common foaming organism in Australian activated sludge treatment plants [21, 41–43]. However, no prophages were recovered. In one strain of *T*. *paurometabola* (CON55), spontaneous prophage induction was observed, making it difficult to determine in this strain the impact of mitomycin C on phage induction. This phage is referred to as phage TPA4.

### Infection properties of induced prophages

The three phages GAL1, GMA1, and TPA4 were characterized. In host range studies, none formed plaques on any of the Mycolata pure cultures screened. It was clear from the present work that *G*. *alkanivorans* (CON72) cells lysed in order to liberate GAL1 after exposure to 20 μM mitomycin C triggering the SOS response [44].

Despite *T*. *paurometabola* strain CON55 carrying the temperate phage TPA4, co-infection with a different lytic phage TPA2 [3] resulted in plaque formation, suggesting that this phage does not confer immunity to phage TPA2. Subsequent whole genome sequencing confirmed that this TPA2 phage was responsible for generating the plaques, and no TPA4 phage could be detected among the sequence, confirming that TPA4 was not responsible for plaque formation. A similar co-infection of *G*. *malaquae* strain BEN700 was successful with phage GTE2 and confirmed by phage genome sequencing that the plaques produced were by phage GTE2 and not GMA1 [4].

### Temperate phage TPA4 is a previously undescribed phage

**Fig 2:**
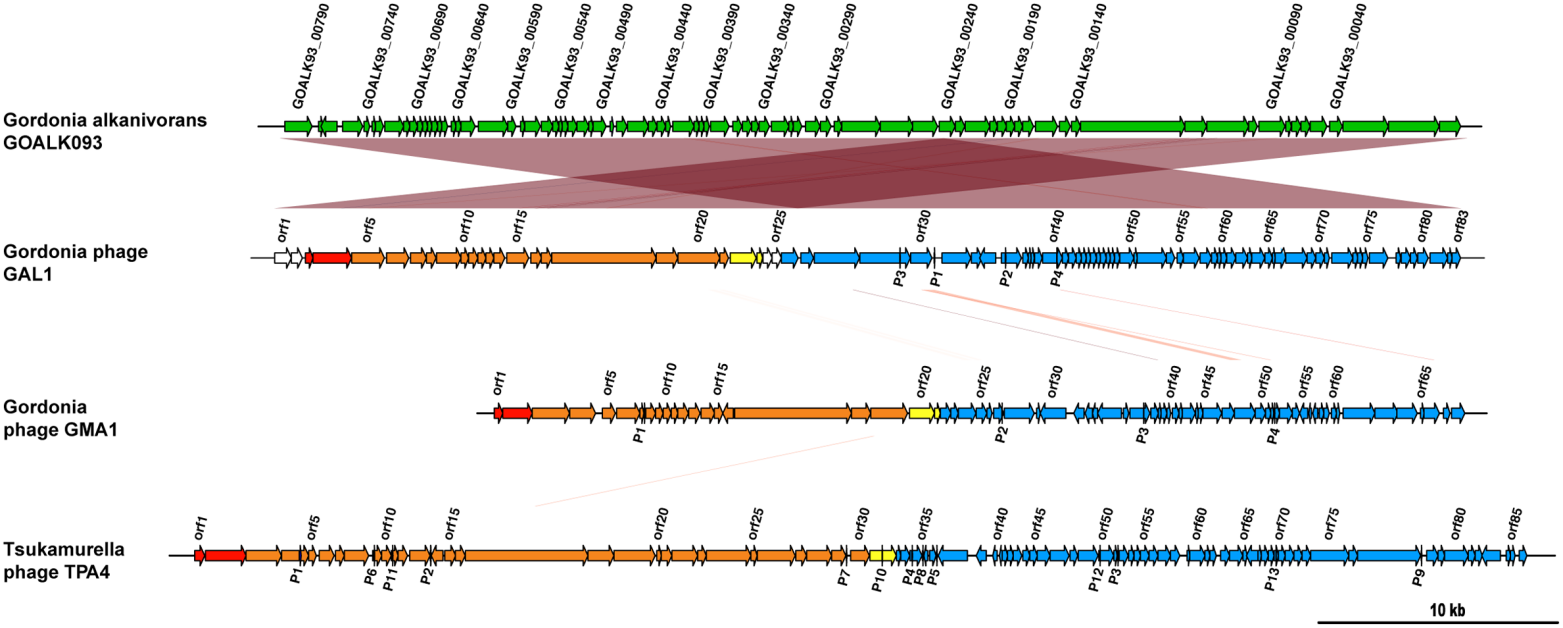
Genome maps of temperate Mycolata phages GAL1, GMA1, and TPA4 and the predicted prophage GOALK093 from the genomes of *G*. *alkanivorans*. The arrows represent the annotated gene and direction of transcription in each genome. Gene modules are similarly shaded i.e., red = encodes DNA packaging proteins, orange = encodes structural proteins, yellow = encodes lysis proteins, blue = encodes phage maintenance proteins. The genome not annotated in this study is shaded in green. Regions of homology are also indicated.

The DNA obtained from the phage TPA4 was sequenced using an Illumina MiSeq platform. Its genome was 56,212 bp in size, with ~1,749-fold average coverage, and contained 84 *orf*s (Fig 2, S3 Table). No putative tRNA or tmRNA could be recognized. It shared a small region (1,825 bp) of nucleotide sequence identity (75%) with *Kineococcus radiotolerans* strain SRS30216. However, no nucleotide sequence similarity was observed with *T*. *paurametabola* strain DSMZ 20162, or to the PHAST predicted prophage region found in its genome, or to the genome of phage TPA2, also infective for this strain [3].

### Sequence repeats are common within the prophage genomes

Several repeat structures were seen in genomes of all three induced prophages. All contained between 4–13 palindromes, ranging in size from 16–86 bp (see S4 Table). Some were located in intergenic regions, where they might act as *rho*-independent transcriptional terminators (see Fig 2) [45]. Between 30–179 direct repeats were found in each of the genome sequences of these three phages (see S5 Table) and ranged in size between 15–193 bp. Furthermore, between 11 and 103 inverted repeats ranging from 15–60 bp in size were detected in all three genomes (see S5 Table).

Inverted repeats can indicate replication origins and transposable elements [46], but neither of these was identified in the induced prophage sequenced here. What functional roles these direct and inverted repeats might play, if any, remains to be determined. However, repeat sequences similar to these have been reported in lytic Mycolata phages [3, 4, 6].

### Summary of features of induced prophage genome sequences

All three temperate phages GAL1, GMA1, and TPA4 had the characteristic modular arrangement distinctive of *Siphoviridae* phages, consisting of encoding DNA packaging genes, structural protein genes, lysis genes, DNA replication genes, and genes associated with lysogeny (Fig 2).

### Phage lysis genes

In each of phage GAL1, GMA1 and TPA4, an N-acetylmuramoyl-L-alanine amidase motif was identified in products of genes *orf22* (pfam01510), *orf20* (pfam01510), and *orf31* (pfam01519), respectively. This motif identifies these as lysis genes, presumably activated at the induction of their lytic cycles. In genomes of phages GAL1 and GMA1 these lysis genes were followed immediately by those encoding putative holin proteins. These holins, identified from amino acid sequence homology, were similar to other putative phage holin proteins. They fulfilled the criteria of Wang et al. [47] that holins should be less than 150 amino acid residues long and contain two or more transmembrane regions. In phage TPA4, no putative holin genes could be identified in the vicinity of the phage lysin gene, suggesting that if present, they are either in an unusual location or have very different amino acid and nucleotide sequences.

### Phage lysogenic conversion and maintenance genes

Integrase genes were identified in all three phage genomes from their amino acid sequence similarities. These were *orf31* in GAL1, *orf30* in GMA1, and *orf*37 in TPA4. Their presence supports an ability of all three to enter a lysogenic replication cycle [48]. In GAL1 phage, a BRO family protein (pfam02498) motif was seen in the N-terminal region of Orf39. The C-terminus contained a phage antirepressor KilAC motif (pfam03374), which may be involved in inactivating phage repressor proteins upon prophage induction [49]. Phage GMA1 also contained a BRO family protein motif (pfam02498) at the N-terminal end of its Orf36. It appears to be encoded by a chimeric gene, as the N-terminal region showed similarity to one in *Mycobacterium avium*, and the C-terminal to that encoded by a gene from *G*. *alkanivorans*. This same region is present in contig GOALK93, which corresponds in its sequence to the Orf34 of phage GAL1. No putative excisionase genes could be found in any of these three phage genome sequences.

Several genes were identified in the GAL1 prophage genome that might confer enhanced fitness to *G*. *alkanivorans*. These included *orf34* whose encoded product contains a motif (pfam09669) for a Rha family regulatory protein. In lamboid phage phi 80, this protein is thought to interfere with phage infection in bacterial strains lacking the host integration host factor (IHF) [50]. The *orf47* of phage GAL1 encodes a protein with high amino acid sequence homology to a putative beta subunit of a protocatechuate 3,4-dioxygenase, involved in degradation of aromatic compounds [51].

### Evolutionary insights into Mycolata prophages

All three induced phages differ to each other at the nucleotide sequence level, and their genomes encode a high proportion of proteins (between 7 to 24%) for which no statistically significant matches in GenBank could be found. However, some amino acid sequence similarity (75% identity, 49% coverage) was seen between the products of *orf34* of GAL1 phage, a putative Rha protein, and *orf36* of GMA1 phage, which is a putative BRO family protein. Furthermore, both phage GMA1 and TPA4 contained genes encoding *G*. *malaquae* protein homologues. Phages GAL1, GMA1, and TPA4 are each genetically unique, and are therefore placed in the category of ‘singletons’ in accordance to Hatfull et al [52], as they share no phylogenetic relationships with any other phages included there.

Despite this, GMA1 possessed several genes whose encoded products had amino acid sequence similarities to those seen in *Mycobacterium* phages Mutaform13, Ramsey, Gumball, PMC, and Dori. This finding suggests some shared evolutionary ancestry between these phages and the GMA1 phage. Furthermore, the GMA1 genome contained many genes that encode amino acid sequences with similarities to several hypothetical proteins in *Rhodococcus spp*., *G*. *malaquae*, and *G*. *sihwensis*.

## Conclusions

*In silico* whole genome sequence data analyses suggest that prophages occur commonly in the Mycolata, identifying many potentially useful molecular ‘time bombs’, to be activated *en masse* to target these organisms and hence control biologically any problems they cause, including the formation of stable foams in wastewater treatment plants.

However, several questions need to be addressed before such a strategy is considered. For example, exposure to any possible agent or factor inducing their lytic cycles might in turn activate the lytic cycle in other temperate phages whose host cells are functionally important bacteria in activated sludge communities. Preliminary induction studies have suggested that some of the lysogenic phages described here are probably functional and can be induced into their lytic cycles. Their genomes reveal genes that may provide their host cells with advantageous attributes, which may be ecologically important. Prophage induction therapy could be an attractive approach to targeting hosts such as the Mycolata with multiple phage defense systems where these are infected with temperate phages, or where the bacterium in question is difficult to culture in a laboratory, thus making lytic phage isolation difficult.

## Supporting Information

S1 Table Putative prophages detected in publically available GenBank Mycolata wgs data using PHAST. (PDF)

S2 Table Prophages detected in plasmid sequences using PHAST. (PDF)

S3 Table GAL1, GMA1 and TPA4 geneome sequence annotations. (PDF)

S4 Table Palindromes in the genome sequences of phages GAL1, GMA1 and TPA4. (PDF)

S5 Table Repeats in the genome sequences of phages GAL1, GMA1 and TPA4. (PDF)
