# Supplementary material for: Locating and Activating Molecular ‘Time Bombs’: Induction of Mycolata Prophages
Source: PLoS One. 2016 Aug 3;11(8):e0159957. doi: 10.1371/journal.pone.0159957 (PMC4972346; doi:10.1371/journal.pone.0159957)
Supplement: S1 Table — (PDF) [file pone.0159957.s001.pdf]

S1 Table: Putative prophages detected in publically available GenBank Mycolata wgs data using PHAST

Sequence number	Organism	Isolation source [c]	Accession/GI number	No. putative prophage region(s)	Percentage of genome encoding putative prophage region(s)[e]	Coordinates within host genome [a]	Size (kb)	Intact [b]	No. CDS	G+C mol % of prophage region
**1**	*Gordonia aichiensis* NBRC 108223 [d]	Human sputum, Japan	NZ_BANR01000000	1	N.A.	3917277-3992148	74.8	Y	63	65.53
**2**	*Gordonia alkanivorans* NBRC 16433 [d]	Tar and phenol contaminated soil, Germany	NZ_BACI01000000	1	N.A.	3524537-3578378	53.8	Y	77	63.84
**3**	*Gordonia amarae* NBRC 15530 [d]	Abnormal foam on activated sludge, USA	NZ_BAED01000000	1	N.A.	299556-307233	7.6	N	10	65.67
**4**	*Gordonia amicalis* NBRC 100051 [d]	Garden soil, Russian Federation	NZ_BANS00000000	0	N.A.					
**5**	*Gordonia araii* NBRC 100433 [d]	Human, Japan	NZ_BAEE00000000	2	N.A.	1392152-1398883	6.7	N	8	65.30
						3895971-3904239	8.2	Q	10	66.31
**6**	*Gordonia bronchialis* DSM 43247 [f]	Sputum, Human with cavitary disease of both upper lungs	NC_013441	2	0.91 %	402787-428773	25.9	Y	22	63.97
						1230465-1251773	21.3	N	15	63.38
**7**	*Gordonia effusa* NBRC 100432 [d]	Human, Japan	NZ_BAEH00000000	0	N.A.					
**8**	*Gordonia hirsuta* DSM 44140 (NBRC 16056) [d]	Packing material of a bio-filter	NZ_BANT00000000	0	N.A.					
**9**	*Gordonia* sp. KTR9 [f]	Soil from an explosives testing facility	NC_018581	2	1.18 %	1517766-1570506	52.7	N	48	66.10
						3874442-3885732	11.2	N	7	62.93
**10**	*Gordonia malaquae* NBRC 108250 [d]	Sludge of a wastewater treatment plant, Taiwan	NZ_BAOP00000000	2	N.A.	3536151-3573410	37.2	Y	45	65.84
						4006158-4019973	13.8	N	23	65.79
**11**	*Gordonia namibiensis* NBRC 108229 [d]	Soil, Namibia	NZ_BAHE00000000	1	N.A.	3539990-3546762	6.7	N	7	68.73
**12**	*Gordonia neofelifaecis* NRRL B-59395 [d]	Faeces, China	NZ_AEUD00000000	2	N.A.	441982-458783	16.8	N	23	67.86
						4198346-4216288	17.9	Q	28	67.94
**13**	*Gordonia otitidis* NBRC 100426 [d]	Human, Japan	NZ_BAFB00000000	3	N.A.	4243486-4248947	5.4	N	7	66.64
						4404497-4430217	25.7	N	20	63.48
						4658711-4673172	14.4	N	9	63.34
**14**	*Gordonia paraffinivorans* NBRC 108238 [d]	Water sample from oil-producing well, China	NZ_BAOQ00000000	0	N.A.					
**15**	*Gordonia polyisoprenivorans* NBRC 16320 [d]	Fouling tire water, Germany	NZ_BAEI00000000	2	N.A.	1472324-1482288	9.9	Q	9	63.67
						2332996-2341150	8.1	Q	10	62.19
**16**	*Gordonia polyisoprenivorans* VH2 (DSMZ 44266) [f]	Earth of rubber tree plantation, Vietnam	NC_016906	2	0.51 %	1456870-1472745	15.8	Y	19	62.43
						1564965-1578231	13.2	N	12	63.57
**17**	*Gordonia rhizosphera* NBRC 16068 [d]	Root, rhizosphere of mangrove trees, Japan	NZ_BAHC00000000	0	N.A.					
**18**	*Gordonia rubripertincta* NBRC 101908 [d]	Soil, Japan	NZ_BAHB00000000	1	N.A.	594440-601941	7.5	Q	6	62.48
**19**	*Gordonia sihwensis* NBRC 108236 [d]	Wastewater-treatment bioreactor, Korea	NZ_BANU00000000	6	N.A.	274177-286634	12.4	N	17	67.92
						285658-319974	34.3	Q	20	67.48
						1653943-1693179	39.2	Y	59	64.32
						3362150-3376889	14.7	N	17	64.22
						3615805-3635013	19.2	N	13	66.99
						3666347-3680269	13.9	N	7	70.68
**20**	*Gordonia soli* NBRC 108243 [d]	Soil, Taiwan	NZ_BANX00000000	4	N.A.	4903248-4944450	41.2	Y	41	67.99
						4980403-4999965	19.5	N	32	68.15
						5225127-5255781	30.6	Q	36	66.33
						5328847-5359588	30.7	N	37	67.35
**21**	*Gordonia sputi* NBRC 100414 [d]	Sputa of patients with pulmonary disease	NZ_BAFC00000000	1	N.A.	2579797-2589231	9.4	N	11	60.46
**22**	*Gordonia terrae* NBRC 100016 [d]	Soil, Japan	NZ_BAFD00000000	1	N.A.	3548749-3558477	9.7	N	8	61.87
**23**	*Mycobacterium abscessus subsp. bolletii* 50594[f]	N.D.	NC_021282	5	4.26 %	772815-828935	56.1	Y	41	63.62

Sequence number	Organism	Isolation source [c]	Accession/GI number	No. putative prophage region(s)	Percentage of genome encoding putative prophage region(s)[e]	Coordinates within host genome [a]	Size (kb)	Intact [b]	No. CDS	G+C mol % of prophage region
						822655-845576	22.9	N	30	63.22
						1573263-1644763	71.5	Y	96	60.23
						1854758-1863909	9.1	N	10	65.94
						3433590-3486892	53.3	Y	75	63.04
**24**	*Mycobacterium abscessus* ATCC 19977	N.D.	NC_010397	1	0.91 %	1782165-1828386	46.2	Y	39	59.91
**25**	*Mycobacterium abscessus* 3A-0119-R [d]	Sputum, Human	NZ_AKUX00000000	8	N.A.	109885-164646	54.7	Y	66	63.91
						407161-452128	44.9	Y	63	63.86
						1453952-1463715	9.7	N	15	59.09
						1477659-1520948	43.2	Y	50	60.47
						1577669-1595325	17.6	N	20	61.29
						2991215-3035148	43.9	Y	61	64.67
						3933832-3955847	22	N	26	64.07
						3952174-3968978	16.8	N	18	63.97
**26**	*Mycobacterium abscessus* 3A-0122-R [d]	Sputum, Human	NZ_AKUY00000000	5	N.A.	514740-555566	40.8	Y	57	63.78
						1513575-1523338	9.7	N	16	59.09
						1537283-1580457	43.1	Y	52	60.47
						1651790-1669446	17.6	N	20	61.28
						3968888-4053927	85	Y	121	64.23
**27**	*Mycobacterium abscessus* 3A-0122-S [d]	Sputum, Human	NZ_AKUZ00000000	9	N.A.	241418-281439	40	Y	56	63.78
						1065906-1075669	9.7	N	15	59.09
						1077967-1111407	33.4	Y	36	60.41
						1181409-1199065	17.6	N	20	61.28
						1482620-1493497	10.8	N	17	61.24
						2611951-2647689	35.7	Y	39	64.70
						3479109-3496213	17.1	N	29	63.37
						3769725-3793752	24	N	30	63.72
						3790079-3806883	16.8	N	18	63.97
**28**	*Mycobacterium abscessus* 3A-0731 [d]	Sputum, Human	NZ_AKVA00000000	8	N.A.	109901-165856	55.9	Y	66	63.90
						1079592-1114497	34.9	Y	47	63.94
						1423191-1432954	9.7	N	14	59.09
						1446899-1490073	43.1	Y	49	60.47
						1546794-1564450	17.6	N	20	61.28
						3156535-3187637	31.3	Y	39	64.70
						4019064-4061249	42.1	Q	63	64.01
						4057576-4074380	16.8	N	18	63.97
**29**	*Mycobacterium abscessus* 3A-0810-R [d]	Bronchial alveolar lavage, Human	NZ_AKUP00000000	9	N.A.	109837-165791	55.9	Y	67	63.90
						407636-446855	39.2	Y	58	63.75
						1509148-1518911	9.7	N	16	59.09
						1521209-1554840	33.6	Y	38	60.41
						1611561-1629059	17.4	N	21	61.30
						1753320-1782585	29.2	N	16	62.98
						3045844-3087591	41.7	Y	59	64.73
						3919086-3962282	43.1	Q	63	64.06
						3958609-3975422	16.8	N	18	63.96
**30**	*Mycobacterium abscessus* 3A-0930-R [d]	Sputum, Human	NZ_AKVB00000000	7	N.A.	109877-165831	55.9	Y	67	63.90
						407847-449145	41.2	Y	58	63.79
						1506729-1516492	9.7	N	15	59.09
						1529492-1572666	43.1	Y	50	60.47
						1629387-1647043	17.6	N	20	61.29

Sequence number	Organism	Isolation source [c]	Accession/GI number	No. putative prophage region(s)	Percentage of genome encoding putative prophage region(s)[e]	Coordinates within host genome [a]	Size (kb)	Intact [b]	No. CDS	G+C mol % of prophage region
						3887720-3931599	43.8	Y	58	63.81
						5244818-5273535	28.7	Y	39	64.91
**31**	*Mycobacterium abscessus* 3A-0930-S [d]	Sputum, Human	NZ_AKVC00000000	8	N.A.	109889-165843	55.9	Y	67	63.90
						1118306-1128069	9.7	N	15	59.09
						1142014-1185188	43.1	Y	50	60.47
						1241909-1259565	17.6	N	20	61.28
						2206800-2248098	41.2	Y	58	63.78
						2990072-3004926	14.8	N	23	64.15
						3835701-3879144	43.4	Y	57	63.85
						5235152-5249987	14.8	Q	25	65.58
**32**	*Mycobacterium abscessus* 4S-0116-R [d]	Sputum, Human	NZ_AKVD00000000	1	N.A.	3229344-3281457	51.2	Y	73	63.21
**33**	*Mycobacterium abscessus* 4S-0116-S [d]	Sputum, Human	NZ_AKVE00000000	1	N.A.	2294181-2347641	53.4	Y	75	63.15
**34**	*Mycobacterium abscessus* 4S-0206 [d]	Sputum, Human	NZ_AKUT00000000	1	N.A.	3236939-3290399	53.4	Y	74	63.15
**35**	*Mycobacterium abscessus* 4S-0303 [d]	Sputum, Human	NZ_AKTU00000000	1	N.A.	3218556-3273405	54.8	Y	77	63.09
**36**	*Mycobacterium abscessus* 4S-0726-RA [d]	Sputum, Human	NZ_AKTV00000000	1	N.A.	3155792-3210641	54.8	Y	77	63.09
**37**	*Mycobacterium abscessus* 4S-0726-RB [d]	Sputum, Human	NZ_AKTW00000000	1	N.A.	2746347-2799807	53.4	Y	74	63.15
**38**	*Mycobacterium abscessus* 5S-0304 [d]	Sputum, Human	NZ_AKTX00000000	4	N.A.	2613910-2642403	28.4	N	29	63.20
						2630693-2663407	32.7	Y	45	63.54
						3151707-3193917	42.2	Q	62	63.92
						3193933-3206308	12.3	N	18	64.66
**39**	*Mycobacterium abscessus* 5S-0421 [d]	Sputum, Human	NZ_AKTY00000000	5	N.A.	2885728-2914221	28.4	N	29	63.20
						2902511-2935225	32.7	Y	45	63.54
						3423525-3465735	42.2	Q	62	63.92
						3465751-3478261	12.5	N	18	64.46
						4449383-4474591	25.2	Q	21	60.46
**40**	*Mycobacterium abscessus* 5S-0422 [d]	Sputum, Human	NZ_AKTZ00000000	4	N.A.	3777961-3806454	28.4	N	29	63.20
						3794744-3827458	32.7	Y	45	63.54
						4315696-4357906	42.2	Q	62	63.92
						4357922-4370432	12.5	N	18	64.46
**41**	*Mycobacterium abscessus* 5S-0708 [d]	Sputum, Human	NZ_AKUA00000000	5	N.A.	2659111-2687604	28.4	N	29	63.20
						2675894-2708608	32.7	Y	45	63.54
						3196846-3239056	42.2	Q	62	63.92
						3239072-3251582	12.5	N	18	64.46
						4242372-4267580	25.2	Q	21	60.46
**42**	*Mycobacterium abscessus* 5S-0817 [d]	Lung autopsy, Human	NZ_AKUB00000000	4	N.A.	2227716-2256209	28.4	N	29	63.20
						2244499-2277213	32.7	Y	45	63.54
						2765451-2807661	42.2	Q	62	63.92
						2807677-2820187	12.5	N	18	64.46
**43**	*Mycobacterium abscessus* 5S-0921 [d]	Lymph node biopsy, Human	NZ_AKUQ00000000	5	N.A.	3561816-3590309	28.4	N	29	63.20
						3578599-3611313	32.7	Y	45	63.54
						4097750-4139960	42.2	Q	62	63.92
						4139976-4152351	12.3	N	18	64.66
						5143650-5168858	25.2	Q	21	60.46
**44**	*Mycobacterium abscessus* 5S-1212 [d]	Sputum, Human	NZ_AKUC00000000	5	N.A.	2387334-2415827	28.4	N	30	63.20
						2404117-2436831	32.7	Y	45	63.54
						2925068-2967278	42.2	Q	61	63.92
						2967294-2979804	12.5	N	18	64.46
						3970653-3995861	25.2	Q	21	60.46
**45**	*Mycobacterium abscessus* 5S-1215 [d]	Sputum, Human	NZ_AKUD00000000	3	N.A.	543915-599790	55.8	Y	78	64.32

Sequence number	Organism	Isolation source [c]	Accession/GI number	No. putative prophage region(s)	Percentage of genome encoding putative prophage region(s)[e]	Coordinates within host genome [a]	Size (kb)	Intact [b]	No. CDS	G+C mol % of prophage region
						2682084-2710577	28.4	N	29	64.20
						2698867-2731581	32.7	Y	45	63.54
**46**	*Mycobacterium abscessus* 6G-0125-R [d]	Sputum, Human	NZ_AKUE00000000	3	N.A.	66052-120303	54.2	Y	68	64.38
						802919-841094	38.1	Q	36	60.12
						4557643-4575474	17.8	N	10	60.80
**47**	*Mycobacterium abscessus* 6G-0125-S [d]	Sputum, Human	NZ_AKUF00000000	4	N.A.	547349-565279	17.9	N	11	60.78
						1197620-1251871	54.2	Y	69	64.38
						1915250-1925824	10.5	N	21	58.21
						1935012-1973358	38.3	Q	35	60.11
**48**	*Mycobacterium abscessus* 6G-0212 [d]	Sputum, Human	NZ_AKUR00000000	3	N.A.	420248-438178	17.9	N	10	60.78
						1070515-1124766	54.2	Y	68	64.38
						1807596-1845939	38.3	Q	36	60.11
**49**	*Mycobacterium abscessus* 6G-0728-R [d]	Sputum, Human	NZ_AKUS00000000	3	N.A.	548967-566897	17.9	N	10	60.78
						1199246-1253497	54.2	Y	69	64.38
						1936410-1974756	38.3	Q	36	60.11
**50**	*Mycobacterium abscessus* 6G-0728-S [d]	Sputum, Human	NZ_AKUG00000000	3	N.A.	230291-248122	17.8	N	10	60.80
						929020-967363	38.3	Q	36	60.11
						1548133-1602384	54.2	Y	69	64.38
**51**	*Mycobacterium abscessus* 6G-1108 [d]	Sputum, Human	NZ_AKUH00000000	4	N.A.	550282-568212	17.9	N	10	60.78
						1197986-1252237	54.2	Y	70	64.38
						1915504-1926078	10.5	N	21	58.21
						1935269-1973612	38.3	Q	36	60.11
**52**	*Mycobacterium abscessus* 47J26 [d]	Sputum sample from CF patient, Human, United Kingdom	NZ_AGQU00000000	3	N.A.	1760597-1782369	21.7	N	27	60.53
						1776946-1799973	23	N	14	60.48
						2823468-2877820	54.3	Y	74	64.10
**53**	*Mycobacterium abscessus* 9808 [d]	Wound of exudate of postoperative patients, Human, China	NZ_ANAR00000000	2	N.A.	4294238-4344432	50.1	Y	70	64.06
						5059013-5122817	63.8	N	76	63.64
**54**	*Mycobacterium abscessus* subsp. bolletii BD (DSMZ 45149) [d]	Broncho-alveolar liquid, France	NZ_AHAS00000000	1	N.A.	4299517-4371046	71.5	Q	85	63.68
**55**	*Mycobacterium abscessus* M24 [d]	Broncho-alveolar lavage sample from patient with lung infection, Human, Malaysia	NZ_AJLY00000000	7	N.A.	187423-202814	15.3	N	18	62.04
						413950-443162	29.2	Y	40	63.58
						1055788-1112837	57	Y	62	63.53
						2302724-2320245	17.5	N	17	55.50
						2936437-2952326	15.8	Q	20	64.72
						4658246-4674037	15.7	N	20	61.14
						5328152-5343522	15.3	N	22	61.60
**56**	*Mycobacterium abscessus* M93 [d]	Sputum of a patient with lung infections, Human, Malaysia	NZ_AJGF00000000	4	N.A.	3300104-3338963	38.3	Y	43	63.84
						3352404-3373412	21	N	41	63.17
						4133118-4185793	52.6	Q	63	62.85
						4188228-4207912	19.6	N	25	61.88
**57**	*Mycobacterium abscessus* M94 [d]	Sputum of a patient with lung infections, Human	NZ_AJGG00000000	2	N.A.	1138860-1202845	63.9	Y	72	64.35
						3656790-3750803	94	Q	100	58.06
**58**	*Mycobacterium abscessus* M115 [d]	Sputum sample from a patient with lung infection, Human, Malaysia	NZ_AJLZ00000000	3	N.A.	1531354-1554355	23	N	17	62.29
						3069333-3155505	86.1	Y	100	63.19
						4808660-4867675	59	N	81	63.91
**59**	*Mycobacterium abscessus* M139 [d]	Sputum sample from patient with lung infection, Human, Malaysia	NZ_AKVR00000000	2	N.A.	2893239-2942215	48.9	Y	58	64.78
						5034033-5046263	12.2	Y	16	54.62
**60**	*Mycobacterium abscessus* M148 [d]	Sputum sample from patient with lung infection, Human, Malaysia	NZ_AKVV00000000	9	N.A.	211977-247242	35.2	N	51	64.33
						409941-428623	18.6	N	32	56.40

Sequence number	Organism	Isolation source [c]	Accession/GI number	No. putative prophage region(s)	Percentage of genome encoding putative prophage region(s)[e]	Coordinates within host genome [a]	Size (kb)	Intact [b]	No. CDS	G+C mol % of prophage region
						451544-473404	21.8	N	9	61.92
						535393-556169	20.7	N	25	64.98
						1624001-1648412	24.4	N	21	63.39
						2722788-2758053	35.2	N	21	63.30
						3371898-3389510	17.6	N	19	64.87
						4166366-4194351	27.9	N	40	63.75
						4696682-4717506	20.8	N	37	63.24
**61**	*Mycobacterium abscessus* M152 [d]	Sputum sample from patient with lung infection, Human, Malaysia	NZ_AKVT00000000	3	N.A.	1897722-1912440	14.7	N	25	64.10
						4781005-4806057	25	N	31	63.95
						4806073-4818805	12.7	N	17	64.37
**62**	*Mycobacterium abscessus* M154 [d]	Sputum sample from a patient with lung infection, Human, Malaysia	NZ_AJMA00000000	1	N.A.	1245061-1267131	22	N	23	61.29
**63**	*Mycobacterium abscessus* M156 [d]	Sputum sample from patient with lung infection, Human, Malaysia	NZ_AKVU00000000	5	N.A.	442783-481816	39	N	34	63.87
						2195989-2235859	39.8	Y	48	63.84
						2367196-2389266	22	N	23	61.29
						4435202-4487053	51.8	N	62	64.12
						5009680-5020998	11.3	Q	18	55.38
**64**	*Mycobacterium abscessus* M159 [d]	Sputum sample from a patient with lung infection, Human, Malaysia	NZ_AJSD00000000	0	N.A.					
**65**	*Mycobacterium abscessus* M172 [d]	Sputum sample from a patient with lung infection, Human, Malaysia	NZ_AJSE00000000	9	N.A.	1558332-1580402	22	N	25	61.29
						1837893-1866432	28.5	N	33	63.87
						2714489-2730476	15.9	N	21	61.16
						2734183-2748188	14	N	25	58.30
						2792748-2809309	16.5	N	25	56.80
						2813622-2846708	33	Y	21	58.58
						3381549-3436748	55.2	N	74	64.04
						3425180-3476134	50.9	Y	58	63.48
						4473837-4530262	56.4	Q	67	63.77
**66**	*Mycobacterium abscessus* CF [d]	N.D.	NZ_CAHZ00000000	1	N.A.	3000231-3051357	51.1	Y	62	64.60
**67**	*Mycobacterium africanum* GM041182	N.D.	NC_015758	1	0.17 %	1154358-1161894	7.5	N	11	65.25
**68**	*Mycobacterium avium* 104	N.D.	NC_008595	4	2.02 %	746437-795760	49.3	N	64	66.79
						1178359-1197714	19.3	N	23	66.53
						1429012-1464399	35.3	Y	24	66.79
						2934861-2949403	14.5	N	13	69.50
**69**	*Mycobacterium avium* subsp. avium ATCC 25291 [d]	Liver of diseased hen	NZ_ACFI00000000	2	N.A.	2474391-2498352	23.9	N	25	69.48
						4417447-4426114	8.6	N	7	70.18
**70**	*Mycobacterium avium* subsp. paratuberculosis JQ5 [d]	*Camelus dromedarius*	NZ_AHAZ00000000	0	N.A.					
**71**	*Mycobacterium avium* subsp. paratuberculosis JQ6 [d]	*Camelus dromedarius*	NZ_AHBA00000000	1	N.A.	3161127-3192743	31.6	N	22	67.28
**72**	*Mycobacterium avium* subsp. paratuberculosis K-10	Faeces, animal, United States	NC_002944	0	0.00 %					
**73**	*Mycobacterium avium* subsp. paratuberculosis MAP4	Faeces, animal, United States	NC_021200	1	0.65%	4258270-4289551	31.2	N	10	68.20
**74**	*Mycobacterium avium* subsp. paratuberculosis S5 [d]	Terminally sick Jamunapari goat, India	NZ_ANPD00000000	1	N.A.	2875542-2885533	9.9	N	8	69.20
**75**	*Mycobacterium avium* subsp. paratuberculosis S397 [d]	Suffolk breed of sheep with Johne's disease, United States	NZ_AFIF00000000	0	N.A.					
**76**	*Mycobacterium bovis* AF2122/97 (ATCC BAA-	Tuberculous cow with caseous lesions	NC_002945	1	0.48 %	1753159-1773871	20.7	N	22	66.10

Sequence number	Organism	Isolation source [c]	Accession/GI number	No. putative prophage region(s)	Percentage of genome encoding putative prophage region(s)[e]	Coordinates within host genome [a]	Size (kb)	Intact [b]	No. CDS	G+C mol % of prophage region
	935)	in lung and bronchomediastinal lymph nodes, England, United Kingdom								
**77**	*Mycobacterium bovis* BCG str. Korea 1168P	N.D.	NC_020245	1	0.18 %	1158781-1166317	7.5	N	11	65.21
**78**	*Mycobacterium bovis* BCG str. Mexico	N.D.	NC_016804	0	0.00 %					
**79**	*Mycobacterium bovis* BCG str. Pasteur 1173P2	N.D.	NC_008769	0	0.00 %					
**80**	*Mycobacterium bovis* BCG str. Tokyo 172	N.D.	NC_012207	0	0.00 %					
**81**	Mycobacterium canettii CIPT 140010059	N.D.	NC_015848	2	0.56 %	1175143-1191934	16.7	N	11	64.40
						3132808-3141183	8.3	N	9	66.18
**82**	*Mycobacterium canettii* CIPT 140060008	N.D.	NC_019950	2	0.91 %	2359232-2379642	20.4	Y	21	65.94
						3425584-3445682	20	N	9	64.61
**83**	*Mycobacterium canettii* CIPT 140070002 [d]	N.D.	NZ_CAOL00000000	0	N.A.					
**84**	*Mycobacterium canettii* CIPT 140070005 [d]	N.D.	NZ_CAOM00000000	1	N.A.	3932695-3940991	8.2	N	8	67.63
**85**	*Mycobacterium canettii* CIPT 140070007 [d]	N.D.	NZ_CAOO00000000	1	N.A.	912000-943297	31.2	Q	40	63.57
**86**	*Mycobacterium canettii* CIPT 140070008	N.D.	NC_019965	1	0.38 %	2379516-2396325	16.8	N	21	65.73
**87**	*Mycobacterium canettii* CIPT 140070010	N.D.	NC_019951	1	0.24 %	1511064-1521712	10.6	N	10	65.16
**88**	*Mycobacterium canettii* CIPT 140070013 [d]	N.D.	NZ_CAON00000000	0	N.A.					
**89**	*Mycobacterium canettii* CIPT 140070017	N.D.	NC_019952	0	0.00 %					
**90**	*Mycobacterium chubuense* NBB4 [f]	Creosote-contaminated soil	NC_018027	0	0.00 %					
**91**	*Mycobacterium colombiense* CECT 3035 (DSMZ 45105) [d]	Blood of a HIV positive patient, South America	NZ_AFVW00000000	2	N.A.	346288-381299	35	N	13	68.96
						3425806-3442217	16.4	N	17	69.13
**92**	*Mycobacterium fortuitum* subsp. fortuitum DSM 46621 [d]	Cold abscess, Human	NZ_ALQB00000000	3	N.A.	2258394-2279587	21.1	N	21	66.07
						2965733-2997490	31.7	N	8	64.30
						3154534-3184955	30.4	N	13	64.87
**93**	*Mycobacterium gilvum* PYR-GCK [f]	N.D.	NC_009338	0	0.00 %					
**94**	*Mycobacterium gilvum* Spyr1 [f]	Creosote-contaminated soil, Greece	NC_014814	2	1.09 %	2607245-2644937	37.6	Q	14	62.06
						4767385-4789945	22.5	N	9	67.40
**95**	*Mycobacterium hassiacum* DSM 44199 [d g]	Urine, Germany	NZ_AMRA00000000	4	N.A.	1309867-1331196	21.3	N	7	68.21
						2225906-2244099	18.1	N	7	69.78
						2949565-3006543	56.9	Y	83	63.51
						4283332-4297497	14.1	N	7	69.13
**96**	*Mycobacterium hassiacum* DSM 44199 [d g]	Urine, Germany	NZ_ARBU00000000	2	N.A.	3237548-3292409	54.8	Q	82	62.99
						3312556-3321918	9.3	N	8	70.31
**97**	*Mycobacterium indicus* pranii MTCC 9506 (DSMZ 45239)	Animal, South India	NC_018612	2	0.63 %	3134984-3158732	23.7	Q	15	64.13
						4731466-4743160	11.6	N	10	65.42
**98**	*Mycobacterium intracellulare* ATCC 13950 [g]	Clinical isolates from a Korean pulmonary patient, male, 64 years of age, Human, Korea	NC_016946	0	0.00 %					
**99**	*Mycobacterium intracellulare* ATCC 13950 [d g]	Human lymph node	NZ_ABIN00000000	0	N.A.					
**100**	*Mycobacterium intracellulare* MOTT-02	Clinical isolates from a Korean pulmonary patient, male, 64 years of age, Human, Korea	NC_016947	1	0.61 %	792142-824918	32.7	N	17	65.17
**101**	*Mycobacterium intracellulare* MOTT-64	Clinical isolates from a Korean pulmonary patient, male, 64 years of age, Human, Korea	NC_016948	2	0.67 %	811269-829827	18.5	N	18	65.29
						1064478-1082928	18.4	N	9	66.85
**102**	*Mycobacterium kansasii* ATCC 12478 [f g]	From a fatal case	NC_022663	3	0.62 %	1384178-1395201	11	N	9	67.04
						2874153-2884766	10.6	N	14	65.42
						3758746-3776831	18	Q	18	64.33

Sequence number	Organism	Isolation source [c]	Accession/GI number	No. putative prophage region(s)	Percentage of genome encoding putative prophage region(s)[e]	Coordinates within host genome [a]	Size (kb)	Intact [b]	No. CDS	G+C mol % of prophage region
**103**	*Mycobacterium kansasii* ATCC 12478 [d f g]	From a fatal case	NZ_ACBV00000000	4	N.A.	4411548-4426164	14.6	N	23	64.10
						5358572-5370007	11.4	N	14	64.82
						6048284-6056911	8.6	N	11	62.87
						6376434-6384682	8.2	N	10	63.69
**104**	*Mycobacterium leprae* Br4923	Brazil	NC_011896	0	0.00 %					
**105**	*Mycobacterium leprae* TN	N.D.	NC_002677	0	0.00 %					
**106**	*Mycobacterium liflandii* 128FXT [f]	N.D.	NC_020133	2	0.30 %	4386145-4395998	9.8	N	7	64.23
						4979523-4988579	9	N	10	65.25
**107**	*Mycobacterium marinum* M (ATCC BAA-535) [f]	Tissue samples of human lesions, United States	NC_010612	2	0.46 %	1764630-1771903	7.2	N	10	64.70
						4825093-4848031	22.9	N	26	63.15
**108**	*Mycobacterium massiliense* 1S-151-0930 [d]	Bronchial alveolar lavage, Human	NZ_AKUI00000000	1	N.A.	2531963-2597196	65.2	Q	87	63.69
**109**	*Mycobacterium massiliense* 1S-152-0914 [d]	Bronchial alveolar lavage, Human	NZ_AKUJ00000000	1	N.A.	3553849-3619082	65.2	Q	87	63.69
**110**	*Mycobacterium massiliense* 1S-153-0915 [d]	Sputum, Human	NZ_AKUK00000000	1	N.A.	2890488-2955721	65.2	Q	87	63.69
**111**	*Mycobacterium massiliense* 1S-154-0310 [d]	Bronchial alveolar lavage, Human	NZ_AKUL00000000	1	N.A.	2902042-2967275	65.2	Q	87	63.69
**112**	*Mycobacterium massiliense* 2B-0107 [d]	Sputum, Human	NZ_AKUN00000000	2	N.A.	313492-346778	33.2	Q	58	59.76
						346833-361617	14.7	N	16	60.04
**113**	*Mycobacterium massiliense* 2B-0307 [d]	Sputum, Human	NZ_AKUU00000000	1	N.A.	317738-346567	28.8	N	47	60.03
**114**	*Mycobacterium massiliense* 2B-0626 [d]	Bronchial alveolar lavage, Human	NZ_AKUM00000000	2	N.A.	973572-1021697	48.1	Q	74	59.85
						2683294-2713268	29.9	N	16	59.65
**115**	*Mycobacterium massiliense* 2B-0912-R [d]	Sputum, Human	NZ_AKUV00000000	2	N.A.	1391796-1425082	33.2	Q	58	59.76
						1425137-1439921	14.7	N	16	60.04
**116**	*Mycobacterium massiliense* 2B-0912-S [d]	Sputum, Human	NZ_AKUW00000000	2	N.A.	972582-1005868	33.2	Q	58	59.76
						2681757-2711731	29.9	N	16	59.65
**117**	*Mycobacterium massiliense* 2B-1231 [d]	Bronchial alveolar lavage, Human	NZ_AKUO00000000	1	N.A.	1034844-1068130	33.2	Q	59	59.76
**118**	*Mycobacterium massiliense* CCUG 48898 [d g]	Human sputum and bronchial alveolar lavage, Human, France	NZ_AHAR00000000	3	N.A.	1793291-1822891	29.6	N	29	60.35
						3907391-3945321	37.9	Q	49	63.98
						3945337-3957588	12.2	N	17	64.75
**119**	*Mycobacterium massiliense* CCUG 48898 [d g]	Human sputum and bronchial alveolar lavage, Human, France	NZ_AKVF00000000	3	N.A.	1600973-1653490	52.5	Q	78	59.72
						3907330-3945260	37.9	Q	51	63.98
						3945276-3957680	12.4	N	17	64.69
**120**	*Mycobacterium massiliense* M18 [d]	Lymph node biopsy of a Malaysian patient with suspected tuberculosis cervical lymphadenitis, Human, Malaysia	NZ_AJSC00000000	2	N.A.	4798547-4830969	32.4	N	49	64.36
						4824727-4842815	18	N	18	64.47
**121**	*Mycobacterium massiliense* str. GO 06	N.D.	NC_018150	0	0.00 %					
**122**	*Mycobacterium neoaurum* VKM Ac-1815D	N.D.	NC_023036	1	2.49 %	4072080-4207674	135.5	Y	127	67.22
**123**	*Mycobacterium parascrofulaceum* ATCC BAA-614 [d]	Clinical specimen (human), Canada	NZ_ADNV00000000	3	N.A.	52-11606	11.5	N	10	59.24
						4336682-4371755	35	Q	23	63.22
						4631977-4695036	63	Y	73	66.63
**124**	*Mycobacterium phlei* RIVM601174 [d]	Human, Netherlands	NZ_AJFJ00000000	2	N.A.	103112-135455	32.3	N	22	68.77
						147443-156919	9.4	N	10	66.96
**125**	*Mycobacterium* sp. 155 [d]	N.D.	NZ_AREU00000000	2	N.A.	1260533-1279296	18.7	N	25	66.39
						3097490-3104486	6.9	N	9	64.68
**126**	*Mycobacterium* sp. H4Y [d]	Sputum, Human, South Korea	NZ_AKIG00000000	4	N.A.	1379632-1404004	24.3	N	10	65.37
						2401904-2412571	10.6	N	11	63.45
						2876718-2894347	17.6	N	16	61.72
						3849582-3885710	36.1	Q	19	64.49

Sequence number	Organism	Isolation source [c]	Accession/GI number	No. putative prophage region(s)	Percentage of genome encoding putative prophage region(s)[e]	Coordinates within host genome [a]	Size (kb)	Intact [b]	No. CDS	G+C mol % of prophage region
**127**	*Mycobacterium* sp. JLS	Creosote-contaminated soil, United States	NC_009077	0	0.00 %					
**128**	*Mycobacterium* sp. KMS [f]	N.D.	NC_008705	2	0.81 %	3074173-3108448	34.2	N	19	67.84
						4085194-4097492	12.2	N	21	67.44
**129**	*Mycobacterium* sp. MCS [f]	N.D.	NC_008146	3	1.53 %	1770462-1810940	40.4	N	21	63.50
						3056285-3090560	34.2	N	19	67.84
						4050658-4063010	12.3	N	20	67.35
**130**	*Mycobacterium* sp. MOTT36Y	Sputa, Human, South Korea	NC_017904	2	0.36 %	967361-980458	13	N	9	65.15
						4758520-4765550	7	N	9	65.50
**131**	*Mycobacterium rhodesiae* JS60 [d]	Soil	NZ_AGIQ00000000	7	N.A.	1446937-1458089	11.1	N	13	59.52
						2005316-2016262	10.9	N	12	65.84
						3030284-3060900	30.6	Q	21	66.71
						5994993-6024881	29.8	N	66	60.49
						6371973-6397968	25.9	N	19	67.07
						6418923-6448661	29.7	Y	21	61.38
						7156405-7191386	34.9	Y	28	63.27
**132**	*Mycobacterium rhodesiae* NBB3	Estuarine sediment, Australia	NC_016604	0	0.00 %					
**133**	*Mycobacterium smegmatis* JS623 [f]	Smegma, Human	NC_019966	1	0.40 %	4206686-4232683	25.9	N	14	64.24
**134**	*Mycobacterium smegmatis* str. MC2 155 [g]	N.D.	NC_008596	2	0.28 %	1942190-1952852	10.6	N	10	64.44
						3499988-3508558	8.5	N	9	64.72
**135**	Mycobacterium smegmatis str. MC2 155 [g]	N.D.	NC_018289	1	0.28 %	1941442-1960865	19.4	N	13	65.58
**136**	*Mycobacterium smegmatis* MKD8 [d]	Japan	NZ_AOCJ00000000	3	N.A.	1334549-1341067	6.5	N	7	67.11
						2254937-2278040	23.1	Y	23	65.25
						5526039-5539167	13.1	N	9	64.04
**137**	*Mycobacterium thermoresistibile* ATCC 19527 [d]	Soil	NZ_AGVE00000000	2	N.A.	1438114-1460818	22.7	N	9	66.27
						1690775-1760309	69.5	Q	77	67.11
**138**	*Mycobacterium tuberculosis* 02_1987 [d]	N.D.	NZ_ABLM00000000	2	N.A.	762700-770225	7.5	N	12	65.17
						2582785-2610715	27.9	N	14	66.01
**139**	*Mycobacterium tuberculosis* 7199-99	N.D.	NC_020089	3	1.10 %	1162662-1170198	7.5	N	10	65.25
						2970659-2983113	12.4	Q	19	66.27
						3881705-3910421	28.7	N	22	65.18
**140**	*Mycobacterium tuberculosis* 94_M4241A [d]	N.D.	NZ_ABLL00000000	1	N.A.	2706498-2715777	9.2	N	13	66.38
**141**	*Mycobacterium tuberculosis* '98-R604 INH-RIF-EM' [d]	N.D.	NZ_ABVM00000000	3	N.A.	1242115-1249638	7.5	N	13	65.26
						2070321-2083497	13.1	N	13	64.38
						2993899-3010376	16.4	N	14	65.78
**142**	*Mycobacterium tuberculosis* str. Beijing/NITR203	N.D.	NC_021054	2	0.80 %	1766846-1788161	21.3	N	17	66.14
						2969790-2983602	13.8	Q	19	66.05
**143**	*Mycobacterium tuberculosis* C [d]	N.D.	NZ_AAKR00000000	2	N.A.	796046-803577	7.5	N	13	65.16
						2559632-2569050	9.4	N	13	66.89
**144**	*Mycobacterium tuberculosis* CAS/NITR204	N.D.	NC_021193	3	1.18 %	1154502-1162028	7.5	N	12	65.25
						1771759-1790415	18.6	N	18	66.62
						2958132-2983883	25.7	N	19	66.24
**145**	*Mycobacterium tuberculosis* CCDC5079 [g]	N.D.	NC_017523	2	0.39 %	1154189-1161716	7.5	N	8	65.24
						2951861-2961273	9.4	N	9	66.94
**146**	*Mycobacterium tuberculosis* CCDC5079 [g]	N.D.	NC_021251	2	0.45 %	1156530-1164066	7.5	N	13	65.24
						2964597-2977050	12.4	Q	18	66.28
**147**	*Mycobacterium tuberculosis* CCDC5180	Sputum from patient with secondary pulmonary tuberculosis, Human,	NC_017522	2	0.38 %	1154121-1161648	7.5	N	8	65.24
						2958267-2967679	9.4	N	9	66.93

Sequence number	Organism	Isolation source [c]	Accession/GI number	No. putative prophage region(s)	Percentage of genome encoding putative prophage region(s)[e]	Coordinates within host genome [a]	Size (kb)	Intact [b]	No. CDS	G+C mol % of prophage region
		China								
**148**	*Mycobacterium tuberculosis* CDC1551	N.D.	NC_002755	2	1.18 %	677260-703462	26.2	N	16	64.95
						3870574-3896495	25.9	N	20	65.17
**149**	*Mycobacterium tuberculosis* CDC1551A [d]	N.D.	NZ_AELF00000000	4	N.A.	805760-813180	7.4	N	12	64.17
						1650077-1670154	20	N	17	64.38
						2592402-2613751	21.3	Q	15	66.07
						3490212-3518761	28.5	N	19	65.12
**150**	*Mycobacterium tuberculosis* CPHL_A [d]	N.D.	NZ_ACHP00000000	2	N.A.	1618176-1625703	7.5	N	13	65.25
						3418304-3427720	9.4	N	14	66.88
**151**	*Mycobacterium tuberculosis* CTRI-2	Russia	NC_017524	2	0.45 %	1158389-1165925	7.5	N	10	65.24
						2964965-2977418	12.4	Q	19	66.26
**152**	*Mycobacterium tuberculosis* CTRI-4 [d]	Sputum, Human, Russia	NZ_AIIE00000000	2	N.A.	984864-992391	7.5	N	12	65.25
						2772473-2781885	9.4	N	13	66.93
**153**	*Mycobacterium tuberculosis* EAI5/NITR206	N.D.	NC_021194	3	0.94 %	1156406-1163939	7.5	N	13	65.22
						1763970-1785203	21.2	N	11	66.13
						2957342-2969796	12.4	Q	17	66.24
**154**	*Mycobacterium tuberculosis* EAI5 MUM101	N.D.	NC_021740	1	0.17 %	1156610-1164137	7.5	N	12	65.25
**155**	*Mycobacterium tuberculosis* EAS054 [d]	N.D.	NZ_ABOV00000000	2	N.A.	813712-821239	7.5	N	13	65.22
						1415179-1435797	20.6	N	12	66.11
**156**	*Mycobacterium tuberculosis* str. Erdman = ATCC 35801	Human sputum	NC_020559	3	1.42 %	1157016-1164543	7.5	N	13	65.25
						2945745-2970758	25	Q	18	66.28
						3866067-3895979	29.9	N	21	65.16
**157**	*Mycobacterium tuberculosis* F11	N.D.	NC_009565	3	0.67 %	1161731-1169267	7.5	N	10	65.28
						1989026-1998675	9.6	N	6	67.59
						2983380-2995833	12.4	Q	18	66.26
**158**	*Mycobacterium tuberculosis* GM 1503 [d]	N.D.	NZ_ABQG00000000	1	N.A.	2559244-2580461	21.2	N	13	65.68
**159**	*Mycobacterium tuberculosis* H37Ra (ATCC 25177) [d g]	N.D.	NZ_AAYK00000000	1	N.A.	348817-357557	8.7	N	12	66.03
**160**	*Mycobacterium tuberculosis* H37Ra (ATCC 25177) [g]	N.D.	NC_009525	3	0.97 %	1159271-1166807	7.5	N	11	65.25
						1768504-1790023	21.5	N	21	66.10
						2982031-2995842	13.8	Q	21	66.05
**161**	*Mycobacterium tuberculosis* H37RvCO [d]	N.D.	NZ_AJSF00000000	2	N.A.	1739822-1760951	21.1	N	11	66.07
						2935352-2946046	10.6	Q	16	66.46
**162**	*Mycobacterium tuberculosis* H37Rv (ATCC 27294) [g]	Lung, Human	NC_000962	3	0.97 %	1157963-1165499	7.5	N	10	65.25
						1766987-1788505	21.5	N	22	66.10
						2970063-2983874	13.8	Q	21	66.05
**163**	*Mycobacterium tuberculosis* H37Rv (ATCC 27294) [g]	Lung, Human	NC_018143	3	1.33 %	675812-702017	26.2	N	19	64.96
						1766993-1788512	21.5	N	22	66.10
						2970073-2980843	10.7	Q	14	66.57
**164**	*Mycobacterium tuberculosis* str. Haarlem [g]	N.D.	NC_022350	3	1.10 %	1162925-1170461	7.5	N	11	65.25
						2965323-2977777	12.4	Q	19	66.28
						3870603-3899317	28.7	N	25	65.17
**165**	*Mycobacterium tuberculosis* str. Haarlem [d g]	N.D.	NZ_AASN00000000	2	N.A.	2457761-2479074	21.3	N	14	65.74
						3366165-3387877	21.7	N	22	65.22
**166**	*Mycobacterium tuberculosis* str. Haarlem/NITR202	N.D.	NC_021192	2	0.42 %	1160580-1168142	7.5	N	12	64.62
						1780017-1790814	10.7	N	11	66.48
**167**	*Mycobacterium tuberculosis* K85 [d]	N.D.	NZ_ACHQ00000000	2	N.A.	1628710-1636237	7.5	N	12	65.22
						3263433-3278255	14.8	N	16	70.76
**168**	*Mycobacterium tuberculosis* KZN 605	Human, South Africa	NC_018078	2	0.63 %	1431411-1443844	12.4	Q	19	66.28

Sequence number	Organism	Isolation source [c]	Accession/GI number	No. putative prophage region(s)	Percentage of genome encoding putative prophage region(s)[e]	Coordinates within host genome [a]	Size (kb)	Intact [b]	No. CDS	G+C mol % of prophage region
						2369462-2384656	15.1	N	12	65.07
**169**	*Mycobacterium tuberculosis* KZN 1435	N.D.	NC_012943	3	0.80 %	1431299-1443732	12.4	Q	19	66.28
						2369580-2384774	15.1	N	13	65.07
						3246469-3254005	7.5	N	11	65.25
**170**	*Mycobacterium tuberculosis* KZN 4207 [g]	Human, South Africa	NC_016768	3	0.80 %	1431243-1443676	12.4	Q	19	66.28
						2366311-2381505	15.1	N	13	65.07
						3243202-3250738	7.5	N	10	65.25
**171**	*Mycobacterium tuberculosis* KZN 4207 [d g]	South Africa	NZ_ACVS00000000	2	N.A.	1155088-1162615	7.5	N	12	65.25
						2021092-2037144	16	N	15	65.08
**172**	*Mycobacterium tuberculosis* KZN R506 [d]	South Africa	NZ_ACVU00000000	3	N.A.	1151605-1159132	7.5	N	12	65.25
						2013900-2029952	16	N	17	65.08
						2949867-2959279	9.4	N	14	66.92
**173**	*Mycobacterium tuberculosis* KZN V2475 [d]	South Africa	NZ_ACVT00000000	3	N.A.	1147742-1155269	7.5	N	12	65.25
						2008496-2024548	16	N	17	65.08
						2943451-2952863	9.4	N	13	66.92
**174**	*Mycobacterium tuberculosis* NA-A0008 [d]	Sputum from a patient infected with tuberculosis, Human, India	NZ_ALYG00000000	4	N.A.	1116822-1131718	14.8	N	15	64.02
						1465364-1480117	14.7	Q	17	65.38
						1956890-1967034	10.1	N	12	66.25
						4111401-4122982	11.5	N	16	66.48
**174**	*Mycobacterium tuberculosis* NA-A0009 [d]	Sputum from a patient infected with tuberculosis, Human, India	NZ_ALYH00000000	3	N.A.	2605534-2614781	9.2	N	11	65.93
						2722301-2727905	5.6	N	7	66.49
						4127334-4137011	9.6	N	11	65.05
**175**	*Mycobacterium tuberculosis* NCGM2209 [d]	N.D.	NZ_BADQ00000000	2	N.A.	3299583-3308800	9.2	N	13	66.62
						4252824-4279687	26.8	Q	42	69.28
**176**	*Mycobacterium tuberculosis* OSDD071 [d]	Sputum, Human, India	NZ_AHHX00000000	5	N.A.	3546002-3559700	13.6	N	12	66.33
						3967164-3981125	13.9	N	15	63.84
						4012377-4029249	16.8	N	22	65.53
						4185952-4213724	27.7	N	15	65.32
						4261104-4277115	16	N	22	71.26
**177**	*Mycobacterium tuberculosis* OSDD504 [d]	Sputum, Human, India	NZ_AHHY00000000	2	N.A.	4103956-4135263	31.3	N	23	66.32
						4207025-4225760	18.7	N	30	71.14
**178**	*Mycobacterium tuberculosis* OSDD518 [d]	Sputum, Human, India	NZ_AHHZ00000000	4	N.A.	778923-790183	11.2	N	11	64.29
						2189485-2215521	26	N	13	64.96
						4173009-4186875	13.8	N	20	66.23
						4225711-4237934	12.2	N	14	70.98
**179**	*Mycobacterium tuberculosis* PR05 [d]	Cerebrospinal fluid sample from tuberculosis patient, Human, Malaysia	NZ_AOMG00000000	2	N.A.	4220539-4237804	17.2	N	19	67.79
						4237971-4249673	11.7	N	16	67.76
**180**	*Mycobacterium tuberculosis* RGTB327	N.D.	NC_017026	1	0.52 %	2955788-2978471	22.6	Q	19	65.66
**181**	*Mycobacterium tuberculosis* RGTB423	N.D.	NC_017528	3	1.17 %	1158796-1166333	7.5	N	12	65.23
						1766223-1787559	21.3	N	16	66.10
						2968585-2991092	22.5	Q	8	66.39
**182**	*Mycobacterium tuberculosis* SP21 [d]	Sputum, Human, Russia	NZ_AOUF00000000	2	N.A.	3028440-3047275	18.8	N	19	66.33
						4226328-4247483	21.1	N	36	67.68
**183**	*Mycobacterium tuberculosis* SUMu001 [d]	N.D.	NZ_ADHQ00000000	2	N.A.	1636588-1658107	21.5	N	10	66.10
						2828905-2858037	29.1	Q	16	66.22
**184**	*Mycobacterium tuberculosis* SUMu002 [d]	N.D.	NZ_ADHR00000000	3	N.A.	2565992-2590251	24.2	Q	16	65.99
						3626157-3633628	7.4	N	10	65.24
						4266416-4292823	26.4	N	13	65.32

Sequence number	Organism	Isolation source [c]	Accession/GI number	No. putative prophage region(s)	Percentage of genome encoding putative prophage region(s)[e]	Coordinates within host genome [a]	Size (kb)	Intact [b]	No. CDS	G+C mol % of prophage region
**185**	*Mycobacterium tuberculosis* SUMu003 [d]	N.D.	NZ_ADHS00000000	2	N.A.	1145200-1152919	7.7	N	12	65.17
						2906967-2930905	23.9	Q	17	65.98
**186**	*Mycobacterium tuberculosis* SUMu004 [d]	N.D.	NZ_ADHT00000000	2	N.A.	1287368-1298073	10.7	Q	14	64.82
						3312974-3336637	23.6	Q	14	65.68
**187**	*Mycobacterium tuberculosis* SUMu005 [d]	N.D.	NZ_ADHU00000000	2	N.A.	1407311-1417951	10.6	Q	14	64.81
						3181196-3190753	9.5	N	11	66.53
**188**	*Mycobacterium tuberculosis* SUMu006 [d]	N.D.	NZ_ADHV00000000	2	N.A.	1578121-1585439	7.3	N	10	65.10
						3360456-3379477	19	N	16	66.03
**189**	*Mycobacterium tuberculosis* SUMu007 [d]	N.D.	NZ_ADHW00000000	4	N.A.	1597704-1605570	7.8	N	10	65.08
						2231826-2241238	9.4	N	12	66.89
						3550134-3563489	13.1	N	16	67.63
						4265456-4291852	26.3	N	16	65.29
**190**	*Mycobacterium tuberculosis* SUMu008 [d]	N.D.	NZ_ADHX00000000	3	N.A.	1616154-1623602	7.4	N	10	65.19
						3375186-3395319	20.1	N	22	66.25
						4270109-4296538	26.4	N	17	65.13
**191**	*Mycobacterium tuberculosis* SUMu009 [d]	N.D.	NZ_ADHY00000000	3	N.A.	805722-813334	7.6	N	11	65.03
						2453948-2474856	20.9	N	19	66.04
						3357622-3383547	25.9	N	14	65.22
**192**	*Mycobacterium tuberculosis* SUMu010 [d]	N.D.	NZ_ADHZ00000000	3	N.A.	804355-811998	7.6	N	11	65.15
						1403436-1424954	21.5	N	10	66.10
						2603946-2617822	13.8	Q	20	65.94
**193**	*Mycobacterium tuberculosis* SUMu011 [d]	N.D.	NZ_ADIA00000000	3	N.A.	805458-812801	7.3	N	10	65.18
						2593480-2613469	19.9	Q	17	65.87
						3347480-3360445	12.9	N	13	62.72
**194**	*Mycobacterium tuberculosis* SUMu012 [d]	N.D.	NZ_ADIB00000000	1	N.A.	2888632-2912934	24.3	Q	16	65.93
**195**	*Mycobacterium tuberculosis* T17 [d]	N.D.	NZ_ABQH00000000	3	N.A.	1734479-1757146	22.6	Q	15	66.05
						1998486-2019930	21.4	N	19	64.39
						2921915-2937415	15.5	N	15	64.89
**196**	*Mycobacterium tuberculosis* T46 [d]	N.D.	NZ_ACHO00000000	3	N.A.	1586190-1597798	11.6	N	15	65.41
						2189548-2211048	21.5	N	11	66.12
						2451905-2481538	29.6	Q	28	64.41
**197**	*Mycobacterium tuberculosis* T85 [d]	N.D.	NZ_ABOW00000000	2	N.A.	802334-809861	7.5	N	13	65.24
						2567273-2587102	19.8	N	14	65.71
**198**	*Mycobacterium tuberculosis* T92 [d]	N.D.	NZ_ABLN00000000	3	N.A.	1479054-1486581	7.5	N	12	65.22
						2315352-2344204	28.8	N	27	64.57
						3228210-3253026	24.8	N	14	65.55
**199**	*Mycobacterium tuberculosis* UM 1072388579 [d]	Sputum, Human, Malaysia	NZ_AMXW00000000	0	N.A.					
**200**	*Mycobacterium tuberculosis* W-148 [d]	N.D.	NZ_ACSX00000000	3	N.A.	2006793-2014239	7.4	N	7	63.15
						2341000-2350413	9.4	N	12	66.93
						2850476-2858003	7.5	N	13	65.22
**201**	*Mycobacterium tuberculosis* UT205	N.D.	NC_016934	0	0.00 %					
**202**	*Mycobacterium tusciae* JS617 [d]	Granular activated carbon, Germany	NZ_AGJJ00000000	7	N.A.	2439862-2460800	20.9	N	14	66.41
						2614214-2627979	13.7	N	8	68.10
						2652769-2661271	8.5	N	10	67.36
						2671348-2691458	20.1	N	21	68.01
						3302641-3323558	20.9	N	19	66.84
						5600545-5660573	60	N	59	66.26
						7083980-7115419	31.4	Y	47	63.38

Sequence number	Organism	Isolation source [c]	Accession/GI number	No. putative prophage region(s)	Percentage of genome encoding putative prophage region(s)[e]	Coordinates within host genome [a]	Size (kb)	Intact [b]	No. CDS	G+C mol % of prophage region
**203**	*Mycobacterium ulcerans* Agy99 [f]	Clinical sample, Human, Ghana	NC_008611	2	0.37 %	917436-929425	11.9	N	11	67.20
						3755296-3764098	8.8	N	10	64.97
**204**	*Mycobacterium vaccae* ATCC 25954 [d]	Human	NZ_ALQA00000000	1	N.A.	5506007-5526152	20.1	N	18	67.87
**205**	*Mycobacterium vanbaalenii* PYR-1	Estuarine sediments polluted with petrol, United States	NC_008726	1		538064-548679	10.6	N	14	66.15
**206**	*Mycobacterium xenopi* RIVM700367 [d]	Human, Netherlands	NZ_AJFI00000000	3	N.A.	58832-68963	10.1	Q	10	64.34
						837197-847850	10.6	Y	11	64.23
						2447053-2492648	45.5	Y	50	65.63
**207**	*Mycobacterium yongonense* 05-1390 [f]	Pulmonary patient, male, 64 years old, Human, South Korea	NC_021715	3	0.89 %	3542832-3554525	11.6	N	10	65.35
						3643049-3660989	17.9	N	15	62.15
						4993761-5013489	19.7	N	12	64.80
**208**	*Nocardia abscessus* NBRC 100374 [d]	Joint abscess of a 56 year old man with a complete endoprosthesis of one of his knees, Germany	NZ_BAFP00000000	2	N.A.	7411413-7432989	21.5	N	35	66.14
						7441249-7466590	25.3	Y	29	66.14
**209**	*Nocardia aobensis* NBRC 100429 [d f]	Human, Japan	NZ_BAFQ00000000	1	N.A.	5984929-5998119	13.1	Q	12	65.60
**210**	*Nocardia araoensis* NBRC 100135 [d]	Human, Japan	NZ_BAFR00000000	2	N.A.	4491835-4508037	16.2	N	10	65.64
						6778720-6806191	27.4	Y	16	65.44
**211**	*Nocardia asiatica* NBRC 100129 [d]	Human, Japan	NZ_BAFS00000000	2	N.A.	6230003-6239024	9	N	15	66.80
					N.A.	7553518-7567749	14.2	N	19	65.74
**212**	*Nocardia asteroides* NBRC 15531 [d]	N.D.	NZ_BAFO00000000	1	N.A.	6464139-6483706	19.5	N	12	68.21
**213**	*Nocardia brasiliensis* ATCC 700358	Human mycetoma, Mexico	NC_018681	0	0.00 %					
**214**	*Nocardia brasiliensis* NBRC 14402 [d]	N.D.	NZ_BAFT00000000	0	N.A.					
**215**	*Nocardia brevicatena* NBRC 12119 [d]	Sputa	NZ_BAFU00000000	2	N.A.	184108-194293	10.1	N	14	66.24
						5608530-5639170	30.6	Y	36	65.31
**216**	*Nocardia carnea* NBRC 14403 [d]	N.D.	NZ_BAFV00000000	0	N.A.					
**217**	*Nocardia cerradoensis* NBRC 101014 [d]	Soil, Brazil	NZ_BAFW00000000	0	N.A.					
**218**	*Nocardia concava* NBRC 100430 [d]	Human, Japan	NZ_BAFX00000000	3	N.A.	4237606-4247437	9.8	N	7	67.21
						7713017-7726248	13.2	Q	16	66.54
						7715411-7742576	27.1	N	26	66.73
**219**	*Nocardia cyriacigeorgica* GUH-2	N.D.	NC_016887	0	0.00 %					
**220**	*Nocardia cyriacigeorgica* NBRC 100375 [d]	Bronchial secretions, Germany	NZ_BAFY00000000	0	N.A.					
**221**	*Nocardia exalbida* NBRC 100660 [d]	A bronchoalveolar lavage of a 43-year-old immunocompromised patient with a lung abscess, China	NZ_BAFZ00000000	1	N.A.	6520306-6528930	8.6	N	9	67.08
**222**	*Nocardia farcinica* IFM 10152 [f]	N.D.	NC_006361	4	1.93 %	28359-50108	21.7	Y	24	69.93
						1669018-1707866	38.8	Y	54	67.47
						4103113-4143292	40.1	Y	41	65.33
						4136309-4151800	15.4	N	24	64.19
**223**	*Nocardia higoensis* NBRC 100133 [d]	Human, Japan	NZ_BAGA00000000	0	N.A.					
**224**	*Nocardia jiangxiensis* NBRC 101359 [d]	Rhizosphere soil (about pH 3.5) of goose-grass (*Elusine indica*) growing next to a copper mine, China	NZ_BAGB00000000	2	N.A.	7163124-7176923	13.8	N	30	58.91
						7209365-7238755	29.3	Q	33	59.68
**225**	*Nocardia niigatensis* NBRC 100131 [d]	Human, Japan	NZ_BAGC00000000	1	N.A.	236676-267140	30.4	Y	36	66.87
**226**	*Nocardia otitidiscaviarum* NBRC 14405 [d]	Ear of guinea pig	NZ_BAGD00000000	1	N.A.	6425537-6434991	9.4	N	17	66.38
**227**	*Nocardia paucivorans* NBRC 100373 [d]	Sputum, Germany	NZ_BAGE00000000	1	N.A.	2423284-2437578	14.2	N	16	64.68
**228**	*Nocardia pneumoniae* NBRC 100136 [d]	Human, Japan	NZ_BAGF00000000	0	N.A.					
**229**	*Nocardia takedensis* NBRC 100417 [d]	Moat sediment, Japan	NZ_BAGG00000000	3	N.A.	5767857-5774953	7.0	N	8	70.10
						5894626-5916891	22.2	Y	28	67.16
						6181247-6203580	22.3	N	6	67.53

Sequence number	Organism	Isolation source [c]	Accession/GI number	No. putative prophage region(s)	Percentage of genome encoding putative prophage region(s)[e]	Coordinates within host genome [a]	Size (kb)	Intact [b]	No. CDS	G+C mol % of prophage region
**230**	*Nocardia tenerifensis* NBRC 101015 [d]	Soil, Canary Islands	NZ_BAGH00000000	1	N.A.	8418932-8432932	14.0	Y	20	63.04
**231**	*Nocardia testacea* NBRC 100365 [d]	Human, Japan	NZ_BAGJ00000000	1	N.A.	1435697-1457180	21.4	Q	21	67.25
**232**	*Nocardia thailandica* NBRC 100428 [d]	Human, Thailand	NZ_BAGK00000000	4	N.A.	1008436-1069196	60.7	Q	68	70.64
						3593195-3613718	20.5	Q	17	66.98
						3612039-3624898	12.8	N	19	67.93
						3628939-3643106	14.1	N	28	67.04
**233**	*Nocardia veterana* NBRC 100344 [d]	Bronchioscopic lavage, Australia	NZ_BAGM00000000	0	N.A.					
**234**	*Nocardia vinacea* NBRC 16497 [d]	Soil, Japan	NZ_BAGN00000000	0	N.A.					
**235**	*Rhodococcus equi* 103S	*Equus caballus*, Canada	NC_014659	0	0.00 %					
**236**	*Rhodococcus equi* ATCC 33707 [d]	Human abscess and/or skin	NZ_ADNW00000000	2	N.A.	1795183-1844172	48.9	Y	57	66.93
						2174691-2219151	44.4	Y	72	65.57
**237**	*Rhodococcus erythropolis* CCM2595 (ATCC 11048) [f]	Soil	NC_022115	1	0.18 %	4660425-4671424	11	N	8	59.09
**238**	*Rhodococcus erythropolis* PR4 [f]	Seawater, Pacific Ocean	NC_012490	3	0.88 %	2445447-2470489	25	Q	30	61.96
						4351039-4373917	22.8	N	9	61.35
						4936069-4945592	9.5	N	7	58.63
**239**	*Rhodococcus erythropolis* SK121 [d]	N.D.	NZ_ACNO00000000	2	N.A.	3024554-3041304	16.7	N	7	61.01
						6558023-6563559	5.5	N	7	61.13
**240**	*Rhodococcus imtechensis* RKJ300 [d]	Soil, India	NZ_AJJH00000000	3	N.A.	280337-303673	23.3	Q	10	65.51
						5386240-5405206	18.9	N	14	64.01
						5775690-5782910	7.2	N	9	66.72
**241**	*Rhodococcus jostii* RHA1 [f]	N.D.	NC_008268	1	0.22 %	7055617-7072621	17	N	12	65.65
**242**	*Rhodococcus opacus* B4 [f]	N.D.	NC_012522	3	0.76 %	320326-329656	9.3	N	11	67.59
						4321619-4339482	17.8	N	28	63.64
						4335275-4368502	33.2	Y	31	63.63
**243**	*Rhodococcus opacus* M213 [d]	Fuel-oil contaminated soil, United States	NZ_AJYC00000000	1	N.A.	1919282-1961835	42.5	Y	57	65.47
**244**	*Rhodococcus opacus* PD630 [d]	N.D.	NZ_AGVD00000000	0	N.A.					
**245**	*Rhodococcus pyridinivorans* AK37 [d]	N.D.	NZ_AHBW00000000	2	N.A.	294536-327014	32.4	Y	48	66.44
						4926772-4948625	21.8	Q	21	67.82
**246**	*Rhodococcus pyridinivorans* SB3094 [f]	N.D.	NC_023150	0	0.00 %					
**247**	*Rhodococcus qingshengii* BKS 20-40 [d]	Soil, India	NZ_AODN00000000	0	N.A.					
**248**	*Rhodococcus rhodochrous* BKS6-46 [d]	Mangrove forest soil sample, India	NZ_AGVW00000000	3	N.A.	124130-146400	22.2	N	27	64.82
						216568-231833	15.2	N	17	69.28
						4675363-4693294	17.9	N	23	66.96
**249**	*Rhodococcus ruber* BKS 20-38 [d]	Soil, India	NZ_AOEX00000000	4	N.A.	393201-401238	8	N	8	70.09
						2225972-2254658	28.6	N	15	72.00
						3534043-3539655	5.6	N	6	66.29
						5184902-5231057	46.1	Q	44	66.59
**250**	*Rhodococcus ruber* Chol-4 [d]	Sewage sludge, Spain	NZ_ANGC00000000	0	N.A.					
**251**	*Rhodococcus* sp. AW25M09 [d]	N.D.	NZ_CAPS00000000	0	N.A.					
**252**	*Rhodococcus* sp. DK17 [d]	Crude oil contaminated soil, Korea	NZ_AJLQ00000000	0	N.A.					
**253**	*Rhodococcus* sp. JVH1 [d]	Canada	NZ_AKKP00000000	1	N.A.	1529730-1569966	40.2	N	46	64.74
**254**	*Rhodococcus* sp. P14 [d]	Crude oil contaminated sediments, China	NZ_AJFC00000000	0	N.A.					
**256**	*Rhodococcus* sp. R1101 [d]	Cavitary lung lesion, Human, United States	NZ_AJVB00000000	0	N.A.					
**257**	*Rhodococcus triatomae* BKS 15-14 [d]	Soil, India	NZ_AODO00000000	1	N.A.	5554117-5592017	37.9	Y	38	64.55
**258**	*Rhodococcus wratislaviensis* IFP 2016 [d]	France	NZ_ANIU00000000	2	N.A.	2724077-2749023	24.9	N	11	64.55

Sequence number	Organism	Isolation source [c]	Accession/GI number	No. putative prophage region(s)	Percentage of genome encoding putative prophage region(s)[e]	Coordinates within host genome [a]	Size (kb)	Intact [b]	No. CDS	G+C mol % of prophage region
						4251757-4264455	12.6	N	10	67.19
**259**	*Tsukamurella paurometabola* DSM 20162 [f]	N.D.	NC_014158	1	0.49 %	1490234-1511515	21.2	Q	21	65.13

[a] Coordinates for draft sequences are based on concatenation of all contigs by PHAST
[b] Y indicates Yes (putative complete predicted prophage), N indicates No (putative incomplete predicted prophage), Q indicates Questionable (completeness of putative predicted prophage is questionable)
[c] Isolation data obtained from GenBank and ATCC, DSMZ, CCUG, NTCC culture collection websites
[d] Draft genome sequence in multiple contigs
[e] Calculated where bacterial genome sequences appear complete
[f] Corresponding plasmids' screened (see Table S2)
[g] Multiple assemblies screened and listed individually
N.D. indicates no data available
N.A indicates not applicable
