# Supplementary material for: Locating and Activating Molecular ‘Time Bombs’: Induction of Mycolata Prophages
Source: PLoS One. 2016 Aug 3;11(8):e0159957. doi: 10.1371/journal.pone.0159957 (PMC4972346; doi:10.1371/journal.pone.0159957)
Supplement: S2 Table — (PDF) [file pone.0159957.s002.pdf]

S2 Table: Prophages detected in plasmid sequences using PHAST

Plasmid(s) screened using PHAST					PHAST predicted prophage(s)					
**Source organism**	**Plasmid**	**Accession number**	**Plasmid size (bp)**	**Genome structure**	**No. prophage regions**	**Intact[a]**	**Coordinates[b]**	**Size (kb)**	**No. CDS**	**G+C content (% mol)**
***Gordonia bronchialis* DSM 43247**	pGBRO01	NC_013442	81410	Circular	0					
***Gordonia* sp. KTR9**	pGKT2	NC_018580	182454	Circular	0					
	pGKT1	NC_018582	89480	Circular	0					
	pGKT3	NC_018583	172385	Circular	1	Y	98742-134788		14	61.13
***Gordonia polyisoprenivorans* VH2**	p174	NC_016907	174494	Circular	0					
***Gordonia westfalica* strain DSM44215T**	pKB1	NC_005307	101016	Circular	0					
***Mycobacterium abscessus* subsp. bolletii 50594**	Plasmid 1	NC_021278	172814	Circular	0					
	Plasmid 2	NC_021279	97240	Circular	0					
***Mycobacterium* abscessus subsp. bolletii CRM-0020**	plasmid unnamed	NZ_ATFQ01000044	56466	Linear	0					
***Mycobacterium abscessus* subsp. bolletii F1725**	BRA100	NC_017908	56265	Circular	0					
***Mycobacterium abscessus* ATCC 19977**	Unnamed plasmid	NC_010394	23319	Circular	0					
Mycobacterium avium	pVT2	NC_005016	12868	Circular	0					
Mycobacterium celatum	pCLP	NC_004963	22688	Linear	0					
***Mycobacterium chubuense* NBB4**	pMYCCH.02	NC_018023	143623	Circular	0					
	pMYCCH.01	NC_018022	615278	Circular	1	N	444996-456797	11.8	10	65.30
Mycobacterium fortuitum	pAL5000	NC_001381	4837	Circular	0					
***Mycobacterium gilvum* PYR-GCK**	pMFLV01	NC_009339	321253	Linear	1	Q	164351-215973	51.6	23	65.37
	pMFLV02	NC_009340	25309	Circular	0					
	pMFLV03	NC_009341	16660	Circular	0					
***Mycobacterium gilvum* Spyr1**	pMSPYR101	NC_014811	211864	Circular	0					
	pMSPYR102	NC_014812	23681	Circular	0					
***Mycobacterium kansasii* ATCC 12478**	pMK12478	NC_022654	144951	Circular	0					
***Mycobacterium* sp. KMS**	pMKMS02	NC_008704	216763	Circular	1	Y	174491-216428	41.9	28	65.03
	pMKMS01	NC_008703	302089	Circular	0					
***Mycobacterium liflandii* 128FXT**	pMUM002	NC_011355	190588	Circular	0					
***Mycobacterium marinum* DL240490**	pMUM003	NC_019018	104530	Linear	0					
***Mycobacterium marinum* M**	pMM23	NC_010604	23317	Circular	0					
***Mycobacterium* sp. MCS**	Plasmid 1	NC_008147	215075	Linear	1	Y	149207-183300	34	35	63.86
***Mycobacterium smegmatis* JS623**	pMYCSM01	NC_019957	394147	Circular	0					
	pMYCSM02	NC_019958	198589	Circular	0					
	pMYCSM03	NC_019959	164114	Circular	0					
***Mycobacterium ulcerans* AGY99**	pMUM001	NC_005916	174155	Circular	1	Y	138963-160330	21.3	24	61.36
***Mycobacterium yongonense* 05-1390**	pMyong1	NC_020275	122976	Circular	0					
	pMyong2	NC_020276	18089	Circular	0					
Nocardia aobensis	pYS1	NC_013448	4326	Circular	0					
***Nocardia farcinica* IFM 10152**	pNF1	NC_006362	184026	Circular	0					

Plasmid(s) screened using PHAST					PHAST predicted prophage(s)					
	pNF2	NC_006363	87093	Circular	0					
***Nocardia* sp. 107**	pXT107	NC_010874	4335	Circular	0					
***Nocardia* sp. C-14-1**	pC1	NC_013538	5841	Circular	0					
***Rhodococcus aetherivorans* I24**	pRA1	NC_010882	9372	Circular	0					
***Rhodococcus equi* 103**	p103	NC_002576	80609	Circular	0					
***Rhodococcus equi* ATCC33701**	pREAT701	NC_004854	80610	Circular	0					
Rhodococcus equi	pVAPB1593	NC_011150	79251	Circular	0					
	pVAPA1037	NC_011151	80610	Circular	0					
	pVAPAMBE116	NC_014247	83100	Circular	0					
Rhodococcus erythropolis	pBD2	NC_005073	210205	Linear	0					
	pFAJ2600	NC_003846	5936	Circular	0					
	pRE8424	NC_006258	5987	Circular	0					
***Rhodococcus erythropolis* CCM2595**	pRECF1	NC_022125	90223	Circular	0					
**Rhodococcus *erythropolis* PR4**	pREL1	NC_007491	271577	Linear	0					
	pREC1	NC_007486	104014	Circular	0					
	pREC2	NC_007487	3637	Circular	0					
***Rhodococcus fascians* D188**	pFiD188	NC_021080	198917	Linear	0					
***Rhodococcus jostii* RHA1**	pRHL1	NC_008269	1123075	Linear	2	Q	452661-481845	29.1	25	65.81
						N	570289-600035	29.7	15	63.16
	pRHL2	NC_008270	442536	Linear	0					
	pRHL3	NC_008271	332361	Linear	0					
***Rhodococcus opacus* B4**	pKNR01	NC_006969	4367	Circular	0					
	pKNR02	NC_006970	2773	Circular	0					
	pROB01	NC_012520	558192	Linear	0					
	pROB02	NC_012521	244997	Linear	1	Y	63094-91700	28.6	14	65.14
	pKNR	NC_012523	111160	Circular	0					
***Rhodococcus pyridinivorans* SB3094**	plasmid	NC_023144	361397	Circular	0					
	plasmid	NC_023145	2035	Circular	0					
Rhodococcus rhodochrous	pNC500	NC_008823	7637	Circular	0					
***Rhodococcus* sp. B264-1**	pB264	NC_004900	4970	Circular	0					
***Rhodococcus* sp. BCP1**	pBMC1	NZ_CM002178	120373	Linear	0					
	pBMC2	NZ_CM002179	103129	Linear	0					
***Rhodococcus* sp. NS1**	pNSL1	NC_010850	117252	Linear	0					
***Tsukamurella paurometabola* DSM 20162**	pTpau01	NC_014159	99806	Circular	0					

[a] Y indicates Yes, N indicates No, Q indicates incomplete
[b] Coordinates for draft sequences are based on concatenation of all contigs by PHAST
