# Supplementary material for: Locating and Activating Molecular ‘Time Bombs’: Induction of Mycolata Prophages
Source: PLoS One. 2016 Aug 3;11(8):e0159957. doi: 10.1371/journal.pone.0159957 (PMC4972346; doi:10.1371/journal.pone.0159957)
Supplement: S3 Table — (PDF) [file pone.0159957.s003.pdf]

<u>**S3 Table: GAL1, GMA1, and TPA4 genome sequence annotations**</u>

ORF[a]	Coordinates	Size (aa)	Significant match [b]	% identity [c]	E value [d]	Putative function (conserved motif) [e]
**GAL1-*orf1***	8..706	233	hypothetical protein [*Gordonia alkanivorans*]	99	2e-144	HNH endocnuclease (pfam01844)
**GAL1-*orf2***	702..1169	156	hypothetical protein [*Gordonia alkanivorans*]	100	7e-102	
**GAL1-*orf3***	1300..1620	107	hypothetical protein [*Gordonia alkanivorans*]	100	5e-63	Putative small terminase
**GAL1-*orf4***	1617..3242	542	terminase [*Gordonia alkanivorans*]	99	0.0	Large terminase (COG4626)
**GAL1-*orf5***	3245..4624	460	hypothetical protein [*Gordonia alkanivorans*]	100	0.0	Portal protein (pfam05133)
**GAL1-*orf6***	4710..5651	314	hypothetical protein [*Gordonia alkanivorans*]	100	0.0	
**GAL1-*orf7***	5721..6392	224	hypothetical protein [*Gordonia alkanivorans*]	99	1e-144	
**GAL1-*orf8***	6396..6800	135	putative K structural protein [*Gordonia alkanivorans*]	100	7e-84	Bacteriophage lambda head decorator protein (pfam02924)
**GAL1-*orf9***	6815..7852	346	putative phage structural protein [*Gordonia alkanivorans*]	100	0.0	Phage major capsid protein E (pfam03864)
**GAL1-*orf10***	7852..8169	106	hypothetical protein [*Gordonia alkanivorans*]	100	2e-62	
**GAL1-*orf11***	8169..8561	131	hypothetical protein [*Gordonia alkanivorans*]	100	1e-81	
**GAL1-*orf12***	8558..8902	115	hypothetical protein [*Gordonia alkanivorans*]	100	2e-68	
**GAL1-*orf13***	8895..9233	113	hypothetical protein [*Gordonia alkanivorans*]	99	4e-66	
**GAL1-*orf14***	9230..9691	154	hypothetical protein [*Gordonia alkanivorans*]	100	1e-97	
**GAL1-*orf15***	9773..10699	309	hypothetical protein [*Gordonia alkanivorans*]	100	0.0	Putative major tail protein
**GAL1-*orf16***	10786..11244	153	hypothetical protein [*Gordonia alkanivorans*]	100	2e-95	Putative tail assembly protein
**GAL1-*orf17***	11226..11669	148	hypothetical protein [*Gordonia alkanivorans*]	99	9e-84	Putative tail assembly protein translated by conserved programmed translational frameshift
**GAL1-*orf18***	11671..16068	1466	hypothetical protein [*Gordonia alkanivorans*]	99	0.0	Putative tape measure protein (pfam01576; COG5412)
**GAL1-*orf19***	16068..16979	304	hypothetical protein [*Gordonia alkanivorans*]	100	0.0	
**GAL1-*orf20***	16985..18748	588	hypothetical protein [*Gordonia alkanivorans*]	99	0.0	
**GAL1-*orf21***	18745..19113	123	hypothetical protein [*Gordonia alkanivorans*]	99	4e-72	
**GAL1-*orf22***	19182..20282	367	hypothetical protein [*Gordonia alkanivorans*]	100	0.0	Lysin (pfam01510; pfam08310)
**GAL1-*orf23***	20309..20548	80	holin [*Gordonia alkanivorans*]	100	1e-41	Putative Holin
**GAL1-*orf24***	20564..20953	130	membrane protein [*Gordonia alkanivorans*]	100	3e-79	
**GAL1-*orf25***	20950..21339	130	hypothetical protein [*Gordonia alkanivorans*]	99	2e-74	
**GAL1-*orf26***	21339..22043	235	hypothetical protein [*Gordonia alkanivorans*]	100	3e-153	PE-PPE domain (pfam08237)
**GAL1-*orf27***	22156..22695	180	hypothetical protein [*Gordonia alkanivorans*]	99	4e-118	HNH endonuclease (pfam13392; pfam07463)
**GAL1-*orf28***	22722..24629	636	hypothetical protein [*Gordonia alkanivorans*]	99	0.0	

ORF[a]	Coordinates	Size (aa)	Significant match [b]	% identity [c]	E value [d]	Putative function (conserved motif) [e]
**GAL1-*orf29***	24641..26770	710	hypothetical protein [*Gordonia alkanivorans*]	100	0.0	
**GAL1-*orf30***	26783..27679	299	hypothetical protein [*Gordonia alkanivorans*]	100	0.0	
**GAL1-*orf31***	28103..29317	405	putative recombinase [*Gordonia alkanivorans*]	100	0.0	Integrase (pfam00589; pfam14659)
**GAL1-*orf32***	29336..29785	150	hypothetical protein GOALK_093_00780 [*Gordonia alkanivorans* NBRC 16433]	98	4e-35	
**GAL1-*orf33***	complement(29687..30364)	226	hypothetical protein [*Gordonia alkanivorans*]	99	1e-147	HTH DNA binding (pfam12844)
**GAL1-*orf34***	30600..31448	283	putative phage protein [*Gordonia alkanivorans*]	100	0.0	Rha phage regulatory protein (pfam09669)
**GAL1-*orf35***	31502..31741	80	hypothetical protein [*Gordonia alkanivorans*]	99	8e-44	HTH DNA binding (pfam01381)
**GAL1-*orf36***	31738..31854	39	-			
**GAL1-*orf37***	31854..31991	46	hypothetical protein [*Gordonia alkanivorans*]	100	2e-22	
**GAL1-*orf38***	32000..32320	107	hypothetical protein [*Gordonia alkanivorans*]	100	8e-65	
**GAL1-*orf39***	32334..33173	280	hypothetical protein [*Gordonia alkanivorans*]	99	1e-179	Phage antirepressor protein (pfam02498; pfam03374)
**GAL1-*orf40***	33170..33433	88	hypothetical protein [*Gordonia alkanivorans*]	99	7e-50	
**GAL1-*orf41***	33430..33756	109	putative Xre family DNA-binding protein [*Gordonia alkanivorans*]	100	7e-646	HTH DNA binding (pfam13560)
**GAL1-*orf42***	33753..33974	74	hypothetical protein [*Gordonia alkanivorans*]	99	1e-40	HTH DNA binding (pfam12728)
**GAL1-*orf43***	33971..34180	70	hypothetical protein [*Gordonia alkanivorans*]	100	2e-37	
**GAL1-*orf44***	34177..34359	61	hypothetical protein [*Gordonia alkanivorans*]	100	2e-33	
**GAL1-*orf45***	34356..34607	84	hypothetical protein [*Gordonia alkanivorans*]	100	2e-49	
**GAL1-*orf46***	34604..34783	60	hypothetical protein [*Gordonia alkanivorans*]	100	5e-32	
**GAL1-*orf47***	34770..35021	84	putative protocatechuate 3,4-dioxygenase subunit beta [*Gordonia alkanivorans*]	99	1e-45	Putative protocatechuate 3,4-dioxygenase subunit beta
**GAL1-*orf48***	35018..35191	58	-			
**GAL1-*orf49***	35188..35370	61	hypothetical protein [*Gordonia alkanivorans*]	100	2e-34	
**GAL1-*orf50***	35370..35588	73	putative pyruvate phosphate dikinase [*Gordonia alkanivorans*]	100	5e-42	Putative pyruvate phosphate dikinase
**GAL1-*orf51***	35588..36184	199	hypothetical protein [*Gordonia alkanivorans*]	100	2e-128	
**GAL1-*orf52***	36181..36321	47	-			
**GAL1-*orf53***	36318..37556	413	putative WhiB family regulatory protein [*Gordonia alkanivorans*]	100	0.0	WhiB (pfam02467)
**GAL1-*orf54***	37553..37900	116	hypothetical protein [*Gordonia alkanivorans*]	100	1e-71	
**GAL1-*orf55***	38002..38268	89	hypothetical protein [*Gordonia alkanivorans*]	98	3e-29	
**GAL1-*orf56***	38270..38929	220	putative methyltransferase [*Gordonia alkanivorans*]	100	1e-144	DNA methyltransferase (pfam01555; COG0863)
**GAL1-*orf57***	38966..39451	162	hypothetical protein [*Gordonia alkanivorans*]	100	2e-101	
**GAL1-*orf58***	39448..39708	87	hypothetical protein [*Gordonia alkanivorans*]	99	5e-49	

ORF[a]	Coordinates	Size (aa)	Significant match [b]	% identity [c]	E value [d]	Putative function (conserved motif) [e]
**GAL1-*orf59***	39705..39854	50	hypothetical protein [*Gordonia alkanivorans*]	100	5e-25	
**GAL1-*orf60***	39851..40093	81	hypothetical protein [*Gordonia alkanivorans*]	100	2e-47	
**GAL1-*orf61***	40090..40473	128	hypothetical protein [*Gordonia alkanivorans*]	100	2e-77	
**GAL1-*orf62***	40466..40969	168	hypothetical protein [*Gordonia alkanivorans*]	100	6e-104	
**GAL1-*orf63***	40966..41148	61	hypothetical protein [*Gordonia alkanivorans*]	100	4e-33	
**GAL1-*orf64***	41145..41696	184	methyltransferase [*Gordonia alkanivorans*]	100	4e-121	DNA N-6-adenine-methyltransferase (pfam05869)
**GAL1-*orf65***	41697..42014	106	hypothetical protein [*Gordonia alkanivorans*]	100	3e-56	
**GAL1-*orf66***	42011..42133	41	-			
**GAL1-*orf67***	42130..42576	149	hypothetical protein [*Gordonia alkanivorans*]	100	1e-94	
**GAL1-*orf68***	42573..43475	301	hypothetical protein [*Gordonia alkanivorans*]	99	0.0	
**GAL1-*orf69***	43472..43843	124	hypothetical protein [*Gordonia alkanivorans*]	100	1e-76	
**GAL1-*orf70***	43840..44205	122	hypothetical protein [*Gordonia alkanivorans*]	100	2e-74	Endodeoxyribonuclease (pfam05866)
**GAL1-*orf71***	44202..44411	70	hypothetical protein [*Gordonia alkanivorans*]	100	1e-38	
**GAL1-*orf72***	44515..45402	296	hypothetical protein [*Gordonia alkanivorans*]	100	0.0	Unknown (DUF3310)
**GAL1-*orf73***	45399..45611	71	hypothetical protein [*Gordonia alkanivorans*]	99	2e-40	
**GAL1-*orf74***	45602..45817	72	hypothetical protein [*Gordonia alkanivorans*]	100	3e-40	
**GAL1-*orf75***	45814..46038	75	hypothetical protein [*Gordonia alkanivorans*]	99	2e-43	
**GAL1-*orf76***	46097..46876	260	hypothetical protein [*Gordonia alkanivorans*]	100	1e-173	
**GAL1-*orf77***	47220..47414	65	hypothetical protein [*Gordonia alkanivorans*]	100	5e-34	
**GAL1-*orf78***	47436..47828	131	hypothetical protein [*Gordonia alkanivorans*]	100	2e-80	Unknown (pfam11750)
**GAL1-*orf79***	47825..48118	98	hypothetical protein [*Gordonia alkanivorans*]	100	7e-59	
**GAL1-*orf80***	48135..48575	147	putative gluconate 2-dehydrogenase (acceptor) [*Gordonia alkanivorans*]	100	2e-92	Putative gluconate 2-dehydrogenase
**GAL1-*orf81***	48654..49394	247	hypothetical protein [*Gordonia alkanivorans*]	100	5e-164	HNH endonuclease (pfam01844)
**GAL1-*orf82***	49391..49576	62	hypothetical protein [*Gordonia alkanivorans*]	98	7e-34	
**GAL1-*orf83***	49576..49935	120	hypothetical protein [*Gordonia alkanivorans*]	100	3e-75	Restriction endonuclease (COG1403)
**GMA1-*orf1***	78..440	121	hypothetical protein EN35_19995 [*Rhodococcus qingshengii*]	39	4e-10	Putative small terminase subunit
**GMA1-*orf2***	437..1684	416	terminase large subunit [*Rhodococcus equi*]	60	4e-172	Large terminase subunit (pfam04466)
**GMA1-*orf3***	1681..3249	523	hypothetical protein [*Rhodococcus* sp. UNC363MFTsu5.1]	46	2e-136	Phage portal protein (pfam05133)
**GMA1-*orf4***	3255..4343	363	phage minor capsid protein 2 [*Streptomyces globisporus*]	40	1e-71	Phage minor capsid protein (pfam06152)
**GMA1-*orf5***	4631..5179	183	hypothetical protein [*Rhodococcus* sp. UNC363MFTsu5.1]	47	1e-29	

ORF[a]	Coordinates	Size (aa)	Significant match [b]	% identity [c]	E value [d]	Putative function (conserved motif) [e]
**GMA1-*orf6***	5231..6211	327	phage capsid protein [*Rhodococcus* sp. UNC363MFTsu5.1]	69	3e-150	Putative phage capsid protein
**GMA1-*orf7***	6211..6375	55	hypothetical protein [*Streptomyces* sp. NRRL WC-3795]	53	3e-08	
**GMA1-*orf8***	6454..6870	139	hypothetical protein EN35_19955 [*Rhodococcus qingshengii*]	55	2e-36	
**GMA1-*orf9***	6867..7187	107	hypothetical protein [*Rhodococcus* sp. UNC363MFTsu5.1]	44	3e-17	
**GMA1-*orf10***	7189..7524	112	hypothetical protein [*Corynebacterium aurimucosum*]	41	1e-17	
**GMA1-*orf11***	7533..7808	92	-			
**GMA1-*orf12***	7805..8254	150	hypothetical protein QR64_00255 [*Rhodococcus* sp. Chr-9]	42	2e-21	
**GMA1-*orf13***	8266..8748	161	hypothetical protein [*Rhodococcus fascians*]	42	4e-35	Putative major tail protein
**GMA1-*orf14***	8784..9359	192	hypothetical protein [*Rhodococcus fascians*]	38	8e-23	Putative tail assembly protein
**GMA1-*orf15***	9341..9703	121	hypothetical protein [*Rhodococcus* sp. p52]	52	2e-16	Putative tail assembly protein translated by conserved programmed translational frameshift
**GMA1-*orf16***	complement(9700..10146)	149	-			
**GMA1-*orf17***	10196..15121	1642	hypothetical protein [*Rhodococcus fascians*]	32	4e-104	Tape measure protein (pfam06737)
**GMA1-*orf18***	15118..15930	271	hypothetical protein [*Rhodococcus* sp. 29MFTsu3.1]	29	8e-28	
**GMA1-*orf19***	15927..17480	518	hypothetical protein [*Rhodococcus* sp. p52]	44	6e-121	
**GMA1-*orf20***	17561..18616	352	hypothetical protein [*Gordonia malaquae*]	53	4e-115	Lysin (pfam01510; pfam08310 X2)
**GMA1-*orf21***	18613..18861	83	holin [*Dietzia alimentaria*]	58	3e-20	Putative holin
**GMA1-*orf22***	18858..19307	150	gp15 [*Mycobacterium* phage Dori]	38	3e-16	
**GMA1-*orf23***	19297..19617	107	-			
**GMA1-*orf24***	19617..20363	249	hypothetical protein [*Gordonia sihwensis*]	48	2e-49	
**GMA1-*orf25***	20374..20823	150	hypothetical protein [*Gordonia sihwensis*]	50	9e-37	
**GMA1-*orf26***	20820..21035	72	hypothetical protein [*Gordonia sihwensis*]	41	1e-06	
**GMA1-*orf27***	21102..21533	144	hypothetical protein [*Rhodococcus equi*]	63	1e-58	
**GMA1-*orf28***	21554..22813	420	hypothetical protein [*Gordonia soli*]	41	3e-24	
**GMA1-*orf29***	22915..23067	51	hypothetical protein [*Gordonia malaquae*]	91	3e-21	
**GMA1-*orf30***	complement(23064..24152)	363	site-specific recombinase XerD [*Mycobacterium smegmatis*]	48	2e-106	Phage integrase (COG0582)
**GMA1-*orf31***	complement(24478..24915)	146	hypothetical protein [*Gordonia malaquae*]	99	7e-69	
**GMA1-*orf32***	complement(24955..25266)	104	hypothetical protein [*Glycomyces arizonensis*]	35	2e-05	
**GMA1-*orf33***	complement(25267..25485)	73	hypothetical protein [*Nocardiopsis dassonvillei*]	53	4e-18	

ORF[a]	Coordinates	Size (aa)	Significant match [b]	% identity [c]	E value [d]	Putative function (conserved motif) [e]
**GMA1-*orf34***	complement(25482..26489)	336	hypothetical protein [*Gordonia malaquae*]	64	1e-23	
**GMA1-*orf35***	26567..26794	76	gp41 [*Mycobacterium* phage PMC]	67	1e-16	HTH DNA binding (pfam01381)
**GMA1-*orf36***	26839..27690	284	putative phage protein [*Gordonia alkanivorans*]	87	2e-78	BRO family N-terminal protein (pfam02498)
**GMA1-*orf37***	27722..28042	107	hypothetical protein [*Gordonia malaquae*]	44	2e-09	
**GMA1-*orf38***	28039..28158	40	-			
**GMA1-*orf39***	28155..28403	83	-			
**GMA1-*orf40***	28400..28561	54	-			
**GMA1-*orf41***	28629..28913	95	hypothetical protein [*Gordonia malaquae*]	94	6e-55	
**GMA1-*orf42***	28910..29065	52	hypothetical protein [*Gordonia malaquae*]	88	2e-18	
**GMA1-*orf43***	29052..29600	183	hypothetical protein [*Gordonia malaquae*]	73	5e-77	
**GMA1-*orf44***	29606..29722	39	hypothetical protein [*Gordonia malaquae*]	93	1e-08	
**GMA1-*orf45***	29719..29916	66	-			
**GMA1-*orf46***	29913..30710	266	hypothetical protein [*Gordonia malaquae*]	97	0.0	Exonuclease (PRK09709)
**GMA1-*orf47***	30722..31246	175	hypothetical protein [*Gordonia malaquae*]	100	2e-124	Endonuclease (pfam07463; pfam13392)
**GMA1-*orf48***	31243..32121	293	hypothetical protein [*Gordonia malaquae*]	98	0.0	
**GMA1-*orf49***	32118..32558	147	transcription factor WhiB [*Gordonia sputi*]	43	9e-09	Transcription factor for WhiB (pfam02467)
**GMA1-*orf50***	32555..32782	76	-			
**GMA1-*orf51***	32775..32900	42	-			
**GMA1-*orf52***	32897..33145	83	gp86 [*Mycobacterium* phage Gumball]	47	1e-10	
**GMA1-*orf53***	33138..33686	183	hypothetical protein [*Rhodococcus* sp. P27]	51	2e-16	
**GMA1-*orf54***	33679..33984	102	hypothetical protein [*Gordonia malaquae*]	86	6e-37	
**GMA1-*orf55***	complement(33988..34329)	114	hypothetical protein [*Gordonia malaquae*]	91	5e-55	
**GMA1-*orf56***	complement(34377..34490)	38	hypothetical protein [*Streptomyces turgidiscabies*]	68	5e-05	
**GMA1-*orf57***	complement(34544..34753)	70	-			
**GMA1-*orf58***	34814..35008	65	-			
**GMA1-*orf59***	35005..35238	78	hypothetical protein [*Gordonia sihwensis*]	44	3e-06	
**GMA1-*orf60***	35328..35543	72	-			
**GMA1-*orf61***	35540..35677	46	-			
**GMA1-*orf62***	35817..37163	449	Gp65 [*Rhodococcus ruber*]	74	0.0	Helicase (pfam00271; pfam00176)
**GMA1-*orf63***	37160..38131	324	Gp66 [*Rhodococcus ruber*]	80	0.0	Methylase (pfam01555)
**GMA1-*orf64***	38079..38942	288	hypothetical protein GSI01S_10_02210 [*Gordonia sihwensis* NBRC 108236]	67	3e-121	
**GMA1-*orf65***	39076..39228	51	-			

ORF[a]	Coordinates	Size (aa)	Significant match [b]	% identity [c]	E value [d]	Putative function (conserved motif) [e]
**GMA1-*orf66***	39225..39875	217	hypothetical protein [*Gordonia sihwensis*]	44	4e-40	
**GMA1-*orf67***	40045..40320	92	hypothetical protein [*Gordonia malaquae*]	71	6e-29	
**GMA1-*orf68***	40382..40945	188	gp54 [*Mycobacterium* phage Mutaforma13]	47	2e-36	Endonuclease (pfam07463; pfam13392)
**TPA4-*orf1***	75..497	141	hypothetical protein N505_0105320 [*Rhodococcus* sp. BCP1]	62	7e-49	Putative small terminase
**TPA4-*orf2***	526..2238	571	terminase [*Rhodococcus* sp. BCP1]	83	0.0	Putative large terminase
**TPA4-*orf3***	2235..3725	497	hypothetical protein [*Gordonia sihwensis*]	59	0.0	Portal protein (pfam05133)
**TPA4-*orf4***	3732..4874	381	capsid maturation protease [*Mycobacterium* phage ZoeJ]	43	2e-46	Capsid maturation protease (cd13442)
**TPA4-*orf5***	4871..5194	108	-			
**TPA4-*orf6***	5313..5945	211	hypothetical protein [*Gordonia sihwensis*]	42	2e-14	
**TPA4-*orf7***	5997..6371	125	hypothetical protein [*Gordonia sihwensis*]	74	2e-59	
**TPA4-*orf8***	6373..7398	342	hypothetical protein [*Gordonia sihwensis*]	68	5e-156	Major capsid protein (pfam03864)
**TPA4-*orf9***	7567..7953	129	hypothetical protein [*Gordonia sihwensis*]	42	1e-20	
**TPA4-*orf10***	7947..8351	135	hypothetical protein [*Mycobacterium abscessus*]	41	6e-21	
**TPA4-*orf11***	8351..8638	96	hypothetical protein [*Mycobacterium abscessus*]	45	2e-11	
**TPA4-*orf12***	8635..9036	134	hypothetical protein [*Mycobacterium abscessus*]	30	2e-04	
**TPA4-*orf13***	9127..9984	286	hypothetical protein I544_3152 [*Mycobacterium abscessus* 103]	46	4e-73	
**TPA4-*orf14***	complement(10033..10527)	165	hypothetical protein [*Dietzia alimentaria*]	61	1e-33	
**TPA4-*orf15***	10600..11064	155	hypothetical protein [*Mycobacterium* sp. 141]	46	5e-22	Putative tail assembly protein
**TPA4-*orf16***	11046..11465	140	Hypothetical protein BB31_24245 [*Amcolatopsis lurida* NRRL 2430]	33	8e-05	Putative tail assembly protein translated by conserved programmed translational frameshift
**TPA4-*orf17***	11473..16632	1720	hypothetical protein [*Mycobacterium* sp. 141]	37	2e-137	Tape measure protein (COG1196)
**TPA4-*orf18***	16629..17726	366	putative gp22 [*Mycobacterium abscessus* MAB_110811_2726]	63	4e-164	
**TPA4-*orf19***	17726..19462	579	putative gp23 [*Mycobacterium abscessus* MAB_110811_2726]	74	0.0	
**TPA4-*orf20***	19528..19722	65	-			
**TPA4-*orf21***	19719..20168	150	putative gp24 [*Mycobacterium abscessus* MAB_110811_2726]	50	2e-31	
**TPA4-*orf22***	20165..21250	362	tail protein [*Mycobacterium abscessus*]	54	1e-120	Putative tail protein
**TPA4-*orf23***	21250..21615	122	hypothetical protein [*Mycobacterium abscessus*]	59	2e-27	
**TPA4-*orf24***	21615..23492	626	putative structural protein [*Mycobacterium* phage WIVsmall]	39	2e-99	Putative structural protein
**TPA4-*orf25***	23492..23755	88	-			
**TPA4-*orf26***	23759..25357	533	putative structural protein [*Mycobacterium* phage	28	1e-06	Putative structural protein

ORF[a]	Coordinates	Size (aa)	Significant match [b]	% identity [c]	E value [d]	Putative function (conserved motif) [e]
			WIVsmall]			
**TPA4-*orf27***	25382..25834	151	hypothetical protein WIVsmall_58 [*Mycobacterium* phage WIVsmall]	74	1e-74	
**TPA4-*orf28***	25836..26891	352	hypothetical protein [*Proteobacteria bacterium* JGI 0000113-L05]	51	5e-16	
**TPA4-*orf29***	26891..27490	200	hypothetical protein [*Gordonia sihwensis*]	51	1e-09	
**TPA4-*orf30***	27688..28482	265	hypothetical protein [*Nocardia* sp. BMG111209]	36	4e-19	PE-PPE domain (pfam08237)
**TPA4-*orf31***	28502..29614	371	antigen 85 complex protein [*Rhodococcus rhodnii*]	54	2e-122	Lysin (pfam01510; pfam08310)
**TPA4-*orf32***	29620..29796	59	-			
**TPA4-*orf33***	29799..30185	129	hypothetical protein TM4_gp31 [*Mycobacterium* phage TM4]	31	4e-13	
**TPA4-*orf34***	30182..30700	173	hypothetical protein [*Gordonia malaquae*]	33	4e-13	
**TPA4-*orf35***	complement(30775..30942)	56	hypothetical protein [*Gordonia soli*]	45	4e-05	
**TPA4-*orf36***	31015..31287	91	-			
**TPA4-*orf37***	complement(31376..32608)	411	Integrase [*Gordonia* sp. KTR9]	53	1e-134	Integrase (pfam14659; pfam00589)
**TPA4-*orf38***	complement(32979..33404)	142	hypothetical protein RR21198_4189 [*Rhodococcus rhodochrous* ATCC 21198]	47	3e-17	
**TPA4-*orf39***	complement(33675..33860)	62	-			
**TPA4-*orf40***	complement(33956..34099)	48	-			
**TPA4-*orf41***	34071..34304	78	hypothetical protein [*Dietzia* sp. UCD-THP]	55	1e-12	
**TPA4-*orf42***	34301..34543	81	-			
**TPA4-*orf43***	34540..34899	120	hypothetical protein [*Gordonia amarae*]	52	3e-17	
**TPA4-*orf44***	34941..35237	99	-			
**TPA4-*orf45***	35234..35554	107	hypothetical protein [*Mycobacterium avium*]	33	6e-04	
**TPA4-*orf46***	35551..36090	180	-			
**TPA4-*orf47***	36087..36890	268	hypothetical protein PBI_CATDAWG_12 [*Mycobacterium* phage Catdawg]	41	7e-53	CRISPR-associated Cas4-like protein (PHA00619)
**TPA4-*orf48***	36926..37252	109	hypothetical protein [*Mycobacterium* sp. UM_RHS]	59	2e-39	
**TPA4-*orf49***	37276..38109	278	gp66 [*Mycobacterium* phage Dori]	56	6e-102	Unknown (pfam10065)
**TPA4-*orf50***	38139..38771	211	hypothetical protein [*Salinispora pacifica*]	41	8e-24	
**TPA4-*orf51***	38771..38914	48	-			
**TPA4-*orf52***	38998..39378	127	-			
**TPA4-*orf53***	39393..39623	77	hypothetical protein [*Nocardiopsis gilva*]	54	1e-12	
**TPA4-*orf54***	39624..39896	91	-			
**TPA4-*orf55***	39893..40261	123	hypothetical protein TPA2_gp62 [*Tsukamurella* phage TPA2]	57	7e-29	

ORF[a]	Coordinates	Size (aa)	Significant match [b]	% identity [c]	E value [d]	Putative function (conserved motif) [e]
**TPA4-*orf56***	40258..40635	126	-			
**TPA4-*orf57***	40632..41162	177	hypothetical protein [*Mycobacterium* sp. UM_WWY]	39	1e-16	Exonuclease (cd06127)
**TPA4-*orf58***	41159..41554	132	hypothetical protein [*Gordonia otitidis*]	67	3e-45	
**TPA4-*orf59***	41855..41971	39	-			
**TPA4-*orf60***	41974..42615	214	hypothetical protein PBI_DONOVAN_52 [*Mycobacterium* phage Donovan]	40	3e-32	
**TPA4-*orf61***	42608..42820	71	-			
**TPA4-*orf62***	42817..43089	91	-			
**TPA4-*orf63***	43272..43652	127	hypothetical protein [*Mycobacterium abscessus*]	60	9e-33	
**TPA4-*orf64***	43645..44205	187	hypothetical protein [*Nocardia nova*]	69	6e-14	
**TPA4-*orf65***	44205..44390	62	-			
**TPA4-*orf66***	complement(44357..44842)	162	-			
**TPA4-*orf67***	44841..44984	48	-			
**TPA4-*orf68***	44981..45280	100	-			
**TPA4-*orf69***	45280..45519	80	-			
**TPA4-*orf70***	45516..45704	63	-			
**TPA4-*orf71***	45701..45991	97	gp79 [*Mycobacterium* phage Dori]	49	2e-04	
**TPA4-*orf72***	45988..46362	125	sporulation protein [*Caldanaerobacter subterraneus*]	44	1e-04	WhiA C-terminal HTH domain (pfam02650)
**TPA4-*orf73***	46359..46703	115	WhiB family transcriptional regulator [*Corynebacterium efficiens*]	55	6e-24	WhiB transcription factor (pfam02467)
**TPA4-*orf74***	46700..47062	121	-			
**TPA4-*orf75***	47059..48669	537	DNA methyltransferase [*Mycobacterium abscessus*]	65	0.0	DNA Methylase (pfam00145; cd00315)
**TPA4-*orf76***	48666..49040	125	hypothetical protein [*Nocardia brasiliensis*]	53	3e-25	
**TPA4-*orf77***	49037..51694	886	gp85 [*Mycobacterium* phage DS6A]	38	3e-62	
**TPA4-*orf78***	51945..52427	161	hypothetical protein [*Mycobacterium abscessus*]	49	8e-37	Recombination endonuclease (pfam02945)
**TPA4-*orf79***	52424..52702	93	-			
**TPA4-*orf80***	52699..53697	333	hypothetical protein [*Nocardia farcinica*]	52	2e-18	
**TPA4-*orf81***	53694..53990	99	-			
**TPA4-*orf82***	53987..54289	101	hypothetical protein [*Tsukamurella* sp. 1534]	45	1e-12	
**TPA4-*orf83***	complement(54265..55047)	261	-			
**TPA4-*orf84***	55293..55478	62	-			
**TPA4-*orf85***	55475..55669	65	-			
**TPA4-*orf86***	55842..56165	108	hypothetical protein [*Gordonia malaquae*]	53	1e-24	

[a] ORFs were numbered consecutively, [b] The most closely related gene (only if named) and the name of the organism, [c] Percentage identity is based on the best match when a BLAST P analysis is performed, [d] The probability of obtaining a match by chance as determined by BLAST analysis. Only values less than 10^{-4} were considered significant, [e] Predicted function is based on amino acid identity, conserved motifs, and gene location within functional modules
