# Supplementary material for: Locating and Activating Molecular ‘Time Bombs’: Induction of Mycolata Prophages
Source: PLoS One. 2016 Aug 3;11(8):e0159957. doi: 10.1371/journal.pone.0159957 (PMC4972346; doi:10.1371/journal.pone.0159957)
Supplement: S4 Table — (PDF) [file pone.0159957.s004.pdf]

S4 Table: Palindromes in the genome sequence of phages GAL1, GMA1, and TPA4

Repeat number-Phage	Size (bp)	Coordinates	Sequence alignment	Position
**P1-GAL1**	36	27770-27804	CCGCCCCCACTCTTC-ACGTCGGAGAGTGGGGGCGG	Between *orf30* and *orf31*
		27804-27770	CCGCCCCCACTCTCCGACGT-GAAGAGTGGGGGCGG	
**P2-GAL1**	27	30767-30793	CTTGGTCGGATTTGAAATCCGACCAAG	Within *orf34*
		30793-30767	CTTGGTCGGATTTCAAATCCGACCAAG	
**P3-GAL1**	20	26325-26344	CAGCCGTCGATCGACGCCTG	Within *orf29*
		26344-26325	CAGGCGTCGATCGACGGCTG	
**P4-GAL1**	18	23320-23337	GCAGCGGCCGGCCGCTGC	Within *orf28*
		23337-23320	GCAGCGGCCGGCCGCTGC	
**P1-GMA1**	39	6388-6426	CAGCCCGGCACCGTGTGGCCCTCCCTCGGTGCCGGGCTG	Between *orf7* and *orf8*
		6426-6388	CAGCCCGGCACCGAGGGAGGGCCACACGGTGCCGGGCTG	
**P2-GMA1**	18	21453-21470	GGTGCTGACGTCAGCACC	Within *orf27*
		21470-21453	GGTGCTGACGTCAGCACC	
**P3-GMA1**	16	27417-27432	CTGGTTGATCAACCAG	Within *orf36*
		27432-27417	CTGGTTGATCAACCAG	
**P4-GMA1**	16	32932-32947	GCTGGCCGCGGCCAGC	Within *orf52*
		32947-32932	GCTGGCCGCGGCCAGC	
**P1-TPA4**	86	4475-4556	ACCGGGCCGCGTCGACC--GATCGAAG-GTCCGGAACGTGCTGCAGCACGAGCTCGACACTGCGAACCGGGTCG-CTCGGCTCGGT	Within *orf4*
		4556-4475	ACCGAGCCGAG-CGACCCGGTTCGCAGTGTCGAGCTCGTGCTGCAGCACGTTCCGGAC-CTTCGATC--GGTCGACGCGGCCCGGT	
**P2-TPA4**	48	9987-10032	CTGGTG--GTGGCCGCGTAGCGGGGAAGCTACGCGGCCACCGCACCAG	Between *orf13* and *orf14*
		10032-9987	CTGGTGCGGTGGCCGCGTAGCTTCCCCGCTACGCGGCCAC--CACCAG	
**P3-TPA4**	46	38927-38970	GCCGCGGCCAGCAGACCGGCTTCAAAACC--TCTGCTGGCCGCGGC	Between *orf51* and *orf52*
		38970-38927	GCCGCGGCCAGCAGA--GGTTTTGAAGCCGGTCTGCTGGCCGCGGC	
**P4-TPA4**	36	30268-30301	CGCGGCGGCGAT--CATCGCTGCCATCGCCGCCGCG	Within *orf34*
		30301-30268	CGCGGCGGCGATGGCAGCGATG--ATCGCCGCCGCG	
**P5-TPA4**	35	31306-31340	AGAAGCCCCCTCCGAGATTCTCGGAGGGGGCTTCT	Between *orf36* and *orf37*
		31340-31306	AGAAGCCCCCTCCGAGAATCTCGGAGGGGGCTTCT	
**P6-TPA4**	35	7620-7654	CCTCGAGGAGGCCCGCACCACGGGCCTCCTCGAGG	Within *orf9*

Repeat number-Phage	Size (bp)	Coordinates	Sequence alignment	Position
		7654-7620	CCTCGAGGAGGCCCGTGGTGCGGGCCTCCTCGAGG	
**P7-TPA4**	32	27504-27535	CGTGCCCCGCGGCCAGCGCGCCGCGGGGCACG	Between *orf29* and *orf30*
		27535-27504	CGTGCCCCGCGGCGCGCTGGCCGCGGGGCACG	
**P8-TPA4**	31	30720-30750	AGCGGCCCCGAGCATCACGCTCGGGGCCGCT	Between *orf34* and *orf35*
		30750-30720	AGCGGCCCCGAGCGTGATGCTCGGGGCCGCT	
**P9-TPA4**	25	51717-51741	GGTTCCGAGGGCGACCCTCGGAACC	Between *orf77* and *orf78*
		51741-51717	GGTTCCGAGGGTCGCCCTCGGAACC	
**P10-TPA4**	20	29014-29033	CCGCCGCGACGTCGCGGCGG	Within *orf31*
		29033-29014	CCGCCGCGACGTCGCGGCGG	
**P11-TPA4**	20	8401-8420	CGACGGCGTCGACGCCCTCG	Within *orf11*
		8420-8401	CGAGGGCGTCGACGCCGTCG	
**P12-TPA4**	16	38186-38201	CGGGAAGTACTTCCCG	Within *orf50*
		38201-38186	CGGGAAGTACTTCCCG	
**P13-TPA4**	16	45597-45612	CGCCGCCCGGGCGGCG	Within *orf70*
		45612-45597	CGCCGCCCGGGCGGCG	
