# Supplementary material for: Locating and Activating Molecular ‘Time Bombs’: Induction of Mycolata Prophages
Source: PLoS One. 2016 Aug 3;11(8):e0159957. doi: 10.1371/journal.pone.0159957 (PMC4972346; doi:10.1371/journal.pone.0159957)
Supplement: S5 Table — (PDF) [file pone.0159957.s005.pdf]

S5 Table: Repeats in the genome sequences of phages GAL1, GMA1, and TPA4. I indicates inverted repeat, D indicates direct repeat.

Phage-Repeat number	Size (bp)	Coordinates	Sequence alignment
**GAL1-D1**	70	41915-41984	CGACCAGTTGAAGGCGATCGGTAACGGGGTGTGCCCGCCGCAAGCGTTCCGGGCGTTGCAAATCCTGGAC
		31892-31961	CGATCAGCTGAAGGCCGTCGGCAACGGTGTGTGCCCGCCTCAGGCGTACCGGGCACTGGAACTGCTGCAC
**GAL1-D2**	41	17225-17265	GGCGACGACCTGGACGACTTCTGTTTGTGGGCGCGGGAGAT
		17003-17043	GGCGACGACATTGACGAGTTCTGCCAGTGGGCGCGGGACAT
**GAL1-D3**	31	28065-28095	CTGGGCTAAACGTAAGTACAAACGTAAGTGA
		27866-27896	CTGCGATAGACGTAAGTCTAAACGTAAGTGA
**GAL1-D4**	31	41061-41091	CACCGTCCGCAACGCCCTCTGGTCCAGCGGC
		35426-35456	CACCCTACGCATCGCCCTCTGGTGCGGAGGC
**GAL1-D5**	28	38946-38973	CACCGGGGCGGTGTACTGACATGAGCGA
		38250-38277	CATCGGGGCGGTGTACTGACATGGGCGA
**GAL1-D6**	28	26958-26985	GGTCGTTGCTCGCCACCACCGACGGCAC
		25629-25656	GGACGTTGTACGGCTCCACCGACGGCAC
**GAL1-D7**	26	39440-39465	GGTGTCCGGTGACCGCCGATCCCGCC
		38918-38943	GGTGTCCGGTGACCGCCGATCCCGCC
**GAL1-D8**	25	6568-6592	AGGTCACCGCGACCGGCCTGTTCGG
		6242-6265	AGGGCACCT-GACCGGCCTGTTCGG
**GAL1-D9**	25	26561-26584	GCCCGCAATCTCAACCCCG-GTGGT
		25562-25586	GCCCGCAATCTCATCCACGAGTGGT
**GAL1-D10**	21	35271-35291	CATCATCGACGGAGAGACGTG
		32441-32461	CATCATCGACGGCGAGCCGTG
**GAL1-D11**	21	37771-37791	GGCGTACGCGAAGGGCTGGTT
		37280-37300	GGCGAAGGCGAAGGGCTGGTT
**GAL1-D12**	21	47370-47390	ACCGACCACATCACGATCGCG
		44205-44225	ACCGACCACATCAGCATCGCG
**GAL1-D13**	20	19889-19908	CGGCCTGTTCGAGGCGTACG
		6581-6600	CGGCCTGTTCGGGCCGTACG
**GAL1-D14**	20	30736-30755	GCCACAACCTGGACGACTTC
		17226-17245	GCGACGACCTGGACGACTTC
**GAL1-D15**	20	46932-46951	TCCCCGTCGTCATCCGCGAC

Phage-Repeat number	Size (bp)	Coordinates	Sequence alignment
		24049-24068	TCCCCGTCGTCATCGTCGAC
**GAL1-D16**	20	43974-43993	CATCCGGCCGCTGACAGCCC
		26659-26678	CATCCGGCCGCTCACCGCCC
**GAL1-D17**	20	47822-47841	GCTATGACCACCACCGACCT
		39848-39867	GCTATGACCACCCCCAACCT
**GAL1-D18**	19	37488-37506	CGGCATGGTCGGCGGTGTC
		12354-12372	CGGCATCGTCGGCGGTGTC
**GAL1-D19**	19	42335-42353	GCTGGATCGGGTTCGACAC
		38786-38804	GCTGGATCGGGTTCCACAC
**GAL1-D20**	18	13705-13722	CCGGTCCTCGGGCAGCTG
		13393-13410	CCGTTCCTCGGGCAGCTG
**GAL1-D21**	18	15844-15861	GGCGAGTACGTGGTCAAC
		14512-14529	GGCGAGTACGTCGTCAAC
**GAL1-D22**	17	40090-40106	ATGACCACCCCCAACCT
		39851-39867	ATGACCACCCCCAACCT
**GAL1-D23**	16	9601-9616	GGAAACCCAGCAGCAG
		7357-7372	GGAAACCCAGCAGCAG
**GAL1-D24**	16	23976-23991	GCGCAGCGTGACGCCA
		12922-12937	GCGCAGCGTGACGCCA
**GAL1-D25**	16	32437-32452	CCGTCATCATCGACGG
		16894-16909	CCGTCATCATCGACGG
**GAL1-D26**	16	47860-47875	CGTGTCTGCCGCCTCG
		34060-34075	CGTGTCTGCCGCCTCG
**GAL1-D27**	16	43540-43555	CCGCATCGACGACCCC
		37325-37340	CCGCATCGACGACCCC
**GAL1-D28**	15	47445-47459	GCTGAGGCGATCGCC
		2358-2372	GCTGAGGCGATCGCC
**GAL1-D29**	15	33341-33355	CTCGAGATCGGCCCG
		10058-10072	CTCGAGATCGGCCCG
**GAL1-D30**	15	36652-36666	AGCACGGCGTCCTCG
		20152-20166	AGCACGGCGTCCTCG

Phage-Repeat number	Size (bp)	Coordinates	Sequence alignment
**GAL1-I1**	44	12142-12184	GGTCTGGATCGGCG-CATCAACATCAACGTCAACACCAGCGGCC
		5004-4961	GGTCGAGATCGGCCACACCATCGTCGACCTCAACACCAGCGGCC
**GAL1-I2**	43	30221-30260	GATGACGTTCTGCGCG---TCAGGGCGGTTCCTGCCGAGCTTC
		10950-10908	GATGACGTACGGCTCGAGTTCGACGCGGTTCTTGCCGAGCTTC
**GAL1-I3**	28	35789-35816	CCCATCCCCGGGCCGACGAGCTTCACCC
		13349-13323	CCCATCCCCGGGCCGGCG-GCGGCACCC
**GAL1-I4**	26	32493-32518	GGATACCGCGACGCGAGCAACGCAGC
		21565-21540	GGATACCGCGACGCGTGGACAGCAGC
**GAL1-I5**	23	37176-37198	GCGGGCCGGGTGGAGAACATCAC
		35040-35018	GCGTGGCGGGTGGAGAACGTCAC
**GAL1-I6**	20	40924-40942	GGTCG-CGCACCAACTCCTC
		20658-20639	GGTCGGCGCACCAACTCCTC
**GAL1-I7**	19	23906-23924	GATGCGCCCGTACTCGCCC
		7096-7078	GATGCGCTCGTACTCGCCC
**GAL1-I8**	18	18606-18623	TGTCGTTGCCGTTGGCGA
		7397-7380	TGCCGTTGCCGTTGGCGA
**GAL1-I9**	18	43961-43978	CGAAACAGGCCCGCATCC
		39435-39418	CGAAGCAGGCCCGCATCC
**GAL1-I10**	16	25447-25462	CACCCACGTCGACGGG
		17558-17543	CACCCACGTCGACGGG
**GAL1-I11**	16	41609-41624	CAAGGGTGACCGTCCG
		33577-33562	CAAGGGTGACCGTCCG
**GAL1-I12**	16	84-99	GGCACTCAGTGCCGGT
		49940-49925	GGCACTCAGTGCCGGT
**GAL1-I13**	15	7492-7506	GCGGCCGGCGGATCG
		680-666	GCGGCCGGCGGATCG
**GAL1-I14**	15	12653-12667	CAGCTGCAGCCGCAG
		5860-5846	CAGCTGCAGCCGCAG
**GAL1-I15**	15	8831-8845	AGGGTGACCGAGGTG
		6093-6079	AGGGTGACCGAGGTG
**GAL1-I16**	15	9271-9285	CGACATCGGTGATCT

Phage-Repeat number	Size (bp)	Coordinates	Sequence alignment
		8717-8703	CGACATCGGTGATCT
**GAL1-I17**	15	49695-49709	CACATCCACATCAGC
		12726-12712	CACATCCACATCAGC
**GAL1-I18**	15	21868-21882	TCGACAGCATCGCCG
		14509-14495	TCGACAGCATCGCCG
**GAL1-I19**	15	45708-45722	ACCCCGGCGGCTACG
		29106-29092	ACCCCGGCGGCTACG
**TPA4-D1**	193	15953-16143	CGAAGCAGATCCGCGACGCCGAGCAGAAGGTCGCCGACAAGGAGGCCGCCGCGCGGCGCGCGCGCACCCAGCTCGCGGAGGCGC GGAACAACCCGAAGGCGAAGAAGTCGATGATC-- CAGGCCGCCGAGGACCGGCTCACCGTCGCCGAGCGCGAGGCAGCCGACGCCAAGACCGACCTCGAGACGCTGAAG
		15719-15909	CGAAGAAGCTCCGCGACGCGCAGCAGAAGGTCACCGACAAGGAGGCCGCCGCGGCACTCGCGCAGACCAAGCTCAACGAGACCC TGAACAACCCGAAGGCCAAGGAGTCG-- GCTCGGCAGGCCGCCCGCGACCGCCTCACCATCGCGCAGCGCGAGGCCGCCGACGCGAAGACCGACCTTGAGACGCTCAAG
**TPA4-D2**	85	24402-24482	CGGCGGTGATGCCTGGGGCAGCAGCGGGGGTTCCGCCCGCCGTG---GT- CGCGGGGTCGGTGCTGCAGCCGCCGCCGATGACGG
		24087-24170	CGGCGCGGATGCCCGCAGCCGCCGAGGTGTATCCGCCGACGGTGCGGGTGCTCGTGG- CGGAGGTCCTGCCGCCGCCGATGACGG
**TPA4-D3**	66	5650-5715	TGCAGAAGCAGCTCGACGACGCCGTCGCCGCGCAGAAGAAGTCCGACGAGGCCGCCGCGACGGCGA
		5557-5610	TGCAGAAGCAGGTCGACG-CGC--TCACC---------AAGGCAGCCGAGGACGCCGCGAAGGCGA
**TPA4-D4**	63	15637-15695	GGCAGCGGCTCGGGCTCGGGCTCG-GGCAGTGG--CGGCGGTAGCGGGTCCGG-TTCGTCGGC
		15541-15603	GGCAGCAGCTCGGGCTCGGGGTCGTCGAAGTCGTACGGCGGCGGCGGGTTCGGCAGCGTCGGC
**TPA4-D5**	51	53573-53623	AGCGGATCGCCGGCACCGTCACCGCCGTCGCGCTCAACGCCGACGGCAGCC
		12800-12849	AGCGAATCACCGCGGCCACCACCGCCGTCGCCGCGAACGCCG-CGGCGGCC
**TPA4-D6**	49	44595-44642	GGACCGCACGAG-GCGCAGGTCGACGCCCTCGTCAACAAGTTCAGCGGC
		15192-15237	GGACTGCTCGGGCGCGCAGG-CGGCGA--TCGTCAACAAGATCACCGGC
**TPA4-D7**	47	42031-42074	ATCGGCGACCGGATGCCGCGCATC-ACCGGC--GCCATGCCGGCGGC
		16426-16472	ATCGGCGACTTCATGAAGCCGATCGACCGTCCGGCCATGCCGGCGGC
**TPA4-D8**	47	43938-43983	CGAGGTGCCCGATCAGGTGGAGCT-GCTCCGCCTGGCGCAGGCCGAG
		20066-20111	CGAGGTGCCCGACCCGGACGAGCTCGACCTGCCTGAC-CCGGCCGAG
**TPA4-D9**	45	13067-13111	TCGGTCAGCGTGTGCCGGTCATCGCGTCGATGCAGCAGGCGTTCG
		1709-1753	TCGCTCCGCGCGAGAAGGTCGACGCGACGGTGCGGCAGGCGTTCG
**TPA4-D10**	44	37853-37896	GCGAGGAGGTGTCCGCGACGGCGGGCCGGACCTCGCAGATCGAG
		13508-13548	GCGACGAGGG--CCGCGAAGGCAC-CCGGAACGCGCAGATCGAG

Phage-Repeat number	Size (bp)	Coordinates	Sequence alignment
**TPA4-D11**	43	38973-39012	CCACCCCACCACC---AGGAGACCCGCGATGGCCGACTACGAG
		36901-36940	CCACCCCACCACCGAAAGGA-ACC--CCATGTCCGACAACGAG
**TPA4-D12**	42	40327-40368	GCCCTGCTCCGCGGCGAACGCGACGAAGCCCGCCGGCAGCTC
		29231-29271	GCCGTGCCGCGCGCCGACCG-GTCGATGCCCGCCGGCACCTC
**TPA4-D13**	41	43785-43825	GCACAAGGCCGCGCTCGTGCAGGCGATCGACGGGAAGGTCA
		9507-9547	GCACTACGCCTACCTCGTCGAGACCTTCGACGGGAAGGTCA
**TPA4-D14**	40	43663-43699	CTGACCGAGA---TCCGCGCCGACCTCGACGACCTGCACG
		40791-40830	CTGCCCGAGAAGTTCCGCGCCGACCACGACGAGCGGTACG
**TPA4-D15**	39	48273-48310	CATCGCCGAGCTCCGCG-GCGGCGGCTCGACGGCGCGCC
		35757-35794	CATCACCGAGCTCCGCGAGCAGCGCGACGA-GGCCCGCC
**TPA4-D16**	38	54100-54137	CACCGTCCGCCTCGTCGACTTCGCCGGCCGCCTCCTCC
		53382-53419	CACCGTCCGCCTCATCGCCCCCGACGGCCGCCCCATCC
**TPA4-D17**	38	52704-52741	CACCGACCAGACCGACCGCGCCATCGCCGCGATCGACG
		1965-2002	CACCGAGCAGGCCGAGCTCACCGCCCGCGCGATCGACG
**TPA4-D18**	38	45281-45318	TGACCGCCAGCGTCACCAGCCTCATCGCGCACCGCGCC
		3745-3782	TGATCGACGGCCTCGCCCACCTCATCGCGCAGCTCGCC
**TPA4-D19**	38	47672-47709	CGCAGCACGGCGTCGTGCGCGCGATGCAGGCGTTCAAG
		29355-29392	CGAAGGACGCCGCCCCGGGCGCGGTGCAGGCGTTCGAG
**TPA4-D20**	37	46738-46771	CCGCGCCCGCCGCGTCC---TCCTCGGCGAGACCACC
		15000-15036	CCGCGCCCGTTCCGTCGCGATCCTCGGCGAGACCGCC
**TPA4-D21**	37	41653-41689	GCCATCGCCGCCGCGCAGGCGCACGGGATCCTCGCGC
		30287-30320	GCCATCGCCGCCGCGATCGCTCTCG---TCCTCGCGC
**TPA4-D22**	37	54589-54623	ATCATCCAGGCCGCCAAGTACCTCCGCACC--CGCCG
		16063-16099	ATGATCCAGGCCGCCGAGGACCGGCTCACCGTCGCCG
**TPA4-D23**	36	19434-19465	TCGTCGGCGGCGCCGC-GGTGGCC---GCCTGACCC
		16382-16417	TCGTCGGCGGCGCGGCGGGCGGCCTCGGCCTGACCC
**TPA4-D24**	36	21380-21415	CCGTCGTCGAGTGGCGCGACATGGGCGAGCTCGACC
		20059-20094	CCGCCGGCGAGGTGCCCGACCCGGACGAGCTCGACC
**TPA4-D25**	35	16810-16843	GGAACCG-CGGCGTCGTCCTCGACGAGGGACTCGA
		2466-2500	GGCACCGTCGTCGACGTCCTCGACGAGCGTCTCGA
**TPA4-D26**	35	8588-8622	CGCGCCGCCGCGCACGAGGCGAAGACCCACGCGCT

Phage-Repeat number	Size (bp)	Coordinates	Sequence alignment
		22827-22861	CGCGCCGCCGCCGTCGAGGCGCGGATCCGCGCGCT
**TPA4-D27**	35	41640-41674	GGCGCCTGACCCGGCCATCGCCGCCGCGCAGGCGC
		39297-39331	GGCGCTGGACGCGGAGATCGCCGCCGCGGCGGCGC
**TPA4-D28**	35	51511-51545	CGCCGAGGGGCCGGCCGGTGCACGGGAGCTGCGGA
		49933-49967	CGGCGAGGCGCTCGCCGCCGAACGGGAGCTGCAGA
**TPA4-D29**	33	22478-22509	CCTCGCCGACGGCACCGTCCGCTTCGAC-CGGA
		17420-17452	CCGCGCCGACGGCATGGTCCTCGTCGACACGGA
**TPA4-D30**	33	14904-14936	CCGCCGCGCCGACATCTACGCGGGCCGCGGCGC
		11538-11570	CCGCCGCGCCGTCAACTCGGCAGAGCGCGGCGC
**TPA4-D31**	32	50766-50797	TCGACGCCCTCGCCGCCCTGTCCGCCCGCTCG
		21874-21905	TCGACGCCCTCGCCAGCCTGTTCGGCGGCGCG
**TPA4-D32**	32	42155-42186	TGTTCGCGCTCGCCGACGAACTCGGTGTCAAG
		36724-36755	TGATCGTGCTCGCCGACGTTCTCGGCGTCGAG
**TPA4-D33**	32	2932-2963	TCACCCGCGAGATCGCCTACCTGACCGACGCG
		52336-52367	TCGCCCGCGCCATCGCCTACCTCGCCGACCCG
**TPA4-D34**	31	34598-34626	CCGCGCCCGCCGCGGCGACGC--CCGGCCTC
		6666-6696	CCGCGCGCGGGGCGGCGACGCCGCCGGCCTC
**TPA4-D35**	31	45367-45397	GCCGGCACGGACACCGTCGGCGACGTCGTGC
		4132-4157	GCCGGCACG-----CGTCGGCGACGGCGTGC
**TPA4-D36**	31	50747-50777	CTCGTCAGCATCAACGAGGTCGACGCCCTCG
		8390-8420	CTCCTCAACAGCGACGGCGTCGACGCCCTCG
**TPA4-D37**	31	48345-48375	CGGCAACCACCACGCCCTCGTCATGCGGAAC
		47514-47544	CGGCTACCGGCACCGCCTCGTCATGCTGAAC
**TPA4-D38**	30	51887-51915	CAGAGG-TTCAGAGGGCGACCCTCGGAACC
		51712-51741	CAGAGGGTTCCGAGGGCGACCCTCGGAACC
**TPA4-D39**	30	53964-53993	ATGCACGGAACCGAGGAGACCCGATGACCG
		53672-53700	ATGC-CGAACCCGAGGAGACCCGATGACCG
**TPA4-D40**	30	37039-37068	CGCCGAGTGCGTCGACGCGGCCCTCGCCAC
		7059-7088	CGCCGACGGTGTGAACGCGGCCCTCGCCAC
**TPA4-D41**	29	54870-54898	CCGCCTGTTCACCGCAGCCGAACTCGTCG
		45733-45761	CCGCCTGTTCACCCCGGACGAACTCGTCG
**TPA4-D42**	29	37560-37588	CGTGATCTACGACGAGCTCGGCGCCGACG

Phage-Repeat number	Size (bp)	Coordinates	Sequence alignment
		14355-14383	CGCGATCACCGACGAGCTCGGCCCGGACG
**TPA4-D43**	29	44614-44642	TCGACGCCCTCGTCAACAAGTTCAGCGGC
		21874-21902	TCGACGCCCTCGCCAGCCTGTTCGGCGGC
**TPA4-D44**	29	48083-48111	GCCGCGACGGCAAGCAGGCGCAGGCCGCG
		29016-29044	GCCGCGACGTCGCGGCGGCGCAGGCCGCG
**TPA4-D45**	29	35954-35982	CCGAGAAGGACGCCGCCTACCGCACGCGG
		29352-29378	CCGCGAAGGACGCCGCC--CCGGGCGCGG
**TPA4-D46**	29	43702-43728	CCGAA--CGACGACACCGCCGACCAGGTC
		36124-36152	CCGAAGCCGACCGCATCGCCGACCAGGTC
**TPA4-D47**	28	24628-24655	GCCCGGTCTGCCGTACACGCTGCCGTTC
		24238-24265	GCACGGGCTGCCGTACACGCTGCCGTTC
**TPA4-D48**	28	7774-7801	TCCGACGAGATCCCCGACCCCGCGGTCA
		1432-1458	TCCGACGAGATCCTCGA-CCCGCGGACA
**TPA4-D49**	28	4350-4377	ATCGCGAACGCGATGGACAAGGCCGAGG
		958-985	ATCGCGAACGCGATGTTCAGTGGCGAGG
**TPA4-D50**	28	35004-35031	GTCGTCGCCGACGACCGCAGGCGCGTCG
		5006-5033	GTGGTCGCCGACGACAGCTGGGACGTCG
**TPA4-D51**	28	43704-43731	GAACGACGACACCGCCGACCAGGTCTTC
		23659-23686	GAACGACGACAAGGGCAACCAGGTCATC
**TPA4-D52**	27	54549-54574	CACGACCGCCCGGAAGC-TCCCGCACC
		39619-39645	CATGACCGCCCGGAAGCGGCCCGCGCC
**TPA4-D53**	26	55013-55038	GCGGCGACGTCCTCGACGCCCTCGCC
		21862-21887	GCCGCGGCGTGCTCGACGCCCTCGCC
**TPA4-D54**	26	47314-47339	CGCGCCCTGCGCGGCGACGCCCAGCC
		34599-34624	CGCGCCCGCCGCGGCGACGCCCGGCC
**TPA4-D55**	26	22403-22428	GCAGCAGCTCACCGGCGAGTGGACCG
		7839-7864	GCAGCAGCTCAACGGCGACATGACCG
**TPA4-D56**	26	27891-27916	CGACGGCGCGTACGCCGCCGCGCGCG
		8578-8603	CGACGGGGCGCGCGCCGCCGCGCACG
**TPA4-D57**	26	35422-35447	GACGTGGCGTGAGGCGCCGCGCACGA
		8579-8604	GACGGGGCGCGCGCCGCCGCGCACGA

Phage-Repeat number	Size (bp)	Coordinates	Sequence alignment
**TPA4-D58**	26	42954-42979	CTTCACCGGCGCCCGCACGCCCGCCG
		34309-34334	CTTCACCGGCGCCCACCTGACCGCCG
**TPA4-D59**	26	45530-45555	CATCGCCGCAGCCGTCGCCGCGGTGG
		34560-34585	CACCGTCGCGGCCGTCGCCGCAGTGG
**TPA4-D60**	26	40280-40305	GGATCGCGCAGCTCCGCGCCACCGCC
		40008-40033	GGATCGCGCAGCTCTGGACCACCACC
**TPA4-D61**	25	38560-38583	CCTCGCAGGCGGCGAACGC-GCTCC
		7752-7776	CCTCGCAGGCGCCGAACGCGGCTCC
**TPA4-D62**	25	34591-34613	TGCAGC--GCCGCGCCCGCCGCGGC
		38623-38647	TGCAGCAGGCCCCGCCCGCCGCGGC
**TPA4-D63**	25	9438-9462	CAACGTCACCCCGGCGACCGCGGCA
		1675-1699	CAGCGGCCCCACGGCGACCGCGGCA
**TPA4-D64**	25	35216-35240	CGCAGAGTCGGTGGCGGCATGAGCG
		23589-23612	CGCAGTGGCG-TGGCGGCATGAGCG
**TPA4-D65**	25	30158-30182	GCGGCACCGCCGCGGCGGCACCGAG
		28110-28134	GCGGCACCGCCTCGGCTGGGCCGAG
**TPA4-D66**	25	51502-51526	CGCCGTGCTCGCCGAGGGGCCGGCC
		30002-30026	CGCCGTGCTCGCCGCCGGCCAGGCC
**TPA4-D67**	25	44115-44139	CGCCGAGGCGCGCCGGCAGCTCGCC
		40347-40371	CGACGAAGCCCGCCGGCAGCTCACC
**TPA4-D68**	25	53578-53602	ATCGCCGGCACCGTCACCGCCGTCG
		45364-45388	ATCGCCGGCACGGACACCGTCGGCG
**TPA4-D69**	24	42774-42797	GGCAGATCACCCTCACCGAGACCA
		40361-40384	GGCAGCTCACCATCACCGAGACCA
**TPA4-D70**	24	13848-13871	CAAGACCGCCCTCGACGCGCTGAA
		13575-13598	CAAGACCGCCCTCGACAACCTGAA
**TPA4-D71**	24	21283-21306	CCGATCCTCCGCAGCGAGTGGACC
		16626-16649	CCGATGAGCCGCAGCGAGTGGACC
**TPA4-D72**	24	54856-54879	GACGCCCTCGTCGACCGCCTGTTC
		17382-17405	GACGGCCTCGTCGACCGCATGGTC
**TPA4-D73**	24	18479-18502	GAGGACACCGGCGTCGGCCTGATC

Phage-Repeat number	Size (bp)	Coordinates	Sequence alignment
		17933-17956	GACGACACCGGCGTCGGCACGATC
**TPA4-D74**	24	37043-37066	GAGTGCGTCGACGCGGCCCTCGCC
		36034-36057	GAGTGCGTCGACGCCTACCTCGCC
**TPA4-D75**	24	52709-52732	ACCAGACCGACCGCGCCATCGCCG
		49041-49064	ACCAGACCGACAGCCCCACCGCCG
**TPA4-D76**	23	14860-14882	GTCCCGATGCTCGCGGGCGGCCT
		13252-13274	GTCGCGATGGTCGCGGGCGGCCT
**TPA4-D77**	23	14717-14739	CGAAGGCCGCGGTGGACGCGATC
		193-215	CGAAGGCCGCGGTCCGCGCGATC
**TPA4-D78**	23	20039-20061	GCTCCCGGAGCCGATCACCGCCG
		2429-2451	GCTCCCGGAGCTGATCCGCGCCG
**TPA4-D79**	23	22990-23012	CCGTGTGGCAGCAGTTCGGCGGC
		22396-22418	CCGTGTGGCAGCAGCTCACCGGC
**TPA4-D80**	23	38460-38482	CGGCTGACCATCGCCGTCGTGGC
		28796-28818	CGGCTGACCATCGGCATCGAGGC
**TPA4-D81**	23	45933-45955	CCACCGACCCCGCGGTGCTCGAC
		42086-42108	CCACCGACCCCGAGATCCTCGAC
**TPA4-D82**	23	55318-55340	TCGCCGACCTCGGCGACTGCGGC
		43128-43150	TCCCCGACATCTGCGACTGCGGC
**TPA4-D83**	23	52509-52531	TCGTCGCCCAGCGCACCCGCCTC
		49878-49900	TCGTCGCCCAGGCCACCCGCATC
**TPA4-D84**	22	6068-6089	CACCGAGACGCCGAAGTCGGCG
		4937-4958	CACCTCGACGCCGAAGTCGGCG
**TPA4-D85**	22	23554-23575	CCGCGTCCTGAAGGAGCTCGCG
		12066-12087	CCGCGGGCTGAAGGAGCTCGCG
**TPA4-D86**	22	49325-49346	GGCACCGGCAAGTCCGGCGACG
		16150-16171	GGCACCACCAAGTCCGGCGACG
**TPA4-D87**	22	49201-49222	CGCCCTCGCCGACGGCGACGTC
		22475-22496	CGCCCTCGCCGACGGCACCGTC
**TPA4-D88**	22	39205-39226	GGCGTCGTCGCCCGCCACCCCG
		28610-28631	GGCGTCGTCGCCCACCACACCG

Phage-Repeat number	Size (bp)	Coordinates	Sequence alignment
**TPA4-D89**	22	44240-44261	CGGCGCGGTCCGCGTCGCCGTC
		37123-37144	CGGCGCGGTCCGCCTCGCCATC
**TPA4-D90**	21	12412-12432	ATCGACGGCCTCGGCCACCTC
		3747-3767	ATCGACGGCCTCGCCCACCTC
**TPA4-D91**	21	28895-28915	CGCGGCGTCGCGGCGATCCTC
		21271-21291	CGCGGCGTCGCGCCGATCCTC
**TPA4-D92**	21	55016-55036	GCGACGTCCTCGACGCCCTCG
		8400-8420	GCGACGGCGTCGACGCCCTCG
**TPA4-D93**	21	23132-23152	CGTCGGGGCGCGCGCCGACGC
		8578-8598	CGACGGGGCGCGCGCCGCCGC
**TPA4-D94**	21	54491-54511	TCGCCTCCGACGTCGCCACCG
		13046-13066	TCGGCTCCGCCGTCGCCACCG
**TPA4-D95**	21	22947-22967	GGCGGCGGCGCCGGCACCCTC
		14926-14946	GGCCGCGGCGCCGGCACCGTC
**TPA4-D96**	21	51153-51173	CCGCCGCCGGTGACGAGATCG
		23260-23280	CCGCCGCCGGTGACGTGTTCG
**TPA4-D97**	21	54423-54443	CGGGCCCCGCGGCCGGCGCGC
		27504-27524	CGTGCCCCGCGGCCAGCGCGC
**TPA4-D98**	21	53046-53066	CGACCACGACGACGCCCGCGA
		50263-50283	CGACCACGACGACGACCACGA
**TPA4-D99**	20	37577-37596	TCGGCGCCGACGCGACGACC
		6325-6344	TCGCCGCCGACGCGACGACC
**TPA4-D100**	20	36204-36223	GAGCTCGGCCCGTCCGAGGT
		21406-21425	GAGCTCGACCCGTCCGAGGT
**TPA4-D101**	20	29484-29503	GCGGCCCGCTCGGCTACCCG
		29310-29329	GCGGCCCGCTCGGCTTCCCG
**TPA4-D102**	20	50026-50044	GGTCGA-GGCCGCGGTCCGC
		190-209	GGTCGAAGGCCGCGGTCCGC
**TPA4-D103**	20	34359-34378	TCGTCCTCGTCCGCGCGTAC
		3211-3230	TCGACCTTGTCCGCGCGTAC
**TPA4-D104**	20	8691-8710	CGCCGAGCTCGGCGTCCCCG

Phage-Repeat number	Size (bp)	Coordinates	Sequence alignment
		3782-3801	CGCCCAGCTCGGCGTCCGCG
**TPA4-D105**	20	16940-16959	CCAGCTCGGCGTCCACATCG
		3785-3804	CCAGCTCGGCGTCCGCGTCG
**TPA4-D106**	20	20113-20132	TCGACGACCTCATCCCGTGG
		6899-6918	TCGACGACCTCATCACCTGG
**TPA4-D107**	20	29700-29719	GGCGCGCATCGCCGCGCACG
		8584-8603	GGCGCGCGCCGCCGCGCACG
**TPA4-D108**	20	41211-41230	GCACCGCGCTCGGCGAGACC
		11197-11216	GCACCGCGCTCCGCGAGGCC
**TPA4-D109**	20	29380-29399	GCAGGCGTTCGAGGGCGGCG
		13101-13120	GCAGGCGTTCGTGCGCGGCG
**TPA4-D110**	20	46297-46316	GACACCGTCGCCGGGCAGCT
		13651-13670	GACACCGTCGCCGCGAAGCT
**TPA4-D111**	20	52212-52231	AAGGCCCGCGCGCACACCGG
		14176-14195	AAGGCCCGCGCGCAGATCGG
**TPA4-D112**	20	53580-53599	CGCCGGCACCGTCACCGCCG
		14934-14953	CGCCGGCACCGTCTTCGCCG
**TPA4-D113**	20	27912-27931	GCGCGGCCGGTTCGTCGGCC
		15677-15696	GCGGGTCCGGTTCGTCGGCC
**TPA4-D114**	20	55013-55032	GCGGCGACGTCCTCGACGCC
		22768-22787	GCGACGACGTCCTCGGCGCC
**TPA4-D115**	20	36966-36985	AGTTCACCGACTACGCGGTC
		26524-26543	AGATCGCCGACTACGCGGTC
**TPA4-D116**	20	34174-34193	TCGGCCTCGGCAAAAGCCAG
		27926-27945	TCGGCCTCGGCTACAGCCAG
**TPA4-D117**	20	29401-29420	GCTGTACCGCCGCGACGGCG
		29008-29027	GCTGTTCCGCCGCGACGTCG
**TPA4-D118**	20	43674-43693	CCGCGCCGACCTCGACGACC
		43565-43584	CCGCCGCGACCTCGACGACC
**TPA4-D119**	20	54515-54534	TCGCCGGCACCCTCAACGCC
		53579-53598	TCGCCGGCACCGTCACCGCC

Phage-Repeat number	Size (bp)	Coordinates	Sequence alignment
**TPA4-D120**	19	12486-12504	GGGCAGCAACGGCCTGCTC
		4700-4718	GGGCAGCAACGTCCTGCTC
**TPA4-D121**	19	44613-44631	GTCGACGCCCTCGTCAACA
		8408-8426	GTCGACGCCCTCGTGAACA
**TPA4-D122**	19	42873-42891	GAAGATCGGCAACGTCGTC
		14388-14406	GAAGACCGGCAACGTCGTC
**TPA4-D123**	19	37315-37333	GAGCCGATCACCGGCGCCG
		20046-20064	GAGCCGATCACCGCCGCCG
**TPA4-D124**	19	45318-45336	CCTCACCCAAGCCGACAAG
		20416-20434	CCTCACCGAAGCCGACAAG
**TPA4-D125**	19	30162-30180	CACCGCCGCGGCGGCACCG
		29291-29309	CAGCGCCGCGGCGGCACCG
**TPA4-D126**	19	32243-32261	GGCCTCGCCGATGCGGATG
		31928-31946	GGCCTCGCCGATGCGGGTG
**TPA4-D127**	19	43571-43589	CGACCTCGACGACCCCGCG
		35811-35829	CGACCTCGACGACCCTGCG
**TPA4-D128**	19	50085-50103	TCGCGGAGGAGCTCGGCGA
		42590-42608	TCGCGGAGGAGCACGGCGA
**TPA4-D129**	19	45051-45069	ACGTCAACGCGATCGGCGA
		44293-44311	ACGGCAACGCGATCGGCGA
**TPA4-D130**	19	53852-53870	CGAGCAGCGCGCCCCGGGC
		53013-53031	CGAGCAGCGCGCCCGGGGC
**TPA4-D131**	18	29407-29424	CCGCCGCGACGGCGACCC
		5701-5718	CCGCCGCGACGGCGACCC
**TPA4-D132**	18	17234-17251	GCGGATGCTCACCCGCGA
		2882-2899	GCGGATGCTCAACCGCGA
**TPA4-D133**	18	23340-23357	GGCGCGGTGATCCTCGAC
		4865-4882	GGCGCAGTGATCCTCGAC
**TPA4-D134**	18	15874-15891	GCCGCCGACGCGAAGACC
		6327-6344	GCCGCCGACGCGACGACC
**TPA4-D135**	18	23026-23043	GCATCGCCCGGTACACCG

Phage-Repeat number	Size (bp)	Coordinates	Sequence alignment
		8804-8821	GCATCGCCCGGCACACCG
**TPA4-D136**	18	28217-28234	GCCTCGCCGACCCGATCG
		9584-9601	GCCTCGGCGACCCGATCG
**TPA4-D137**	18	49670-49687	GGCGCCCCGGTCACCGTC
		10355-10372	GGCGCCGCGGTCACCGTC
**TPA4-D138**	18	19849-19866	GCGGCCCGGGTGGCGAGC
		15350-15367	GCGGCCCGGGTGGCGGGC
**TPA4-D139**	18	37581-37598	CGCCGACGCGACGACCGA
		15876-15893	CGCCGACGCGAAGACCGA
**TPA4-D140**	18	55013-55030	GCGGCGACGTCCTCGACG
		16816-16833	GCGGCGTCGTCCTCGACG
**TPA4-D141**	18	27573-27590	TCGATCACCGCGCACGCC
		20540-20557	TCGATCACCGCGTACGCC
**TPA4-D142**	18	40750-40767	TCACCGCGTACGTCGAGC
		20544-20561	TCACCGCGTACGCCGAGC
**TPA4-D143**	18	42102-42119	CCTCGACGGCATCGACGA
		22610-22627	CCTCGACGGCATCGTCGA
**TPA4-D144**	18	50440-50457	CCCGTGGGCCGTCCTCGG
		25065-25082	CCGGTGGGCCGTCCTCGG
**TPA4-D145**	18	53769-53786	CCGATCGCCCGCGGCGAC
		34741-34758	CCGATCACCCGCGGCGAC
**TPA4-D146**	18	50931-50948	CCCGCGCCGGTGCGCTCC
		39911-39928	CCCGTGCCGGTGCGCTCC
**TPA4-D147**	18	52403-52420	CCCCACCCACCAGGAGAA
		41950-41967	CCCCAACCACCAGGAGAA
**TPA4-D148**	18	54918-54935	CGGCCAGCGCATCACCGC
		45521-45538	CGGCCAGCGCATCGCCGC
**TPA4-D149**	18	52762-52779	ATCGACGACATCGTCGAC
		49367-49384	ATCGACGACATCGCCGAC
**TPA4-D150**	17	20988-21004	GCCTCGGCGACCCGATC
		9584-9600	GCCTCGGCGACCCGATC

Phage-Repeat number	Size (bp)	Coordinates	Sequence alignment
**TPA4-D151**	17	50160-50176	TCGGCCGGCCCGCCGCG
		21452-21468	TCGGCCGGCCCGCCGCG
**TPA4-D152**	16	40284-40299	CGCGCAGCTCCGCGCC
		2318-2333	CGCGCAGCTCCGCGCC
**TPA4-D153**	16	45010-45025	GACCGACCTCGAGGAG
		7614-7629	GACCGACCTCGAGGAG
**TPA4-D154**	16	38735-38750	GCAGGCCGCGCTGCTC
		11796-11811	GCAGGCCGCGCTGCTC
**TPA4-D155**	16	25110-25125	CCGCGTCGGCGGCGAC
		12165-12180	CCGCGTCGGCGGCGAC
**TPA4-D156**	16	23171-23186	GCGCGTGCGGATCGGC
		17117-17132	GCGCGTGCGGATCGGC
**TPA4-D157**	16	32032-32047	CGCGGGCGCGCAGCTG
		22304-22319	CGCGGGCGCGCAGCTG
**TPA4-D158**	16	36772-36787	CCGCCGCCGTCGAGGC
		22831-22846	CCGCCGCCGTCGAGGC
**TPA4-D159**	16	37270-37285	AACCCCATGTCCGACA
		36920-36935	AACCCCATGTCCGACA
**TPA4-D160**	16	54915-54930	CCTCGGCCAGCGCATC
		47835-47850	CCTCGGCCAGCGCATC
**TPA4-D161**	16	55025-55040	TCGACGCCCTCGCCGC
		50766-50781	TCGACGCCCTCGCCGC
**TPA4-D162**	15	11561-11575	AGCGCGGCGCGAAGG
		776-790	AGCGCGGCGCGAAGG
**TPA4-D163**	15	23900-23914	GCGGCGGCCGGGATG
		3246-3260	GCGGCGGCCGGGATG
**TPA4-D164**	15	35311-35325	CCGTCTCGAGAAGGC
		6750-6764	CCGTCTCGAGAAGGC
**TPA4-D165**	15	26050-26064	GCCAGGGCGACGACC
		10196-10210	GCCAGGGCGACGACC
**TPA4-D166**	15	18887-18901	CCGACGATCCTGAAC

Phage-Repeat number	Size (bp)	Coordinates	Sequence alignment
		12637-12651	CCGACGATCCTGAAC
**TPA4-D167**	15	42856-42870	GGTGACCGCATCGCC
		13432-13446	GGTGACCGCATCGCC
**TPA4-D168**	15	48225-48239	CGTCGCCGACGGCGC
		14588-14602	CGTCGCCGACGGCGC
**TPA4-D169**	15	43180-43194	CGAGATCCCCGCCGA
		17618-17632	CGAGATCCCCGCCGA
**TPA4-D170**	15	53478-53492	CGAGCTCGACCTGCC
		20084-20098	CGAGCTCGACCTGCC
**TPA4-D171**	15	36120-36134	CTCACCGAAGCCGAC
		20417-20431	CTCACCGAAGCCGAC
**TPA4-D172**	15	26736-26750	GGGACGGCGCGCACG
		22129-22143	GGGACGGCGCGCACG
**TPA4-D173**	15	50113-50127	CTACAGCGGCGGCGG
		22940-22954	CTACAGCGGCGGCGG
**TPA4-D174**	15	49142-49156	GGCGCCGGGAAGGTC
		26034-26048	GGCGCCGGGAAGGTC
**TPA4-D175**	15	52693-52707	CCGATCATGAGCACC
		40626-40640	CCGATCATGAGCACC
**TPA4-D176**	15	50763-50777	AGGTCGACGCCCTCG
		44611-44625	AGGTCGACGCCCTCG
**TPA4-D177**	15	54447-54461	GCGCGGCGCCACCGA
		45438-45452	GCGCGGCGCCACCGA
**TPA4-D178**	15	50770-50784	CGCCCTCGCCGCCCT
		46323-46337	CGCCCTCGCCGCCCT
**TPA4-D179**	15	52561-52575	CCCGCCGCGCCTGCG
		50168-50182	CCCGCCGCGCCTGCG
**TPA4-I1**	60	37008-37061	GCCGCGCGCTCGC---CGAGG--CGTCCCGC-GCCCTCGCCGAGTGCGTCGACGCGGCCC
		5524-5465	GCCGCGCGCTCGCTCTCGAGTGCCTTCTTGCCGCCCTCGCCGAGCGGCTCATCGCCGCCC
**TPA4-I2**	52	42939-42989	CGGCGACTACGGCATCTTC-ACCGGCGCCCGCACGCCCGCCGGCACCTACCC
		10361-10313	CGGCGCCCACGGCATCTTCGACCGCCGCGC---CGGCCGCCCTCACCTACCC

Phage-Repeat number	Size (bp)	Coordinates	Sequence alignment
**TPA4-I3**	51	15634-15684	CGCGGCAGCGGCTCGGGCTCGGGCTCGGGCAGTGGCGGCGGTAGCGGGTCC
		38675-38630	CGCGGCAGCGGCCGGGGCCTGGGC---GGGAGCCGCGGCGG--GCGGGGCC
**TPA4-I4**	41	24444-24484	TGGTCGCGGGGTCGGTGCTGCAGCCGCCGCCGATGACGGTG
		9208-9168	TGGTGGCGAGGTCGTACCGCCAGATGCCACCGATGACGGTG
**TPA4-I5**	40	29921-29960	CGCCCTCGCCACCGCGGTCCTCGGACCCGTGATCTCCGCG
		14268-14229	CGCCTCCGCCACCGCGGCGAGCTGCCGCGTGGTCTCCGCG
**TPA4-I6**	36	51889-51924	GAGGTTCAGAGGGCGACCCTCGGAACCCTCGGAACC
		51743-51708	GAGGTTCCGAGGGTCGCCCTCGGAACCCTCTGAACC
**TPA4-I7**	36	24150-24185	TCCTGCCGCCGCCGATGACGGCGGCGGCGGACGTGC
		19573-19539	TCTTGCCGCCCTCGATGACGGC-GTGGCGTACGTGC
**TPA4-I8**	36	41093-41126	CGCGCTGGACGACGCCGCGGCGGCG--CTCCGCATC
		39339-39304	CGCCCTGCGCGCCGCCGCGGCGGCGATCTCCGCGTC
**TPA4-I9**	35	44992-45026	GACCGTCCCCGACCAGGCGACCGACCTCGAGGAGC
		2862-2828	GACCGTGCTCGTCGCGGCGCACGACCTCGAGGCGC
**TPA4-I10**	34	25057-25088	CGGCGGCGCCGGT--GGGCCGTCCTCGGCGCTCG
		12865-12833	CGGCGGCGCCGGTGCGGGCCG-CCGCGGCGTTCG
**TPA4-I11**	33	42886-42918	GTCGTCGGCGACGACTATGACCCCGGCGGCGTC
		6714-6682	GTCGACGCCGCCGAGCATGAGGCCGGCGGCGTC
**TPA4-I12**	32	53103-53134	CCCCGTCGCGGAGGATCGAGAGCGCCTCGCCG
		1042-1011	CCCCGTCGCGGAGGATCGTGCGCGTCTCGCCG
**TPA4-I13**	32	25491-25522	CGCCGAACCAGGACACCTGGCGGTACCGGTCG
		1780-1752	CGCCGAACCA---CACCACGCGGTACCGGTCG
**TPA4-I14**	32	53001-53032	CGCGCACGAGCGCGAGCAGCGCGCCCGGGGCG
		29396-29365	CGCCCTCGAACGCCTGCACCGCGCCCGGGGCG
**TPA4-I15**	31	41862-41892	ACGCGCTCGAGCTCCTCGCCGCGCTGGCCGC
		27542-27512	ACGCGCGCGTGCCCCGCGGCGCGCTGGCCGC
**TPA4-I16**	31	34722-34752	CCTCACCGTCATCCAGGGGCCGATCACCCGC
		32591-32561	CCCCATCGGCACCCACGGGACGATCACCCGC
**TPA4-I17**	30	25877-25905	CGCCGCCGACGCGAACGACGTCGC-GAACG
		12178-12149	CGCCGCCGACGCGGAGGATCTCGCGGAACG
**TPA4-I18**	30	52015-52044	CCACCGCCCGCCCCCTCGCGAAGCGCGCCG

Phage-Repeat number	Size (bp)	Coordinates	Sequence alignment
		39304-39275	CCAGCGCCCGCCCCCACGGCACGCGGGCCG
**TPA4-I19**	28	38393-38420	CTGGATGACCGGCGCGCTCGGCGACGCG
		24382-24355	CTGCACGACCGGCGCGCGCAGCGCCGCG
**TPA4-I20**	28	30268-30295	CGCGGCGGCGATCATCGCTGCCATCGCC
		39324-39297	CGCGGCGGCGATCTCCGCGTCCAGCGCC
**TPA4-I21**	27	19762-19788	ACCTCGTCGAACCCGACGCCGACGACC
		5052-5026	ACCTCGGCGTACACGACGCCGACGTCC
**TPA4-I22**	27	54742-54768	GCCTGCCCCATCTGCAAGACCGACCTG
		23703-23678	GCCTGCCCCATCTGCACGA-TGACCTG
**TPA4-I23**	27	35964-35990	CGCCGCCTACCGCACGCGGGCCGAGGA
		39296-39271	CGCCCCC-ACGGCACGCGGGCCGCGGA
**TPA4-I24**	27	35530-35556	CCGGTACACGGGCGGCGCGAAGTGACC
		39677-39652	CCGGGACGCGGGCGGCGCGGA-TGACC
**TPA4-I25**	26	54695-54720	GCGCCGTCCGCATCGTCGACCGCCGC
		10359-10334	GCGCCCACGGCATCTTCGACCGCCGC
**TPA4-I26**	26	52480-52505	GACGAATCACGAGCGCGAGCATCTGC
		24601-24577	GACGA-TCAGAAGCGCGAGCATCTGC
**TPA4-I27**	25	43565-43587	CCGCCG--CGACCTCGACGACCCCG
		15581-15557	CCGCCGTACGACTTCGACGACCCCG
**TPA4-I28**	25	44098-44122	GGGCGCTGGTCCGGGCACGCCGAGG
		33934-33912	GGGCGC--GTGCGGGCACGCCGAGG
**TPA4-I29**	25	48395-48418	ACGGCGCCGAGCACGTC-ACGCCGG
		22789-22765	ACGGCGCCGAGGACGTCGTCGCCGG
**TPA4-I30**	25	54679-54703	CTCCACGCCATCACCCGCGCCGTCC
		32230-32206	CTCGACGCGTTCATCCGCGCCGTCC
**TPA4-I31**	25	42894-42918	CGACGACTATGACCCCGGCGGCGTC
		34152-34128	CGTCGACGGTGACACCGGCGGCGTC
**TPA4-I32**	24	31604-31627	GCCATCGGTGACGACGACGGTGCG
		6534-6511	GCCGTCGGTGCCGACGACGGTGCG
**TPA4-I33**	24	50098-50121	CGGCGACCACCAGCACTACAGCGG
		9999-9977	CGGCCACCACCAGCACTAC-GCGG

Phage-Repeat number	Size (bp)	Coordinates	Sequence alignment
**TPA4-I34**	24	53568-53591	GCGCGAGCGGATCGCCGGCACCGT
		31853-31830	GCGCGTGACGATCGCCGGCACCGT
**TPA4-I35**	24	47617-47640	GAAGGCGACCGGGAGCCGGACCTC
		41527-41504	GATGGCGACCGGGATCCGGGCCTC
**TPA4-I36**	23	29822-29844	CGCGGACCTCCGCGCCGCGCTCT
		11581-11559	CGCGGACCTTCGCGCCGCGCTCT
**TPA4-I37**	23	50137-50159	CCTCGAGGCGCTGCGCGAGCTCA
		5646-5624	CCTCGAGGCGCTTCTCGAGTTCA
**TPA4-I38**	23	53421-53443	GCTCGCCCGCCACGTCCGCGCGC
		11782-11760	GCGCGCCCGCCACGCCCGCGAGC
**TPA4-I39**	23	42069-42091	GGCGGCGTGGTGGCTGACCACCG
		27079-27057	GGCGGCGCGGATGCTGACCACCG
**TPA4-I40**	23	55008-55030	CCGCTGCGGCGACGTCCTCGACG
		34585-34563	CCACTGCGGCGACGGCCGCGACG
**TPA4-I41**	22	45481-45502	CCGCCCACGACAGCGGCACCGC
		24791-24770	CCGCCCCGGACAGCGGCACCGC
**TPA4-I42**	22	31916-31937	GCGGATCGCGAGGGCCTCGCCG
		30038-30017	GCCGATCGCGAGGGCCTGGCCG
**TPA4-I43**	22	19727-19748	GCCGCGCGAGGTTCTGCTCACC
		7968-7949	GCCGCGCGAGGTTCT--TCACC
**TPA4-I44**	22	47755-47776	CGCTGCCCGCGCGTCGAGTGCC
		46636-46616	CGCTCCCC-CGCGTCGAGTGCC
**TPA4-I45**	21	48276-48296	CGCCGAGCTCCGCGGCGGCGG
		2331-2311	CGCGGAGCTGCGCGGCGGCGG
**TPA4-I46**	21	33300-33320	CGTCGGCCACGACTTCGGCGT
		4964-4944	CGTCGGCGCCGACTTCGGCGT
**TPA4-I47**	21	44884-44904	TATTCGTCGACGCCGACTTCG
		4968-4948	TACTCGTCGGCGCCGACTTCG
**TPA4-I48**	21	40491-40511	CATCGACGCCGCCGGGGACGT
		7500-7480	CATCGACGCCGCGAGGGACGT
**TPA4-I49**	21	51329-51349	GCGATGGCCGTGTCGAAGCTG

Phage-Repeat number	Size (bp)	Coordinates	Sequence alignment
		15614-15594	GCGATGGCCGTGCCGACGCTG
**TPA4-I50**	21	45376-45396	GACACCGTCGGCGACGTCGTG
		19191-19171	GACACCTTCGGCGACGTCTTG
**TPA4-I51**	21	37916-37936	TCAGCGCGCCGCCGTTCGAGG
		25787-25767	TCAGCGTGCCGCCGTTCGGGG
**TPA4-I52**	21	49890-49910	CCACCCGCATCGCCGCGGCCC
		31947-31927	CCACCCGCATCGGCGAGGCCC
**TPA4-I53**	21	52746-52766	CCTCACCGACGACCAGATCGA
		32069-32049	CCTCACCGACGACGAGCTCGA
**TPA4-I54**	21	45946-45966	GGTGCTCGACCTCGACGTCCA
		42421-42401	GGAGCTCGACCTCGAGGTCCA
**TPA4-I55**	21	48073-48093	CCGGTCGAGGGCCGCGACGGC
		46344-46324	CCGGTCGAGGGCGGCGAGGGC
**TPA4-I56**	20	37784-37803	GGGCGAGCTCGTCCGGGTCG
		20094-20075	GGTCGAGCTCGTCCGGGTCG
**TPA4-I57**	20	22258-22276	GGCTCGCCGGCCGC-GCGTC
		2656-2637	GGCTCGCCGGCCGCGGCGTC
**TPA4-I58**	20	31745-31764	GGCCTCGGCGGCGAAGTCGG
		10902-10883	GGACTCGACGGCGAAGTCGG
**TPA4-I59**	20	15832-15850	CGGC-AGGCCGCCCGCGACC
		13279-13260	CGGCGAGGCCGCCCGCGACC
**TPA4-I60**	20	43849-43868	GACGCCCTGCGCGCCGAGCG
		14520-14501	GACGGCCTGCGCGCCGGGCG
**TPA4-I61**	20	41559-41578	CGCCGAGCACGTCGCCGAGG
		17900-17881	CGCCGAGCACGGTGCCGAGG
**TPA4-I62**	20	52170-52189	GAACTCCTCGCCTTCCAAGA
		19596-19577	GAACTCCTCGCCATCCAGGA
**TPA4-I63**	20	45836-45855	GTCTGGCACCTCGCCAGCGG
		20078-20059	GTCGGGCACCTCGCCGGCGG
**TPA4-I64**	20	30710-30729	CGCACCGGACAGCGGCCCCG
		24790-24771	CGCCCCGGACAGCGGCACCG

Phage-Repeat number	Size (bp)	Coordinates	Sequence alignment
**TPA4-I65**	20	29062-29081	GCCGGGGCCGGTGCGCAACG
		28200-28181	GCCGGCGCCGGTGCGCAGCG
**TPA4-I66**	20	42231-42250	CGCGGAGATGCGCGCCGACC
		29715-29696	CGCGGCGATGCGCGCCGGCC
**TPA4-I67**	20	45076-45095	CGAGGACGACGCCGGCCACG
		33310-33291	CGTGGCCGACGCCGGCCACG
**TPA4-I68**	20	48392-48411	GCGACGGCGCCGAGCACGTC
		37589-37570	GCGTCGGCGCCGAGCTCGTC
**TPA4-I69**	20	34267-34286	CCGCGGACCTGCGCGCCGCC
		39343-39324	CCGCCGCCCTGCGCGCCGCC
**TPA4-I70**	19	38403-38421	GGCGCGCTCGGCGACGCGG
		3673-3655	GGCGCGCTCGGCGCCGCGG
**TPA4-I71**	19	50413-50431	CCGCGTCGCGCTGCTCGCG
		35788-35770	CCTCGTCGCGCTGCTCGCG
**TPA4-I72**	18	8776-8793	GTCCTCGGCGGCGGCAAG
		1512-1495	GTCCTCGGCGGCGGCCAG
**TPA4-I73**	18	24554-24571	GCGGCGACCGCGGCGCCG
		19458-19441	GCGGCCACCGCGGCGCCG
**TPA4-I74**	18	32080-32097	CGCCGCGGTCGACGCGTC
		24568-24551	CGCCGCGGTCGCCGCGTC
**TPA4-I75**	18	51118-51135	GCACGCCGGCCGCGGCGA
		25277-25260	GCACGCCGGCCGCGCCGA
**TPA4-I76**	18	44335-44352	ACTGCGACGACGGCCGCG
		34583-34566	ACTGCGGCGACGGCCGCG
**TPA4-I77**	18	42101-42118	TCCTCGACGGCATCGACG
		36796-36779	TCCTCGACGGCCTCGACG
**TPA4-I78**	18	47312-47329	AGCGCGCCCTGCGCGGCG
		40068-40051	AGCGCGACCTGCGCGGCG
**TPA4-I79**	18	51399-51416	CCCGGGAGCGCGGCGAGC
		51285-51268	CCCGCGAGCGCGGCGAGC
**TPA4-I80**	17	21372-21388	GGAGCTCACCGTCGTCG

Phage-Repeat number	Size (bp)	Coordinates	Sequence alignment
		7458-7442	GGAGCTCACCGTCGTCG
**TPA4-I81**	17	28933-28949	CGCGAGCCACGCGATCG
		12762-12746	CGCGAGCCACGCGATCG
**TPA4-I82**	17	50472-50488	GCACCCTCGCCGCGATC
		25944-25928	GCACCCTCGCCGCGATC
**TPA4-I83**	16	25111-25126	CGCGTCGGCGGCGACC
		6338-6323	CGCGTCGGCGGCGACC
**TPA4-I84**	16	47886-47901	CGCGCGGATCCGCGCC
		22859-22844	CGCGCGGATCCGCGCC
**TPA4-I85**	16	41077-41092	ACGAGCACCCGCACCG
		24141-24126	ACGAGCACCCGCACCG
**TPA4-I86**	16	50162-50177	GGCCGGCCCGCCGCGC
		30332-30317	GGCCGGCCCGCCGCGC
**TPA4-I87**	15	53441-53455	CGCACCAGCTGCAGC
		599-585	CGCACCAGCTGCAGC
**TPA4-I88**	15	32388-32402	TCGCGGGTGATCTCG
		2941-2927	TCGCGGGTGATCTCG
**TPA4-I89**	15	35883-35897	CCCGGCCGCCGCGGC
		3257-3243	CCCGGCCGCCGCGGC
**TPA4-I90**	15	5384-5398	GCCGGCGCTCGGCGC
		3357-3343	GCCGGCGCTCGGCGC
**TPA4-I91**	15	22164-22178	GGCGTCCTCGGCTGC
		5600-5586	GGCGTCCTCGGCTGC
**TPA4-I92**	15	19984-19998	GCCAGATGCCGGGGA
		6288-6274	GCCAGATGCCGGGGA
**TPA4-I93**	15	12166-12180	CGCGTCGGCGGCGAC
		6338-6324	CGCGTCGGCGGCGAC
**TPA4-I94**	15	49676-49690	CCGGTCACCGTCGCG
		9875-9861	CCGGTCACCGTCGCG
**TPA4-I95**	15	24554-24568	GCGGCGACCGCGGCG
		11732-11718	GCGGCGACCGCGGCG

Phage-Repeat number	Size (bp)	Coordinates	Sequence alignment
**TPA4-I96**	15	15834-15848	GCAGGCCGCCCGCGA
		14884-14870	GCAGGCCGCCCGCGA
**TPA4-I97**	15	37522-37536	TCGACGGTGTGGGCC
		20543-20529	TCGACGGTGTGGGCC
**TPA4-I98**	15	30318-30332	CGCGGCGGGCCGGCC
		21468-21454	CGCGGCGGGCCGGCC
**TPA4-I99**	15	39846-39860	CGACCGCCGAGCCCC
		23783-23769	CGACCGCCGAGCCCC
**TPA4-I100**	15	48757-48771	GCACCCGCACCGTCG
		24137-24123	GCACCCGCACCGTCG
**TPA4-I101**	15	42886-42900	GTCGTCGGCGACGAC
		35018-35004	GTCGTCGGCGACGAC
**TPA4-I102**	15	43440-43454	CGCGACCTGCGCGGC
		40066-40052	CGCGACCTGCGCGGC
**TPA4-I103**	15	41425-41439	CGACCTCGGCGCCGA
		40169-40155	CGACCTCGGCGCCGA
**GMA1-D1**	120	121-240	CCGCCGCCGTCATCGCCGACGGCCGCACCCGCCGCGCCGCCCTCGAACAACGCCTCATCGACGAAGCCAACAAGGCGTTGGACACCATGTGGGCGCCGCACGAGATAGGCGCATTCGGTG
		1-120	CCGCCGCCGTCATCGCCGACGGCCGCACCCGCCGCGCCGCCCTCGAACAACGCCTCATCGACGAAGCCAACAAGGCGTTGGACACCATGTGGGCGCCGCACGAGATAGGCGCATTCGGTG
**GMA1-D2**	54	4744-4791	CGACACCGACAAGTCG------ACGACAGCCGACACCGCGGCCGACAAGGGCGA
		2631-2684	CGACACCGACAAGACGATCTTCACGCCAGTCGGCGCAGCGTCAGATAAGGGCGA
**GMA1-D3**	39	20641-20679	ACGCCGGTCCGACTATGGCCGCTGATCGACGCGGGCATG
		9133-9167	ACGCCGGGCGGAC----GGCGCTGATCGACGTCGGCATG
**GMA1-D4**	35	27747-27778	CGAAGTTGACGGAGATCGGCG---AGGAAACGTTG
		17158-17192	CGAAGCTGAAGGAGATCGGCGGCCAAGACACGTTG
**GMA1-D5**	32	13862-13893	CTGTTCCAGATCGCGAAGGGCACGTGGACGTC
		13724-13755	CTGATCCAGAACCCGGACGGCACGTGGACGTC
**GMA1-D6**	29	14344-14372	GGCCCGCCACGACAAGGCCGAAGCCGAGA
		4813-4841	GGCCCGCAAGCACGAGGCCGAAGCCAAGA
**GMA1-D7**	25	31228-31252	TGAAAGGAAAGTCGCATGACCAGCA
		28614-28638	TGAAAGGAAATCAGCATGACCAGCA
**GMA1-D8**	25	35101-35125	GGCTGCACCGACTGGACGATCCGCA

Phage-Repeat number	Size (bp)	Coordinates	Sequence alignment
		20856-20880	GGCCGCACGGTCTTGACGATCCGCA
**GMA1-D9**	25	32218-32241	GCCCGGTCCG-TGTCGCGTGCCGTG
		30330-30354	GCCTGGTCTGGTGTCGCGTGCCGTG
**GMA1-D10**	24	6949-6972	TCGCCGCACCAACCACCGAAGTCG
		2819-2842	TCGCCGCAACCGCCACCGAAGTCG
**GMA1-D11**	23	17829-17851	GGTCGCGCGCGTCGTGGACGCGC
		7819-7841	GGTCGCGCGCGTGCTGGACGCGC
**GMA1-D12**	23	35475-35497	CCGACCATCCGCGCCGCCGACGG
		33952-33974	CCGACCATCCGCGCGTTCGACGG
**GMA1-D13**	20	25205-25224	TCGAGGTCGCTGTCGCCGTC
		21891-21910	TCGAGGTAGCTGTCGCCGTC
**GMA1-D14**	20	29617-29636	TGTCGCCGCCGCCCTCATCC
		11233-11252	TGTCGCCGCCGGTCTCATCC
**GMA1-D15**	20	38752-38771	GCAAGCTCCGTGACGAGCAG
		15051-15070	GCAAGCTCCGTGACACGCAG
**GMA1-D16**	20	38140-38159	GGTTCAAGGTCGACGACGGT
		17257-17276	GGTTCAAGGTCGGCGACCGT
**GMA1-D17**	19	36349-36367	CGGCGACGCCCGCACCGAA
		9468-9486	CGGCGACGCCCGCACCGAA
**GMA1-D18**	19	33587-33605	CGACCAGGCGATCGCCGCG
		5404-5422	CGACCAGGCGAACGCCGCG
**GMA1-D19**	19	12915-12933	TCGTCATCAACCCTGACGA
		12744-12762	TCGTCATCAACCGTGACGA
**GMA1-D20**	18	24932-24949	GACCAGGAACTACACGCG
		16382-16399	GACCAGGAACTACACGCG
**GMA1-D21**	18	41160-41177	CGCGACGCCTCGGCCTCG
		2603-2620	CGCGAGGCCTCGGCCTCG
**GMA1-D22**	18	14064-14081	CCGGCGGCGACCCCGGCA
		13794-13811	CCGGCGGCGACCCCGACA
**GMA1-D23**	18	37225-37242	GCTCGGCGACTCGTGCGA
		36965-36982	GCTCGGCGACTCGTACGA

Phage-Repeat number	Size (bp)	Coordinates	Sequence alignment
**GMA1-D24**	17	5758-5774	CGTCATCGACGAGTTCC
		5290-5306	CGTCATCGACGAGTTCC
**GMA1-D25**	17	32755-32771	CGCCGAGGCGGGTGAGG
		32534-32550	CGCCGAGGCGGGTGAGG
**GMA1-D26**	16	9173-9188	CACCCTCGCTGAGGCG
		5242-5257	CACCCTCGCTGAGGCG
**GMA1-D27**	15	33173-33187	CCGCGCCGCCCTCGA
		32-46	CCGCGCCGCCCTCGA
**GMA1-D28**	15	33173-33187	CCGCGCCGCCCTCGA
		152-166	CCGCGCCGCCCTCGA
**GMA1-D29**	15	33799-33813	GACGCCGTCGACGCG
		3223-3237	GACGCCGTCGACGCG
**GMA1-D30**	15	19873-19887	ACACCGTCACCGACG
		6335-6349	ACACCGTCACCGACG
**GMA1-D31**	15	10592-10606	TCCACCATCGGCATC
		10010-10024	TCCACCATCGGCATC
**GMA1-D32**	15	34043-34057	GTCGCCGTCGTGGAC
		17750-17764	GTCGCCGTCGTGGAC
**GMA1-D33**	15	34254-34268	GAGGAGTCGTGAAGT
		32547-32561	GAGGAGTCGTGAAGT
**GMA1-D34**	15	35154-35168	CGCCGAGCGCGCCGA
		33569-33583	CGCCGAGCGCGCCGA
**GMA1-I1**	47	22486-22531	CACGATGTCGGGGTCCGGTGGCGCATTC-CGCACGATGATCGTCGCC
		13816-13771	CACGATGTCGGGGT-CGCCGCCGGATTCGCGCTTGATGAGCGCCGCC
**GMA1-I2**	38	22006-22043	GGATGCGACGGTGTCACCGCCGGAGATGGTCGCGTTCG
		1459-1422	GGATGCGACGGTGCCGATGCCGTCGATGACAGCGTTCG
**GMA1-I3**	33	30408-30440	ACGTCGTCACCTGCGGCGTTCGCGAAGAGTGTC
		7024-6992	ACTTCCTCACCTGCGGCGTTGCGGACGAGTTTC
**GMA1-I4**	23	10499-10521	CAGGCCACCGCGGCCCTTCGTGC
		4401-4379	CAAGCCGCCGAGGCCCTTCGTGC
**GMA1-I5**	20	26623-26642	CGGTCACAGCCTCACCTCGC

Phage-Repeat number	Size (bp)	Coordinates	Sequence alignment
		20752-20733	CGGTCACAGCCTCAGCACGC
**GMA1-I6**	17	38627-38643	GCGCGAGACTTCCCGCC
		4038-4022	GCGCGAGACTTCCCGCC
**GMA1-I7**	16	28081-28096	GAAGGTTGGGCGCCCG
		23291-23276	GAAGGTTGGGCGCCCG
**GMA1-I8**	15	24463-24477	TCCGGTCGTCGTCGG
		2248-2234	TCCGGTCGTCGTCGG
**GMA1-I9**	15	26806-26820	GCCGCCGCCTGCGGG
		5119-5105	GCCGCCGCCTGCGGG
**GMA1-I10**	15	12764-12778	CTCGATGCGGCGTCG
		10794-10780	CTCGATGCGGCGTCG
**GMA1-I11**	15	25463-25477	GGATGCCGCGGCCGT
		21584-21570	GGATGCCGCGGCCGT
